# Artificial neural network, machine learning modelling of compressive strength of recycled coarse aggregate based self-compacting concrete

**DOI:** 10.1371/journal.pone.0303101

**Published:** 2024-05-13

**Authors:** P. Jagadesh, Afzal Hussain Khan, B. Shanmuga Priya, A. Asheeka, Zineb Zoubir, Hassan M. Magbool, Shamshad Alam, Omer Y. Bakather

**Affiliations:** 1 Department of Civil Engineering, Coimbatore Institute of Technology, Coimbatore, Tamil Nadu; 2 Civil Engineering Department, College of Engineering, Jazan University, Jazan, Saudi Arabia; 3 Green Energy Park (IRESEN, UM6P), km2 R206, Benguerir, Morocco; 4 Department of Chemical Engineering, College of Engineering, Jazan University, Jazan, Saudi Arabia; Amirkabir University of Technology (Tehran Polytechnic), ISLAMIC REPUBLIC OF IRAN

## Abstract

This research study aims to understand the application of Artificial Neural Networks (ANNs) to forecast the Self-Compacting Recycled Coarse Aggregate Concrete (SCRCAC) compressive strength. From different literature, 602 available data sets from SCRCAC mix designs are collected, and the data are rearranged, reconstructed, trained and tested for the ANN model development. The models were established using seven input variables: the mass of cementitious content, water, natural coarse aggregate content, natural fine aggregate content, recycled coarse aggregate content, chemical admixture and mineral admixture used in the SCRCAC mix designs. Two normalization techniques are used for data normalization to visualize the data distribution. For each normalization technique, three transfer functions are used for modelling. In total, six different types of models were run in MATLAB and used to estimate the 28^th^ day SCRCAC compressive strength. Normalization technique 2 performs better than 1 and TANSING is the best transfer function. The best k-fold cross-validation fold is k = 7. The coefficient of determination for predicted and actual compressive strength is 0.78 for training and 0.86 for testing. The impact of the number of neurons and layers on the model was performed. Inputs from standards are used to forecast the 28^th^ day compressive strength. Apart from ANN, Machine Learning (ML) techniques like random forest, extra trees, extreme boosting and light gradient boosting techniques are adopted to predict the 28^th^ day compressive strength of SCRCAC. Compared to ML, ANN prediction shows better results in terms of sensitive analysis. The study also extended to determine 28^th^ day compressive strength from experimental work and compared it with 28^th^ day compressive strength from ANN best model. Standard and ANN mix designs have similar fresh and hardened properties. The average compressive strength from ANN model and experimental results are 39.067 and 38.36 MPa, respectively with correlation coefficient is 1. It appears that ANN can validly predict the compressive strength of concrete.

## 1. Introduction

The progress of "contemporary" self-compacting concrete (SCC) is related to improving the concrete quality made in Japan in the late 1980s, where it was determined that incomplete and uneven compaction was the main cause of the unsatisfactory performance of concrete structures. Since there was no practical way to guarantee complete compaction of concrete on a site, attention shifted to finding a way to avoid having to compact concrete at all, whether through vibration or any other method. As a result, researchers Okamura and Ouchi from the University of Tokyo created the first practical SCC. That would include enhanced quality of the concrete and reduced on-site repairs, shorter construction durations, and reduced overall costs [1]. In the present scenario, the use of SCC has received much interest in terms of a wide area of application [2].

Concrete has dominated the construction material market since the 1900’s, accounting for almost 8% of global carbon dioxide emissions. Furthermore, the over-mining of natural coarse and fine aggregates, which includes the high quality of coarse aggregate and fine aggregates, has resulted in the scarceness of the resources and increase in the aggregate prices. On the other hand, the restoration and disposal of existing buildings result in the gathering of construction and demolition (C&D) waste, which consumes large amount of land resources in addition to the pollution effect. Researchers have established that the usage of recycled aggregate from the waste concrete obtained from C&D waste offers economic, environmental, and social benefits [3–5]. Recycled aggregates that are obtained have poor quality in terms of physical and chemical properties than natural aggregates. Recycled aggregate consists of attached cementitious mortar to it from parent concrete. This attached mortar consists of more pores as a result of partial or full hydration of the cementitious particles. This leads to a reduction in the density of recycled aggregates associated to the natural aggregates. Due to these pores and the presence of attached mortar absorb higher water absorption value than aggregates obtained from naturally. This pushes the researchers or academicians to propose mix design properly is essential to attain the required qualities for recycled aggregate concrete [2].

Recycled aggregate reduces the evacuation of natural resources and absorbs a significant amount of carbon dioxide (CO_2_) while being crushed into smaller particles. This reduces CO_2_ levels in the atmosphere. The need for new landfills for recycled aggregates is reduced and, most importantly, the reduction in costs is reported. The presence of pores in the recycled aggregate and the attachment of mortar leads to an increase in the water absorption capacity in the range of 3% to 9%. An increase in the water absorption results in an increase in the segregation and bleeding of the fresh concrete properties blended with recycled aggregates. Thus, results in the degrades in the concrete quality in terms of mechanical and durability properties. A drop in the concrete compressive strength of about 15% minimum is noted in the literature [6–8]. The effect of the recycled aggregates on the SCC fresh concrete properties is reported by various literatures [8–11].

The presence of recycled aggregates results in the variation of the density and Young’s modulus of recycled blended concrete responds differently compared to conventional concrete when it is subjected to dynamic excitations [12]. On 100% of the replacement of natural aggregate by recycled aggregate, the density of hardened concrete can be reduced by more than 7%, and a reduction in the modulus of elasticity of about 45% compared to conventional concrete is noted. Structural stiffness and mass of the concrete play a vital role towards the structural dynamic natural frequency. Hence prediction of the dynamic response of recycled aggregate concrete is difficult compared to conventional concrete is reported by Upshaw et al., 2021 [12]. The relationship between the compressive strength and modulus of elasticity, which is directly proportional for recycled aggregate, is also noted. There is no much difference (variation below 6%) in the modulus of elasticity of recycled aggregate concrete with and without high fineness of fly ash is reported by Tangchirapat et al., 2010 [13].

There is a significant increase in weight loss on the drying of recycled aggregate about 3.71%, compared to that of natural aggregate about 2.97%, which indicates that there are more pores in their surface. The specific gravity of natural aggregate is about 2.62, whereas the recycled aggregate is about 2.38 under saturated surface conditions, indicates that the recycled aggregate possesses lower density. Water absorption of the recycled aggregate about 1.56%, compared to the natural aggregates of about 0.30% indicating that there are different sizes (micro or nano pores) in the surface of recycled aggregates. From this, it can be concluded that the main factors like pore sizes and connectivity of pores apart from the nature of the material, influence the specific gravity and water absorption of recycled aggregates. One of the advantages of using lightweight composites materials for construction is to control building temperature and moisture, as reported by Charai et al., 2023 [14]. The water absorption of all recycled aggregates is always greater than that of natural aggregates, is reported by Purushothaman et al., 2015 [15].

Microscopic studies on the recycled aggregates indicate that the aggregates are a heterogeneous, irregular, and inconsistent microstructure. The surface of recycled aggregate suggests that the uneven surface is more porous than the surface of the old mortar attached to it. There are two types of Interfacial Transition Zone (ITZ) noted concerning the recycled aggregates reported in the literature. First, the ITZ is formed between the new aggregates to the new binder material and the second ITZ is formed between the recycled aggregates and the new binder material [4]. The presence of high fine content (cementitious materials and admixtures) in the SCC, as well as the increased use of superplasticiser (SP), results in SCC mixes are more expansive than conventional concrete. The application of processing of mineral admixture results in the enhanced fresh and hardened properties of concrete [16]. To compensate for this drawback, recycled aggregates can be combined with SCC mix without compromising the concrete properties. Recycled aggregates used in the literature are in the form of recycled fine aggregates and recycled coarse aggregates. Even the usage of recycled materials leads to circular economic policies in the construction sector as reported by Jagadesh et al., 2023b [17].

The chemical and physical properties of recycled aggregates are typically lower than those of NA. The use of RCA in SCC will be concerned not only with its strength and structural properties but also with its durability [18]. SCC uses less manpower, constructs buildings more quickly, has greater durability, doesn’t require a vibratory machine, and doesn’t bleed and segregate. However, because there is no standard design mix, measuring must be done accurately, and numerous trial batches and laboratory tests are necessary. Because of its high fluidity, it may cause hydrostatic pressure problems in mould workmanship. It now costs more than normal concrete. Hence, it is necessary to estimate the properties of SCC from mix proportions are required and, in the literature, there are several techniques like Artificial Neural Network (ANN), Machine Learning (ML), and Deep Learning (DL) are reported.

One of the most common approaches to predict the concrete properties followed in the literature is ANN. Even several literatures [19–21] show the prediction of strength properties of SCRCA using ANN and ML. In literature, several input variables used are cementitious content, water content, mineral admixture content, chemical admixture content, fine aggregate content, coarse aggregate content, recycled coarse aggregate content, and recycled fine aggregate content, whereas the output variables used are compressive strength and spilt tensile strength of SCC. In order to even distribute the data, normalisation techniques are adopted to get the best model. Several sensitive assessment techniques are used to assess the performance of the model.

Neurons in the ANN model behaves similarly to the biological structure of neurons in the human brain. A neural network consists of a number of layers that are multiple in nature and these layers are interconnected either in nonlinear or linear processing, which work in parallel. Multiple inputs from every neuron in each processing unit in the layer to which it is linked using weighted parameters achieve suitable computation, and communicate its output variables to the processing units using the transfer function [20]. To build an effective ANN, it requires three critical steps must be considered: first architecture network, training and testing. The number of processing units in each layer, the pattern of connectivity and the transfer function used for every processing unit are the basic features of the architecture used for the network. Supervised behaviour or unsupervised behaviour of the training procedure are used for the ANN model. From each data set from input, the expected output can be achieved with the help of different sets of training data set in the network is known as supervised training. Whereas, the dataset from input, the neural network must learn the similarities and regularities among the training patterns is known as unsupervised training.

The importance of a measurable assessment of recycled aggregates in the construction industry is deeply affected by national policymaking for the authorities. When the measurable database in the construction industry is defined more, it can excite government regulation programmes by enacting suitable recycled aggregate polices. With help of different construction sites, different data collected are complex in the nature, and the ANN model makes a precise tool for predicting the models [22]. Development of mix design for the concrete mix with the help of an artificial neural network is already reported in the literature [23,24]. In recent literature, the application of machine learning and ANN for the SCRCAC to predict the compressive strength and spilt tensile strength [25–28]. Early assessment of the SCC’s compressive strength is crucial for design and application in ready-mixed concrete plants and construction sites.

Given that strength is determined experimentally through destructive tests. The different ratio from the mix design parameters or even directly the quantity of the mix design parameters from SCC mix design will affect the strength characteristics of the SCC. Apart from the above parameters, the quality of ingredients, method of specimen testing, size of the specimens according to different standards, etc., plays a vital role in the prediction of the strength parameters. In order to save the environment, reduce the economic cost, decrease the energy associated with manpower, etc., there is a need for some new techniques to predict the required parameters for the concrete. In this study, an attempt is made to compare the strength parameter from the predicted results using different ML techniques and the mix design from the international standards. The aim of this study is to forecast the compressive strength of SCC with recycled coarse aggregate by using ANN. Furthermore, the investigation was conducted experimentally on the 7^th^ and 28^th^ day compressive strength of SCRCAC to compare the results from the ANN model. The compressive strength of SCRCAC is used as output based on the precision of the input data from the mix design (literature) and how the ANN model is trained. To achieve the required output, the sample of SCC mixes data were collected from the literature.

## 2. Methodology

Mix designs are derived from the components to arrive at the compressive strength of SCRAC. In this research, there are two phases. The first phase is to develop the model to estimate the 28^th^ day compressive strength. Nowadays, most researchers are applying ML methods like ANN and other methods like Random Forest (RF), Extra trees (ET), Extreme gradient boost (XGB), and Light gradient boosting machine (LGBM) in assessing the compressive strength of SCRAC. Different normalisation methods and transfer functions are used in the present study to estimate the compressive strength of the mix components. In this research, an effort is made to forecast the compressive strength of SCRAC at 28^th^ days by ANN methods. 70% of the data is used to predict the model, and 30% of the data is used to authenticate the ANN model. In order to make the model easily accessible to both technical and non-technical persons, the models are represented as general equations.

**Fig 1:**
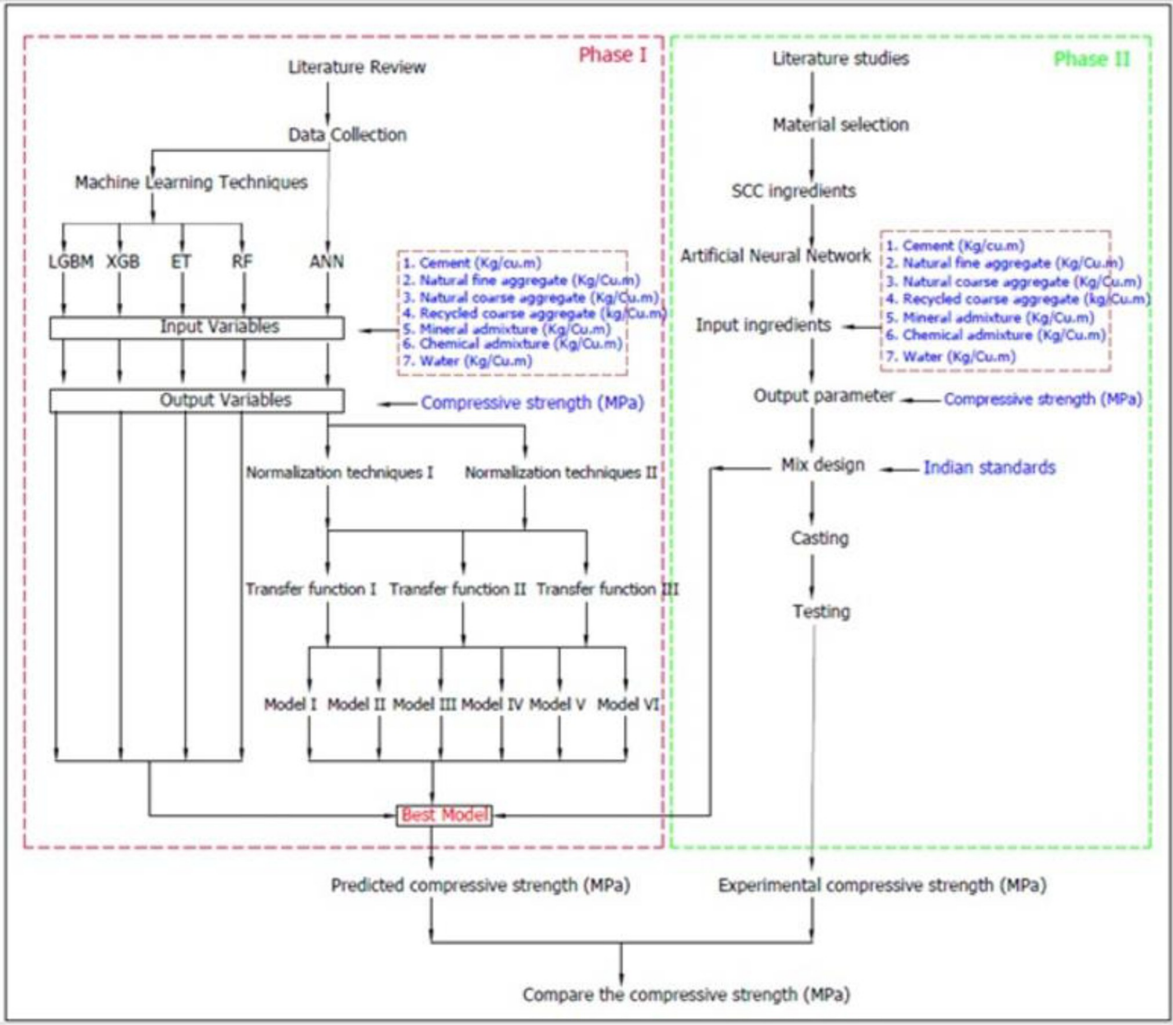
Methodology for the current research.

In the second phase, Indian code is used to develop a mix design for the SCCRAC from the ingredients. Ingredients used in these investigations are cement content, natural coarse aggregate, natural fine aggregate, recycled coarse aggregate, water, mineral admixture and SP, which are input parameters for the first phase. The standard parameters used are minimum, maximum cement content, water cement ratio, coarse aggregate content, etc. The Methodology for the current investigation is shown in Fig 1.

## 3. Modelling

### 3.1 Database for modelling

**Fig 2:**
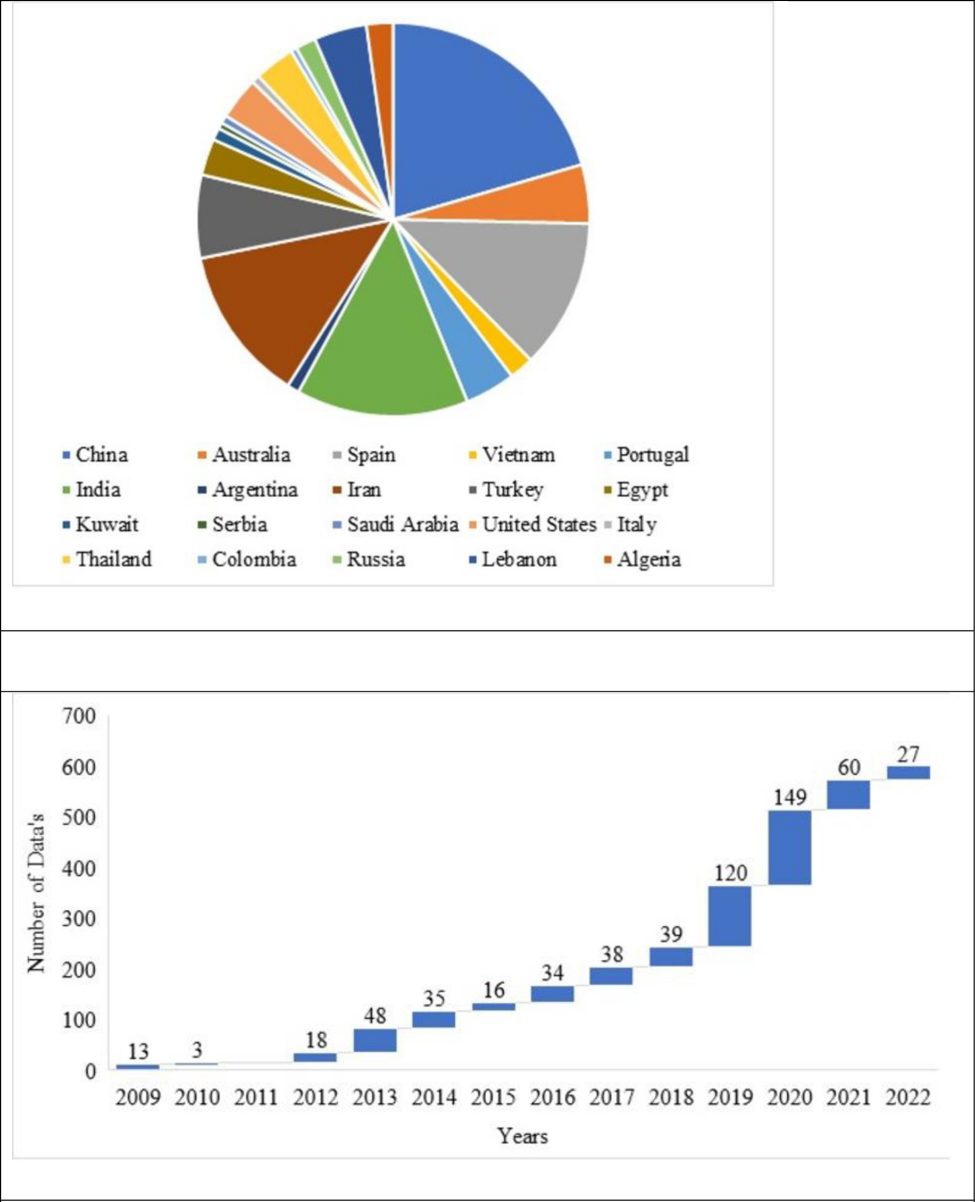
(a). Data collected from various countries from literature. (b). Number of publications with respect to a number of mix data for the past decade.

The experimental database arrived from 61 literature from the past 14 years publications [10,18,29–78] and the distribution of the database across the various countries based on the country is shown in Fig 2A. It is noted that the number of data to use the RCA in SCC is used maximum by China; next to it are India and Iran, is noted in Fig 2A. Most Asian countries have started their research on utilising the RA on the SCC, is reported in the literature. Next to Asian countries, some of European countries like Spain, Portugal, etc. have started using RA on SCC is noted from the literature. Most of the research was done in 2019 and 2020 is noted in Fig 2B.

### 3.2 Visualization of data by frequency distribution

**Fig 3:**
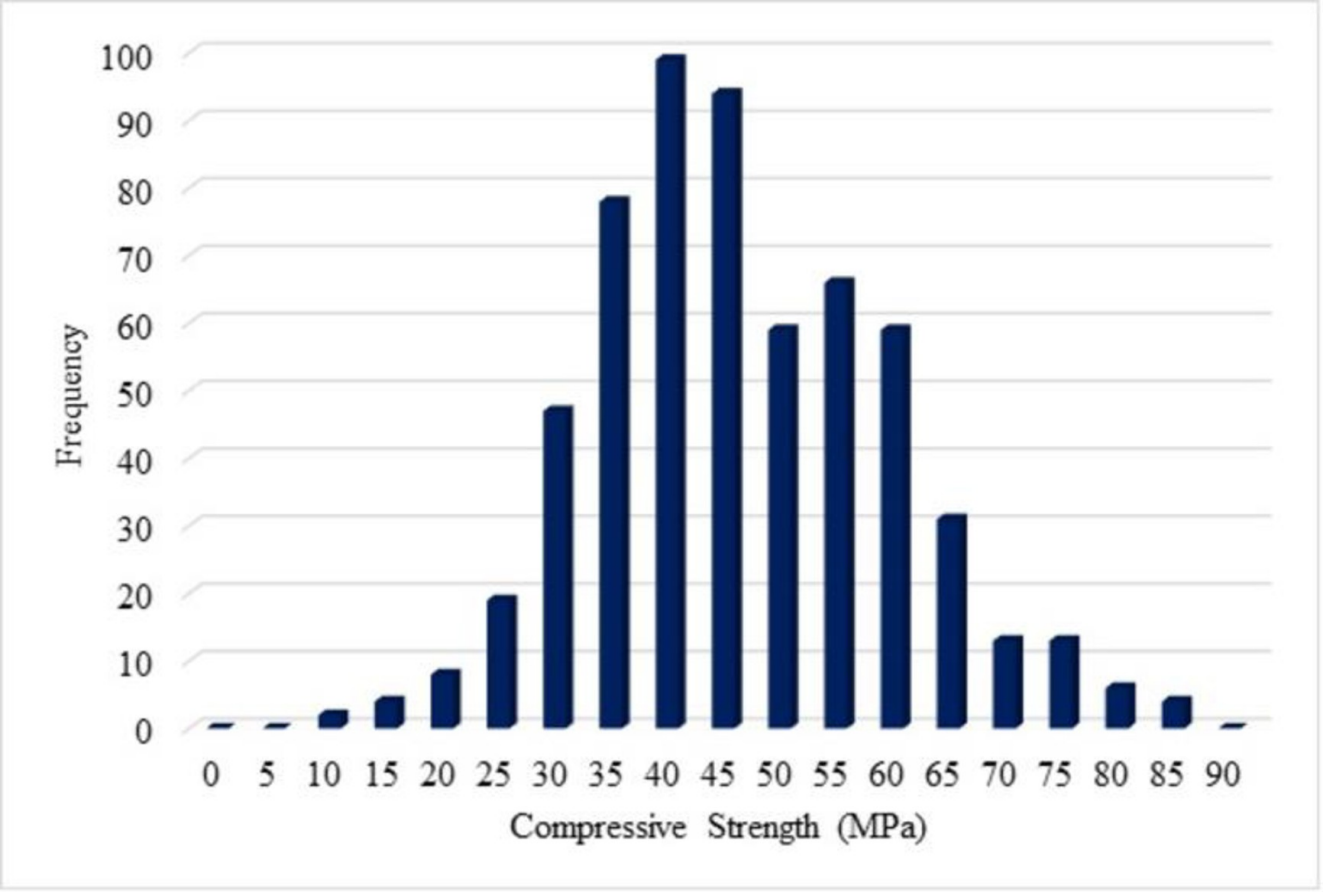
Histogram for compressive strength (MPa).

**Fig 4:**
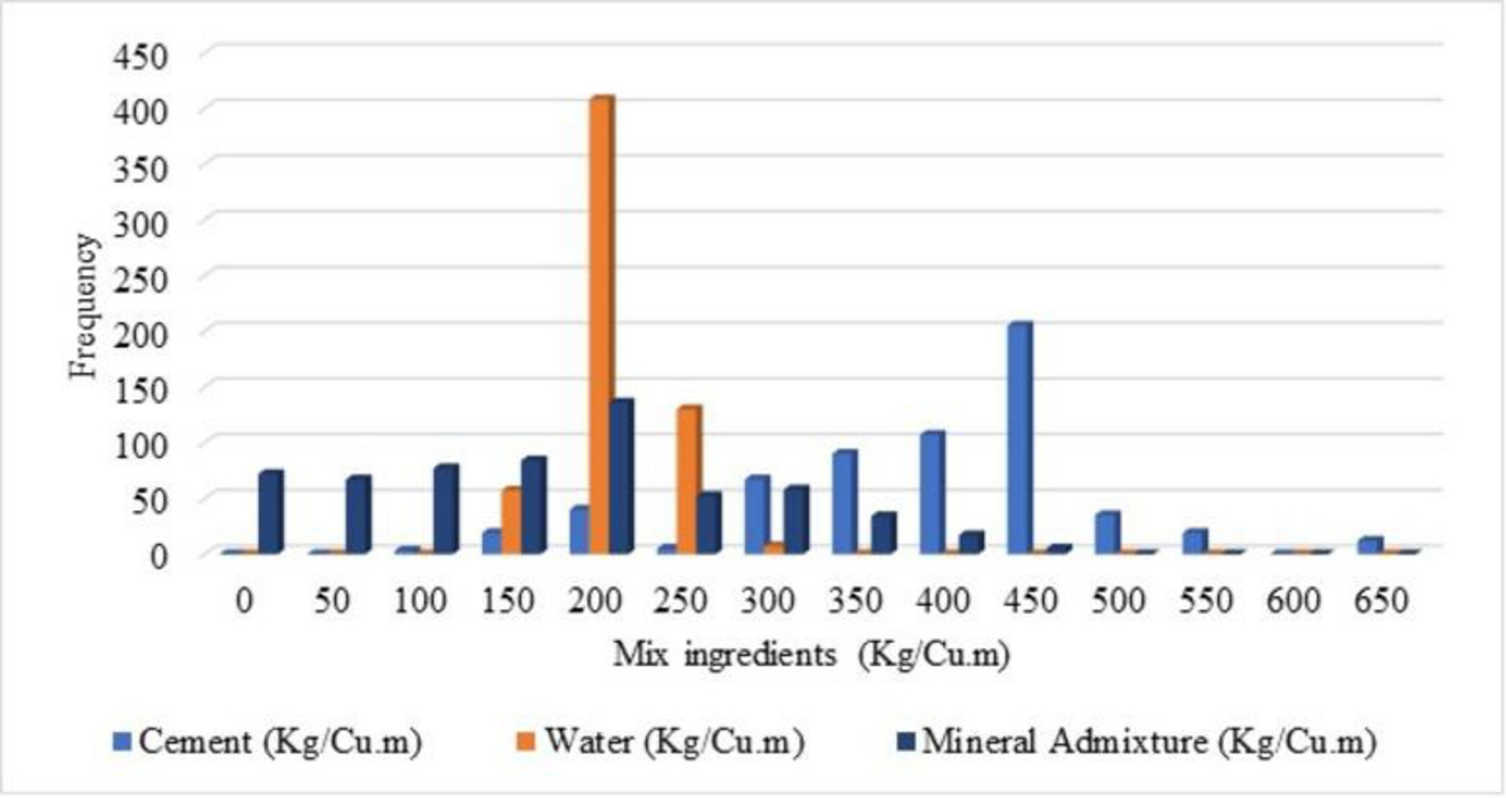
Histogram for cement (Kg/Cu.m), water (Kg/Cu.m) and mineral admixture (Kg/Cu.m).

**Fig 5:**
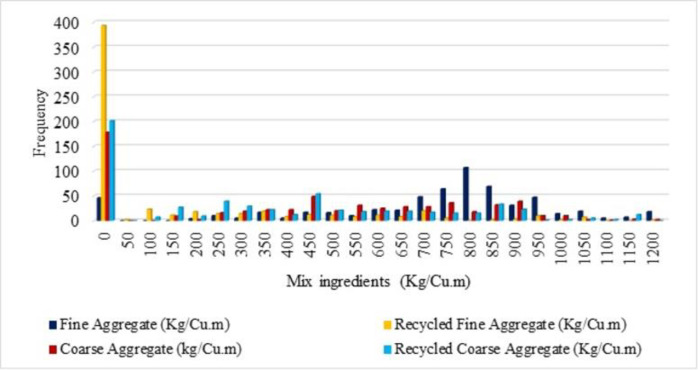
Histogram for aggregates (Kg/Cu.m).

**Fig 6:**
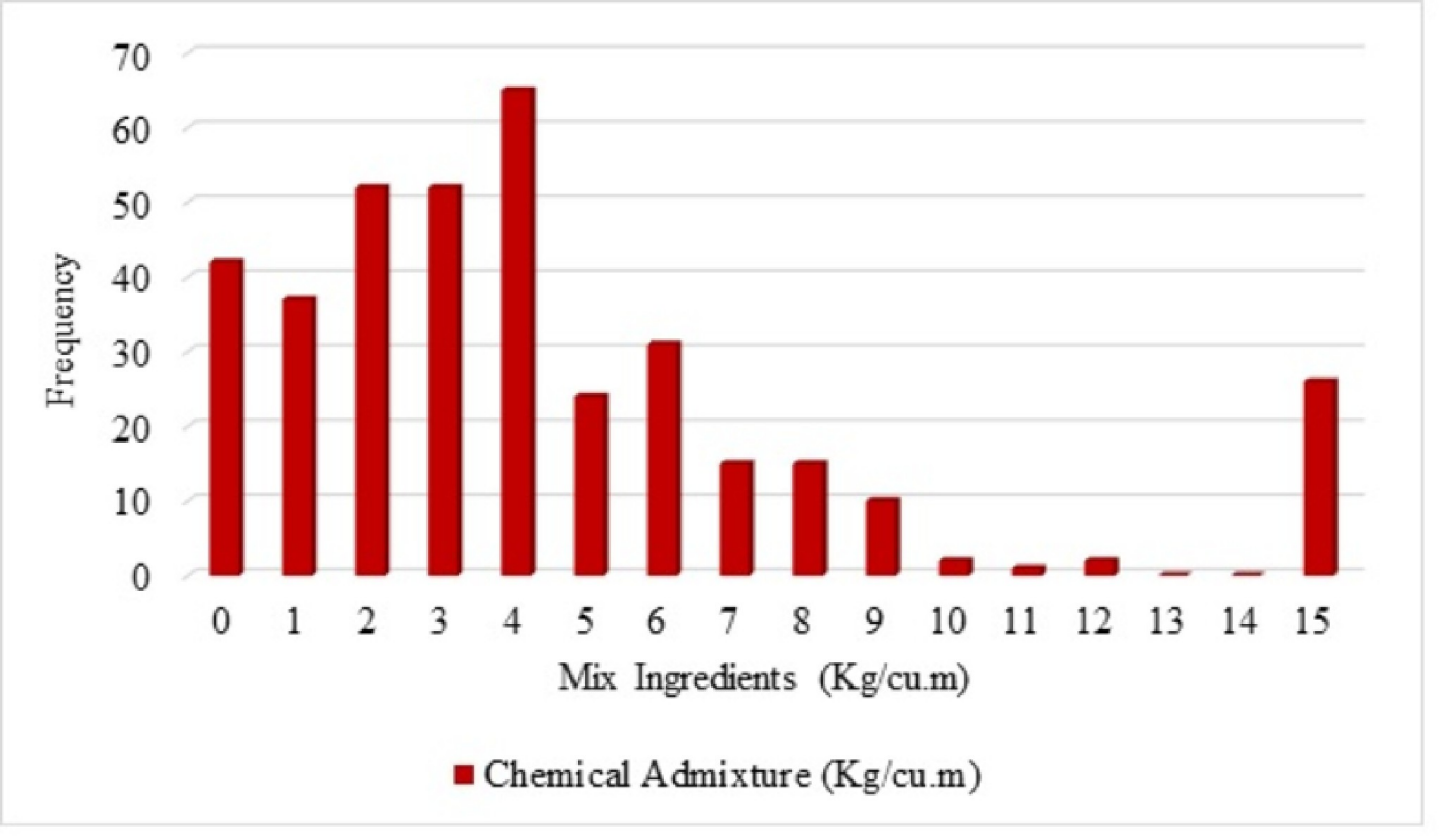
Histogram for chemical admixture (Kg/Cu.m).

Visualization of the input data is simply distributed as frequency distribution is depicted in this section. The frequency of spreading of the variables (input and output) is explained in terms of histogram figures in order to understand the distribution of the frequency of the variables. A number of frequencies for compressive strength lie between 90 to 100 is noted for the compressive strength 40 to 45 MPa is shown in Fig 3 from the histogram figure. From Fig 4, it is noted that the frequency of water as 400 for the mix ingredients as 200 Kg/Cu.m. For cement, the number of highest frequencies is noted as 400, and for the mix ingredients, 450 Kg/Cu.m, and the mineral admixture, the number of highest frequencies is noted as 100, for the mix ingredients as 200 Kg/Cu.m. From Fig 5, the histogram figure for aggregates, including the natural and fine aggregates, is depicted with frequencies with the highest for natural fine aggregate in the range of 800 Kg/Cu.m. For the natural coarse aggregate, the frequencies are highest as 450. Whereas the recycled fine and coarse aggregate, the number of frequencies below 100 is observed. The highest number of frequencies for the chemical admixture is noted above 60, with chemical admixture as 4 Kg/Cu.m noted in Fig 6. Similar visualisation of the collected data variables is reported in the literature [19–21]. The data variables show that the distribution of the number of data is distributed with different ranges of frequencies of variables.

### 3.3 Artificial neural network

Forecasting of SCC compressive strength from the data’s collected from the literature using ANN and other machine learning is reported from the literature [19]. The input layer had eight parameters: cement quantity, water quantity, natural fine aggregate, natural coarse aggregate, recycled coarse aggregate, mineral admixture, and chemical admixture. The output layer entailed of 28^th^ day compressive strength of SCC. While developing the ANN model, 70% of the samples were used for training and 30% for testing. ANN is an artificial tool to process the input information to predict the output variable, similar to the human biological nervous system used. Most of the ANN tools are used to forecast the output variables, which is used as a decision-making tool in various fields. ANN consists of a number of interconnected neurons using processing elements called layers, which work together to solve the given problems [20,21]. ANN consists of three different layers processed with the help of different neurons. (1) Input layer consists of a different number of input variables (2) Hidden layer is used to analyse the different number of variables from the input (3) Output layer is used to generate the output variables.

### 3.4 Normalisation techniques

Mapping of the collected data from the different sources is used per the required structural representation; normalisation methods are adopted. The data from the different sources are transformed as per the required arrangement before analysis, which is used for the further process. The normalisation method is a mapping method or a scaling method in the preprocessing stage to map or scale the input variables within a required range from an existing range from the source. Transformation of the input variables within a range of similar scales improves the performance of the training stability of the predicted model. In order to reduce the difference between the output variables from the sources, input variables are required to be normalised. There are two normalisation techniques adopted in this study to scale the variation in collected variables (Input and output variables). Two techniques are used to find out the optimised normalised technique, which is used in the literature. Normalisation is the procedure of casting the data to a specific range, between 0 and 1 or between -1 and +1 [79]. The first normalisation technique was used to scale the data between 0 to 1 as shown in Eq (1).


$$X_{i}=\frac{\left[X-X\right]}{\left[X_{max}-X_{min}\right]}$$ (1)

The second normalisation technique is used to arrange the data in the range between -1 and 1 using Eq (2) [80]. In order to eliminate the overfitting of the predicted value from the model, the dimensional consistency of the parameters needs to be maintained.


$$X_{i}=\frac{\left[2[X-X_{min}]\right]}{\left[X_{max}-X_{min}\right]}-1$$ (2)

Where Xi denotes the normalised data, Xmax represents the minimum value of X, Xmin represents the maximum value of X and X designates the experimental data. With help of Microsoft excel program, the data normalisation was performed for both techniques.

### 3.5 K-Fold cross method

K- fold cross method is used to assess the better group of sub-sets from the collected data from the literature. K—fold cross validation is a famous method used in artificial intelligence methods, including ML methods deep learning methods, and statistical modelling to analysis the performance and generalisation of forecasting models. It reports the challenge of quantifying how well a forecasted model will execute on the unseen dataset by simulating the forecasted model performance on various multiple subsets of the collected data. K–fold cross validation is defined as a process in which the dataset is divided into equal k-sized folds or subsets [81]. The model is trained for minimum K-3 folds or subsets, and the remaining subsets or folds are used for the testing of the model. The performance of each fold is analysed using sensitive assessment for every trained and testing dataset. K–fold cross method is used to detect the underfitting or overfitting of the predictable data from the estimate model. Hence, K–fold cross method is used to analysis the optimum dataset to get the best combination of datasets using a sensitive method. K–fold cross method used in this study is K = 10, for training and testing the dataset is depicted in **Table 1**.

**Table 1:** K–Fold method for optimizing the data sets.

**Training**	**K = 1**	**K = 2**	**K = 3**	**K = 4**	**K = 5**	**K = 6**	**K = 7**	**K = 8**	**K = 9**	**K = 10**	**K = 11**	**K = 12**	**Subsets or folds**
	**1**	**10**	**10**	**10**	**10**	**10**	**10**	**10**	**10**	**10**	**7**	**6**	
	**2**	**2**	**9**	**9**	**9**	**9**	**9**	**9**	**9**	**6**	**6**	**5**	
	**3**	**3**	**3**	**8**	**8**	**8**	**8**	**8**	**5**	**5**	**5**	**4**	
	**4**	**4**	**4**	**4**	**7**	**7**	**7**	**4**	**4**	**4**	**4**	**3**	
	**5**	**5**	**5**	**5**	**5**	**6**	**3**	**3**	**3**	**3**	**3**	**2**	
	**6**	**6**	**6**	**6**	**6**	**2**	**2**	**2**	**2**	**2**	**2**	**1**	
	**7**	**7**	**7**	**7**	**1**	**1**	**1**	**1**	**1**	**1**	**1**	**8**	
**Testing**	**8**	**8**	**8**	**3**	**4**	**5**	**6**	**7**	**8**	**9**	**10**	**10**	
	**9**	**9**	**2**	**2**	**3**	**4**	**5**	**6**	**7**	**8**	**9**	**9**	
	**10**	**1**	**1**	**1**	**2**	**3**	**4**	**5**	**6**	**7**	**8**	**7**	

### 3.6 Machine learning models

#### 3.6.1 Random forest (RF)

RF Regressor is an ensemble learning technique that processes by creating the average prediction for the individual trees while training various decision trees. It’s a powerful technique for regression tasks due to its robustness, particularly against overfitting by training multiple trees [79]. RF consists of three main categories, which include the assembling of trained regression trees via dataset for training, estimation of the average value of single regression tree outcome, and the validation of forecasting results. The collected trained set is used to estimate a new trained dataset comprising boot strap data. In the next step, a few data points are removed and swapped with the present data points. In the last step, the regression function is estimated for balanced data points. These processes are repeated till the achievement of the required forecasting value [81].

#### 3.6.2 Extra trees (ET)

Similar to RF, the ET Regressor is an ensemble learning technique that incorporates an increased degree of randomness. Instead of seeking for the optimal split, it arbitrarily selects the splits for each feature during the construction of the decision trees; this results in increased variance but decreased bias. This method increases the speed and effectiveness of ET for vast datasets. Its utilization of multiple trees to average predictions, like RF, contributes to the achievement of more reliable and accurate results. Regression and classification problems can be handled by ET. Every output in the decision tree is used for the classification problem, and the output of final data are averaged and the average of the output of all decision trees are used as the final output [82]. ET includes the following strategies: (a) every decision tree is created using all the samples in the dataset used for training but randomly selects the characteristics; (b) For each non-constant characteristic contained in the node, an arbitrary number in between the minimum and maximum of the characteristics is selected randomly [82].

#### 3.6.3 Extreme gradient boosting (XGB)

XGB Regressor is an improved technique of gradient boosting, created for speed and efficacy. Due to its exceptional performance, it is well recognized within the machine learning community. XGB enhances model accuracy by adding trees consecutively, each correcting errors left by prior ones, through strategic data splits to minimize prediction errors iteratively [83]. Additionally, XGB incorporates advanced regularization techniques designed to prevent overfitting, a common issue in machine learning models. This, in addition to its ability to efficiently handle sparse data and missing values, demonstrates XGB’s adaptability and robustness across a wide variety of data scenarios.

#### 3.6.4 Light gradient boosting machine (LGBM)

LGBM is a powerful gradient boosting framework designed specifically to handle large data sets with optimized speed and performance. In contrast to level-wise tree growth, it selects the leaf with the greatest delta loss to develop, resulting in enhanced accuracy, faster learning, and reduced memory use. Decision trees are used for the learning algorithm for the LGBM [84].

### 3.7 Performance assessment

Sensitivity assessment is defined as the assessment procedure to understand the uncertainty in the predicted output to the experimental output in a given mathematical model or other models derived [85]. This analysis is also necessary to assess the performance of the predicted results compared to the actual results with the help of different parameters like the coefficient of determination (R^2^), root mean square error (RMSE), mean square error (MSE), etc., If there are too many input variables are used to estimate the output variables with help of any statistical or ML approach, sensitive analysis is essential. Sensitivity assessment forecasts the effect of the different independent input variables on the particular dependent output variable [20,21]. In this study, the sensitive analysis was performed with the help of various indicators like R^2^, RMSE and MSE. Eqs (3)–(5) are used to estimate the performance of the model.


$$R^{2}=1-\left[\frac{\sum_{i=1}^{N}{\left(O_{i}-P_{i}\right)}^{2}}{\sum_{i=1}^{N}{\left(O_{i}-P_{i}\right)}^{2}}\right]$$ (3)

$$MSE=\sum_{i=1}^{N}\left[\frac{{\left(O_{i}-P_{i}\right)}^{2}}{N}\right]$$ (4)

$$RMSE=\sqrt{\sum_{i=1}^{N}\left[\frac{{\left(O_{i}-P_{i}\right)}^{2}}{N}\right]}$$ 5

Where Oi is the actual value used for the model, Pi is the estimated value obtained using the model and N is the total number of observed samples

## 4. Experiment work

### 4.1 Materials

#### 4.1.1 Cement (OPC 53 grade)

Cement, in broad terms, is any type of adhesive ingredient, especially it refers to the binding material in the construction industry. One of the common binding materials used in construction is cement, which is finely powdered content when mixed with water, which solidifies to form a solid mass. As a result of cement hydration, small crystals or a gel-like substance with a larger surface area are noted. Calcium, silica, iron and alumina compounds are major components, there are trace levels of other components observed. In order to regulate the fineness, specific gravity, soundness, setting time, and strength of the cement, the Ordinary Portland Cement (OPC) grade of 53, as per IS 8112–2004 [86]. The consistency test is used to figure out how much water is needed to make cement pastes for other testing. It can be said that specific gravity is crucial in defining the quality and compressive strength of the concrete mix. Le-Chatelier’s Flask method is used to calculate cement’s specific gravity in accordance with the Le-Chatelier Principle. The specific gravity of OPC cement is 3.15 as per IS 1727–2004 [87]. Normal consistency of cement is measured as per IS 4031 (part-4):1996 [88]. The period between the water added to the cement and the needle penetration is called the initial setting time, obtained as 35 minutes. The time taken between the water being added to the cement and the needle makes an impression on the cement mortar surface while the attachment fails to do so, which is noted as the final setting time, which is obtained as 178 minutes (IS-4031:Part-5,1998) [89]. The chemical properties of OPC used in this study are depicted in **Table 2**.

**Table 2:** Chemical properties of OPC 53 grade.

Chemical composition	Percentage of oxides (%)
Silicon dioxide (SiO_2_)	19.35
Calcium oxide (Cao)	68.64
Magnesium Oxide (MgO)	1.39
Sodium oxide	0.47
Aluminum Oxide (Al_2_O_3_)	4.57
Sulfur Trioxide (SO_3_)	1.23

#### 4.1.2 Fly ash

Burning pulverised coal in power plants for electricity production is a fine powder called fly ash. The mineral impurities in the coal combine as they exit the combustion chamber, then cool and harden to form this residue, which is referred to as fly ash. Fly ash transforms into a substance resembling OPC when mixed with lime and water. Fly ash increases the concrete’s strength and segregation when added to concrete mixtures, and it also makes the concrete simpler to pump. Class F fly ash is extracted from bituminous products which anthracite coals and consists of aluminium silicate glass, with quartz, mullite, and magnetite also present [84]. The specific gravity of the cement used in this study is 2.20 as per IS 1727–2004 [87].

#### 4.1.3 Manufacturing sand

Because of the rapidly growing construction industry, the claim for sand has skyrocketed, resulting in a scarcity of appropriate river sand in most parts of the world. In order to save environmental resources, construction industrialists have started using manufacturing sand nowadays increased drastically. Hence, in this study, on behalf of the natural fine aggregates, manufacturing sand is used for mix which fills the voids in between the coarse aggregates. For SCC, the powder content should be used to increase the fluidity of the mix. One of the major components in the powder content is fine aggregates, which resist shrinking and cracking. Manufacture sand is obtained from the local Coimbatore region and their properties are depicted in **Table 3** as per latest Indian standards.

**Table 3:** Physical properties of manufacturing sand.

Property value	Property value
Fineness Modulus	2.57
Bulk Density (kg/cm^3^)	1850
Water Absorption (%)	0.45
Specific Gravity	2.75

#### 4.1.4 Natural coarse aggregate

Coarse aggregates are obtained from the broken stones of granite and limestone, which plays a vital role in the stability of the concrete. In order to evaluate the quality of the coarse aggregate, various tests like fineness modulus, specific gravity, and water absorption were carried out as per Indian standards like IS: 2386 (Part III)– 2002 [90]. The properties of coarse aggregate are listed in **Table 4**.

**Table 4:** Physical properties of natural coarse aggregate.

Property value	Property value
Fineness Modulus	4.57
Water Absorption (%)	1.002
Specific Gravity	2.66

#### 4.1.5 Recycled coarse aggregate

C&D waste is collected from the construction industry, which is processed to collect the RCA. This kind of RCA provides a lot of advantages like economic benefits, environmental benefits, etc., The Quality of natural coarse aggregates is generally found to be lower quality than the RCA. RCA has many pores in the surfaces, which provides lesser density compared to the natural coarse aggregates [4]. Several properties similar to the natural coarse aggregates are performed for the RCA, and the results are depicted in **Table 5**.

**Table 5:** Physical properties of RCA.

Property value	Property value
Fineness Modulus	4.57
Water Absorption (%)	4.47
Specific Gravity	2.52

#### 4.1.6 Superplasticiser

SP is used as an additive in high-strength concrete production which is used as high-range water reducers. SP are chemical compounds that allow SCC to be made with approximately 15% less water. SP can reduce water content by 30% or more. Chemical admixtures are added to concrete to improve its flowability, reduce the amount of water in the SCC, and improve the strength and durability of concrete. AURACAST 250M is used in this study. The physical properties of SP used in this study are listed in **Table 6**.

**Table 6:** Physical properties of SP.

Property value	Property value
Appearance	Light brown liquid
pH	Minimum 6.0
Volumetric mass @ 25°C	1.05 to 1.09 (kg/litre)
Workability	2 hours and more depending on the dosage

#### 4.1.7 Water

The water used is free of impurities such as organic matter, suspended solids, and dissolved salts, which can have an adverse effect on the concrete properties, particularly the fresh and hardened properties of concrete. If the percentage of water used is lower, there will be insufficient water to hydrate cement. Cement and concrete will have less mechanical properties due to being weak and porous. In site conditions, water is added too much to improve the mix’s workable nature, which leads to poor concrete or mortar. With a higher water content in the mix, coarse aggregates lead to segregation, resulting in the concrete being porous and having poor mechanical properties. A certain amount of water is required to hydrate the cement completely. More water is required to make the concrete workable enough to be placed.

### 4.2 Methods

#### 4.2.1 Mix design

Mix design of concrete is the process of estimating the appropriate amount of concrete ingredients and the necessary ratios with the target of making concrete with a minimum level of strength and durability while using the least resources. Mix proportions for the SCC used in the study are calculated as per IS 10262:2019 [91], and the mix ingredients are shown in **Table 7**.

**Table 7:** Mix proportion for self-compacting concrete with RCA.

Mix ID’s	Cement (kg/m^3^)	Fly ash (kg/m^3^)	Water (kg/m^3^)	Fine Aggregate (kg/m^3^)	Coarse Aggregate (kg/m^3^)	Recycled Coarse Aggregate (kg/m^3^)	Chemical Admixture (kg/m^3^)
ET1	287	155	190	975	775	0	2.65
ET2	280	151	185	970	770	0	2.59
ET3	294	159	195	980	780	0	2.72

#### 4.2.2 Experiment

*4*.*2*.*2*.*1 Fresh concrete properties*. To determine its suitability, several tests were carried out, Slump flow, V funnel, etc., and harden properties like compressive strength test. The property of freshly mixed concrete, known as "workability of concrete" determines how easily and uniformly it can be assorted, positioned, consolidated, and finished. Maximum strength cannot be achieved simply by using the ideal water-cement ratio. Concrete must be completely compacted to reach its maximum strength. Air voids caused by insufficient compaction have a detrimental impact on strength. Influencing factors for workability water content, mix proportion, aggregate size and shape, use of admixtures, aggregate grading, and aggregate surface texture. Concrete’s workability can be evaluated using a variety of fresh concrete tests for the SCC, like V funnel, slump flow, U flow, and L box tests. Workability properties as per EFNARC standards are represented in **Table 8**. Each test was conducted based on the mix design discussed above, and its fresh properties were evaluated.

**Table 8:** Workability properties of SCC.

Property	Test	Range
Filling ability	Slump flow	SF1-550-650 mm SF2-650-750 mm SF3-750-850 mm
Passing ability	L box test	0.8–1.0
Filling ability	V funnel	VF1- <8s VF1- 9s-25s
Passing ability	U box	0–30 mm

#### Slump flow

In the absence of obstructions, the horizontal free flow of SCC is evaluated using the slump flow test. It was initially created in Japan to be used in the evaluation of underwater concrete. The test also reveals segregation resistance. The standard slump cone is used, which has a 200 mm diameter base, 100 mm diameter at the top, and a 300 mm height. A square base plate that is rigid and must have sides that are at least 700 mm long. About the center point, where the slump cone is to be placed, concentric circles are marked. 500 mm in diameter is used to draw a firm circle. The test requires approximately 6 liters of concrete, sampled normally. Keep the base plate stable in nature and level the surface and the slump cone in the center, holding it down firmly. The Interior of the cone and the base plate used are moistened with the help of oil. With the help of the scoop, the slump cone is filled with concrete, without tamping, concrete is filled to the top level of the slump cone. Clear the area around the cone’s base of any extra concrete. B raising the slump cone vertically, the concrete flows out freely. After measuring the concrete’s final diameter in two perpendicular directions, calculate the average of the two diameters. This is the mm-scale slump flow.

#### V Funnel

Apart from the flowability characteristics of the SCC, there are several characteristics that influence the fresh concrete properties of SCC. One of the potential liabilities of SCC characteristics is the blocking nature of the concrete, which will be measured in terms of an inverted cone shape. The ability of the SCC with a maximum coarse aggregate size of 20 mm is fill on the required shape is measured using V-funnel test. Around 12 liters of SCC are poured into the funnel, and the amount of time it takes for the concrete to pass through the required apparatus is quantified. After that, the concrete funnel can be replenished and given five minutes to settle. The flow time will increase noticeably if the SCC exhibits segregation.

#### L Box Test

The test evaluates both the SCC ability to flow and the degree to which it is jammed by reinforcement. The tool is a rectangular box with a "L" shape to it, divided into vertical and horizontal sections by a gate, which is movable and to which a vertical reinforcement bar is attached. The test required about 14 liters of concrete, sampled normally. The sliding gate in the apparatus can open freely before aligning the apparatus on the ground and closing it. SCC is filled in the vertical part of the apparatus, and excess water should be removed. To let the SCC flow into the horizontal part of the apparatus, raise the sliding gate. Start the stopwatch and time how long it takes for the SCC to reach the 200 and 400 marks. The distances "H1" and "H2" are quantified when the SCC stops flowing. Calculate the blocking ratio, "H2/H1". Within five minutes, the entire task must be completed.

#### U Box Test

SCC filing capacity is assessed using the U Box test. The device contains a vessel with a middle wall separating it into two sections and a sliding gated opening between them. A reinforcing bar with a nominal diameter of 134 mm and 50 mm between centers is installed at the gate. This results in a clear 35 mm space between bars. About 20 liters of concrete are added to the left section, and after the gate is raised, the concrete flows upward into the right section. In both sections, the concrete’s height is gauged. The performance of the entire test is completed within 5 minutes.

*4*.*2*.*2*.*2 Hardened concrete properties*. The ability of a material or structure to support loads on its surface without cracking or deflecting is known as compressive strength. When a material is subjected to external loads, the material is compressed or disintegrated from the inside. Various external factors, like relative humidity, curing process, temperature, etc., are influencing the concrete strength. Concrete compressive strength is estimated using IS: 516–2006 [92]. The size of the cube specimen cast is 150 x 150 x 150 mm, and SCC is properly tempered to prevent voids. Molds are removed after 24 hours, and the specimens are cured in water. The specimen is tested at 7 and 28 days as per IS: 516–2006 [92].

## 5. Results and discussion

### 5.1 Influence of normalisation techniques, transfer functions and k-fold method

Normalisation techniques are conventionally considered as a technique to preprocess the data set. Data preprocessing is one of the vital stages in the development of a solution, and the choice of the preprocessing technique can significantly affect the performance of algorithms in ANN [93]. Recently, the collected data have become more complex to save memory and computational cost, and the feature’s relevance is based on normalisation techniques [94]. The influence of normalisation techniques (three techniques) on the ANN-based model performance with three sensitive assessments as R^2^, MSE, and Mean Absolute Error (MAE) is reported by Nino-Adan et al., 2016 [95]. It is reported that in the complex data set without proper preprocessing, the predicted model is not performing well [96]. Pre-processed data used for modelling has a huge impact on the model, is noted by Aksu et al., 2019 [96]. The impact of adopting different transfer functions on the model performance is reported by Aidan et al., 2020 [97]. The influence of normalisation techniques and transfer function (LOGSIG and TANSIG) on the ANN model for energy transportation is reported by Reyes-Tellez et al., 2020 [98], which is analysed using two assessment techniques.

**Fig 7:**
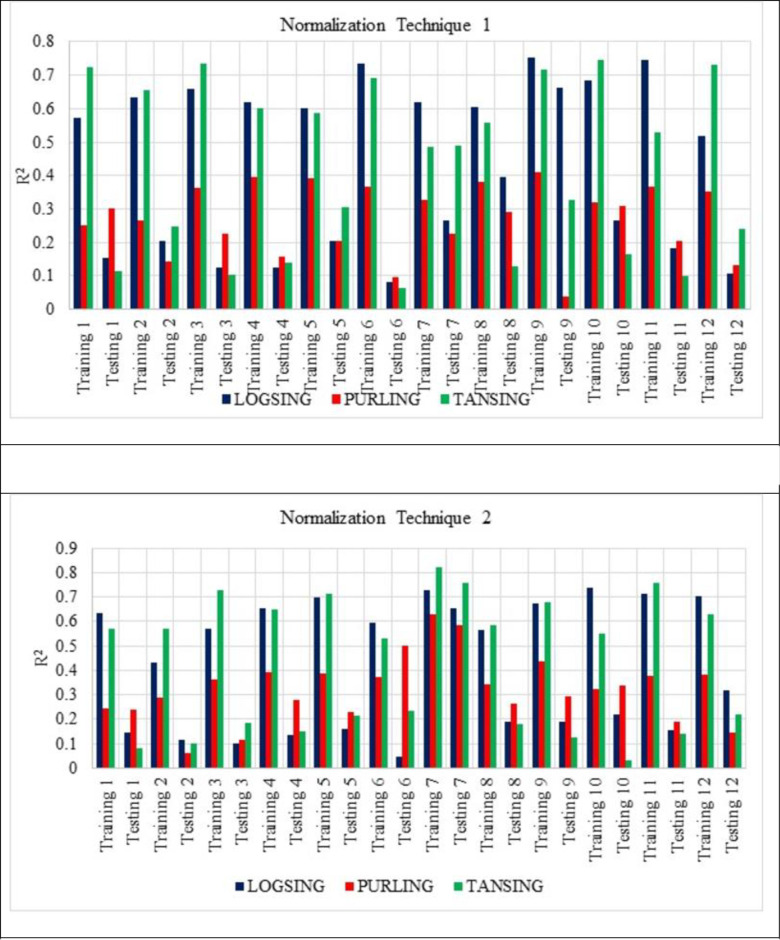
(a). Sensitive assessment based on R^2^ for Normalization Technique 1. (b). Sensitive assessment based on R2 for Normalization Technique 2.

**Fig 8:**
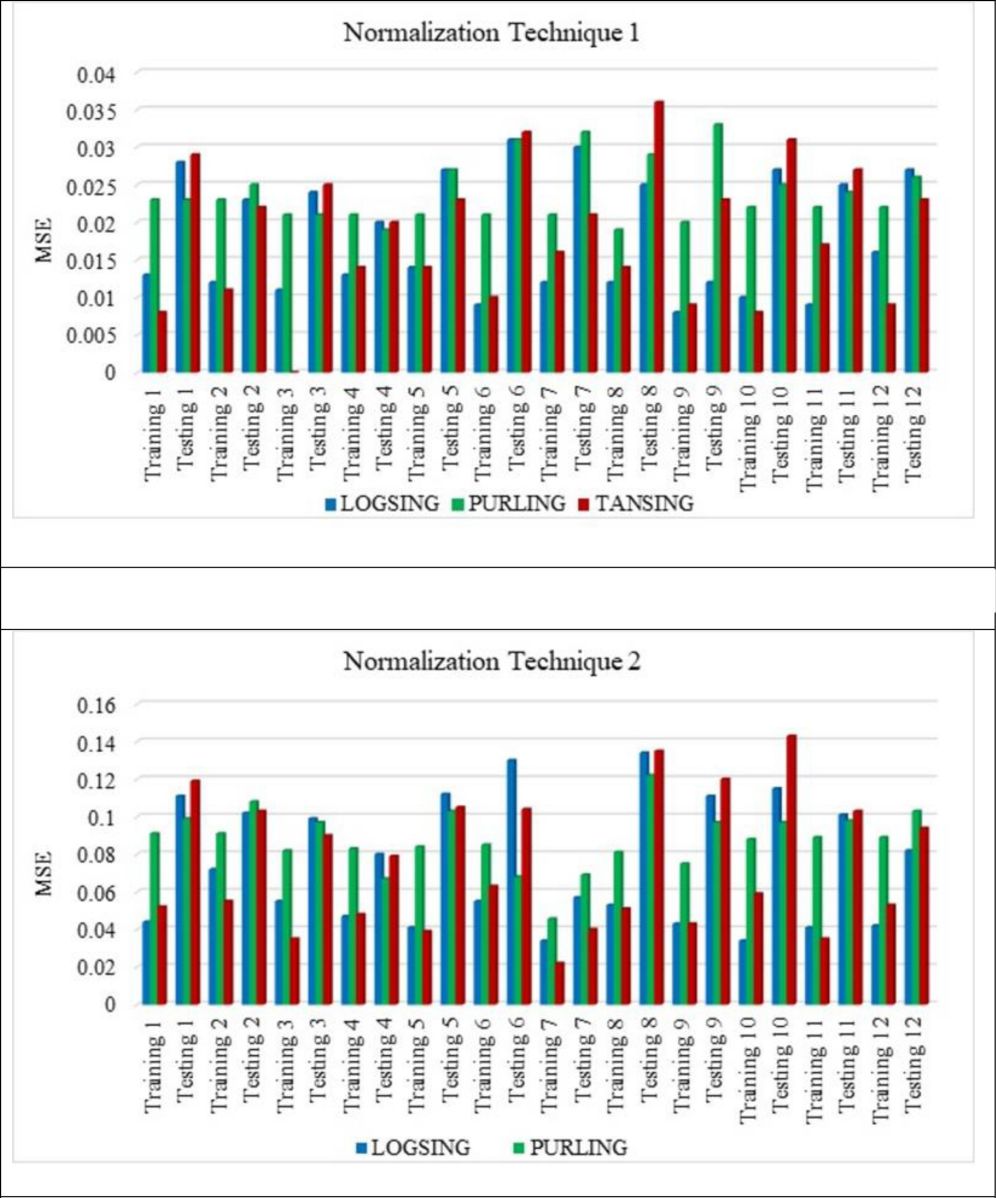
(a). Sensitive assessment based on MSE for Normalization Technqiue 1. (b). Senstive assessment based on MSE for Normalization Technique 2.

Fig 7 shows that the normalisation technique 2 shows better value than the normalisation technique 1. From Fig 7A, the highest average R^2^ value is noted for the sub-set 9 with LOGSING as average R^2^ = 0.707, PURLING as average R^2^ = 0.224 and TANSING as average R^2^ = 0.523. Fig 7B shows the highest average R^2^ value for sub-set 7 with LOGSING as average R^2^ = 0.690, PURLING as average R^2^ = 0.606 and TANSING as average R^2^ = 0.789. Compared to Fig 7A and 7B, the best sub-set was found to be sub-group 7 because it shows the highest average value in terms of training and testing observed for normalisation technique 2. From Fig 8A, the highest average MSE value is noted for the sub-set 9 with LOGSING as average MSE = 0.010, PURLING as average MSE = 0.027 and TANSING as average MSE = 0.016. From Fig 8B, the highest average MSE value is noted for the sub-set 7 with LOGSING as average MSE = 0.046, PURLING as average MSE = 0.057 and TANSING as average MSE = 0.031. Fig 8A and 8B show that the lowest MSE value is indicated for normalisation technique 2 the sub-set 7.

**Fig 9:**
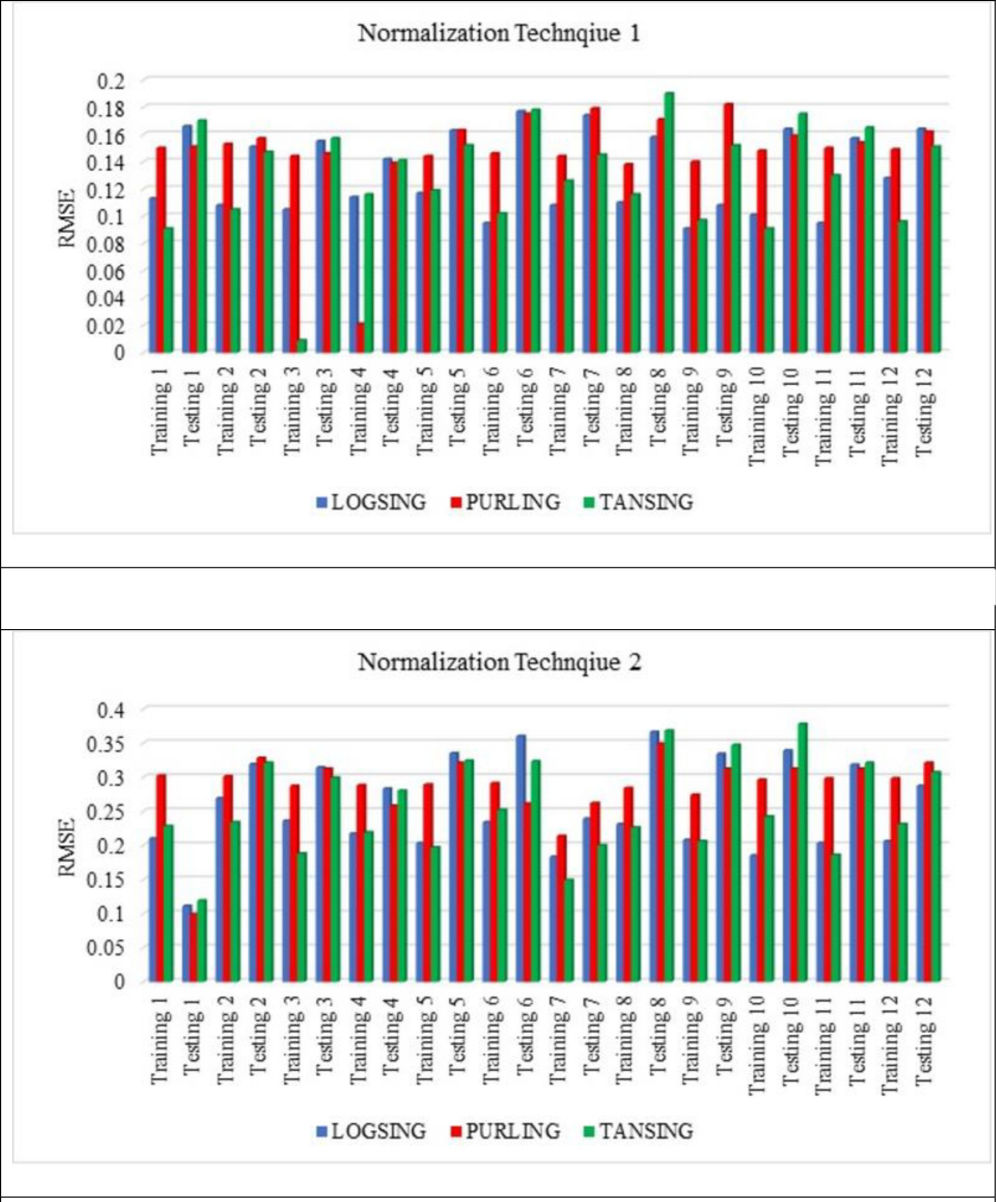
(a). Sensitive assessment based on RMSE for Normalization Technqiue 1. (b). Senstive assessment based on RMSE for Normalization Technique 2.

From Fig 9A, the highest average MSE value is noted for the sub-set 9 with LOGSING as average RMSE = 0.141, PURLING as average RMSE = 0.162 and TANSING as average RMSE = 0.136. From Fig 9(B), the highest average RMSE value is noted for the sub-set 7 with LOGSING as average RMSE = 0.271, PURLING as average RMSE = 0.293 and TANSING as average RMSE = 0.277.

From Fig 9A and 9B, it is noted the highest RMSE value is noted for the normalisation technique 2 for the sub-set 7. Even the application of five normalisation techniques on the dataset and assessment using the coefficient of determination is reported by Aksu et al., 2019 [84]. Optimisation of data is obtained by K-fold method (K = 12). In K-fold method, the given data set is divided into 10 number of subsets and this sub-sets are combined in 12 different forms to form 12 groups. In each group, 70% of data (i.e., 7 sub datasets) and 30% of data (i.e., 3 sub datasets) are used for the modelling. The number of layers used is 2 and the number of neurons is 8 for data optimisation. In most of the literature, the number of neurons is taken as number of input variables used for ANN modelling and the number of layers is kept as 2. Several hidden layers are kept as 2 because input layer and output layer. The number of layers and neurons are kept constant for optimising the normalisation technique.

In contrast, the parameters like transfer functions, normalisation technique and k-fold-cross validation are varied. From Figs 7–9 show that the higher R^2^ value is noted for the normalisation technique 2, and the lower MSE, RMSE value is noted for the normalisation technique 2. In specifically, for the transfer function, the TANSIG shows better performance in terms of R^2^, MSE and RMSE when compared to the other two transfer functions (LOGSING and PURLING). Normalisation technique is used with the transfer function as TANSIG for ANN for further investigations. K-fold cross-validation is used to predict the torsional strength of reinforced concrete beam, is proposed by Lyu et al., 2022 [99]. Similarly, three sensitive parameters are used along with K-fold cross-validation to estimate the compressive strength of concrete using machine learning techniques, is reported by Xu et al., 2021 [100].

### 5.2 Influence of number of neurons

Neural network architecture is designed based on experimental work, which will produce the best results for the specific problem. Neural network size influences the network architecture’s complexity, learning time and capability. In order to keep the size of a neural network, the learning time is also kept small resulting in optimised neural network requirements [101]. Depending upon the complexity of the problem, if the number of neurons is chosen as less, the underfitting of the model may occur. On the other hand, if the problem complexity is more, the number of selected neurons as more means results in the overfitting of the model, hence the neurons will be affecting the performance of network [102].

ANN model becomes more complex patterns and the data’s used for model and the relationship between variables also becomes complex. An increase in number of neurons leads to a struggle with the model predicted due to high dimensional spaces in the data. Such behavior is called as ‘cruse of dimensionality [103]. During the training of the data, when the number of neurons increases, the computational of the network becomes complex; such a scenario during the training of data is known as ‘computational efficiency [104]. To estimate the suitable number of neurons, it is significant to strike a balance between the model’s complexity and the data’s generalisation. In order to optimise the number of neurons, the trial-and-error method is adopted by increasing the number of neurons and monitoring both the training and validation of the data set. From **Table 9**, it is noted that a number of neurons is 16 and the minimum is 3. Whereas, the number of input parameters is denoted as Ni, number of output parameters is denoted as No and number of hidden layers is denoted as NHL. However, most of the authors found that the number of neurons is below 25. Hence, in this study, the number of neurons as 18, which is further used for this investigation.

**Table 9:** Summary of neurons in different hidden layers proposed in the literature.

Authors	Formulas	Values taken
Asteris et al., 2021a [105]	(Ni + No)	9
Asteris et al., 2021b [106]	Ni-NHL+No	6
Wang., 1994 [107]	2Ni/3	5
Masters., 1994 [108]	(Ni + No)^0.5^	3
Li et al., 1995 [109]	[(1+8Ni)^0.5^–1]/2	4
Tamura and Tateishi., 1997 [110]	Ni– 1	7
Lai and Serra., 1997 [111]	Ni	8
Nagendra., 1998 [112]	Ni + No	9
Shibata and Yuske., 2009 [113]	(Ni x No)^0.5^	3
Sheela and Deepa., 2013 [114]	(4Ni^2^ + 3) / (Ni^2^–8)	5
Ripely., 1993 [115]	(Ni + No)/2	5
Kanellopoulas and Wilkson., 1997 [116]	2Ni	16
Hunter et al., 2012 [117]	Log((Ni-1)–No)	1

**Fig 10:**
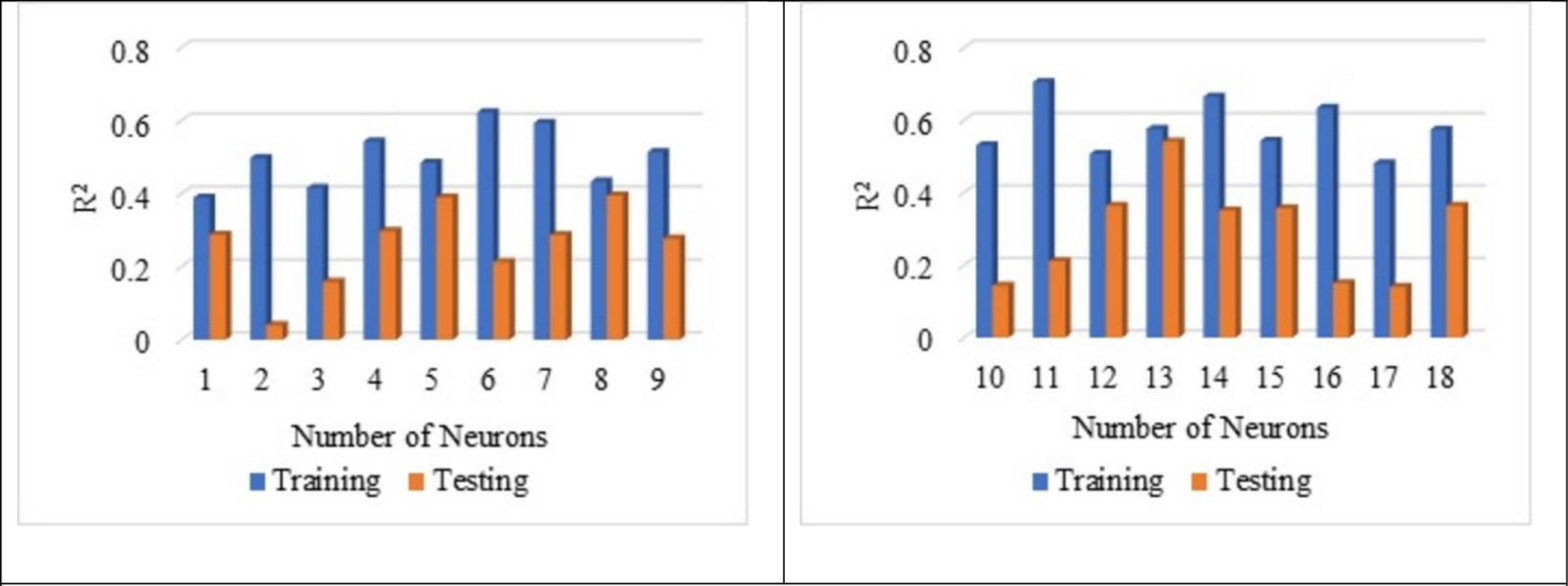
Impact of number of neurons on R^2^ value.

**Fig 11:**
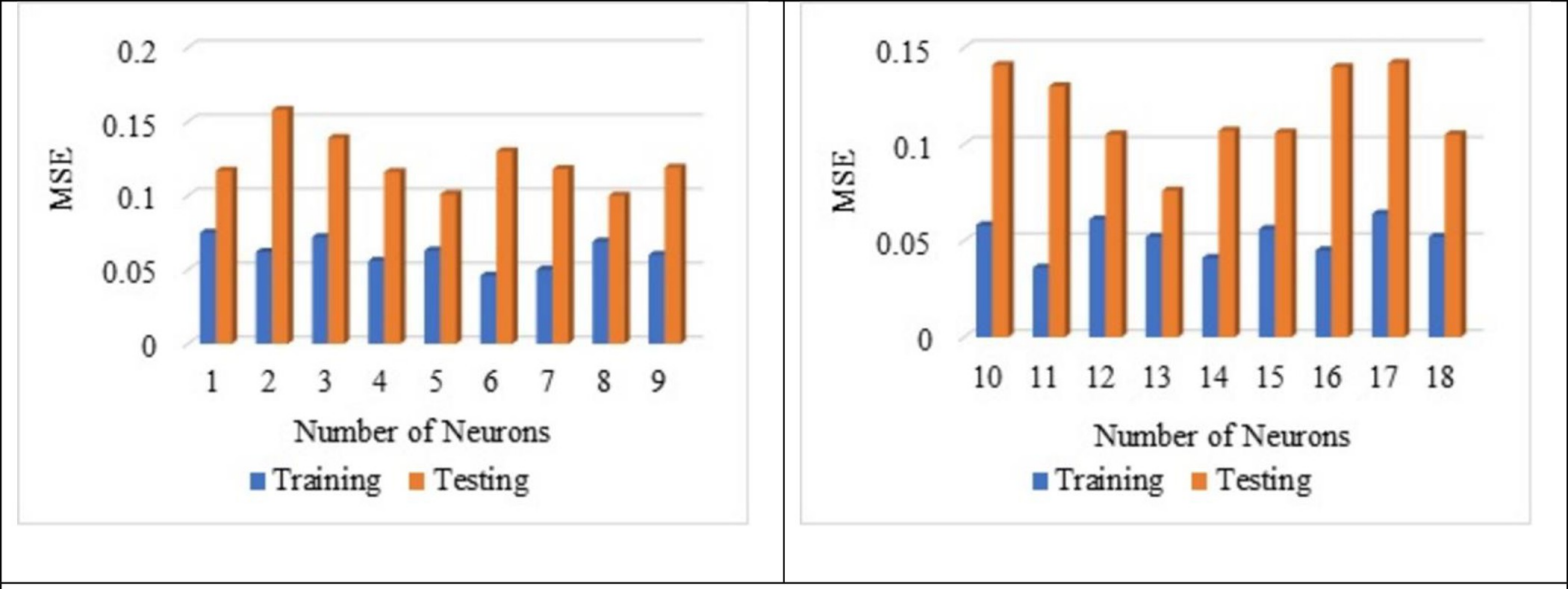
Impact of number of neurons on MSE value.

**Fig 12:**
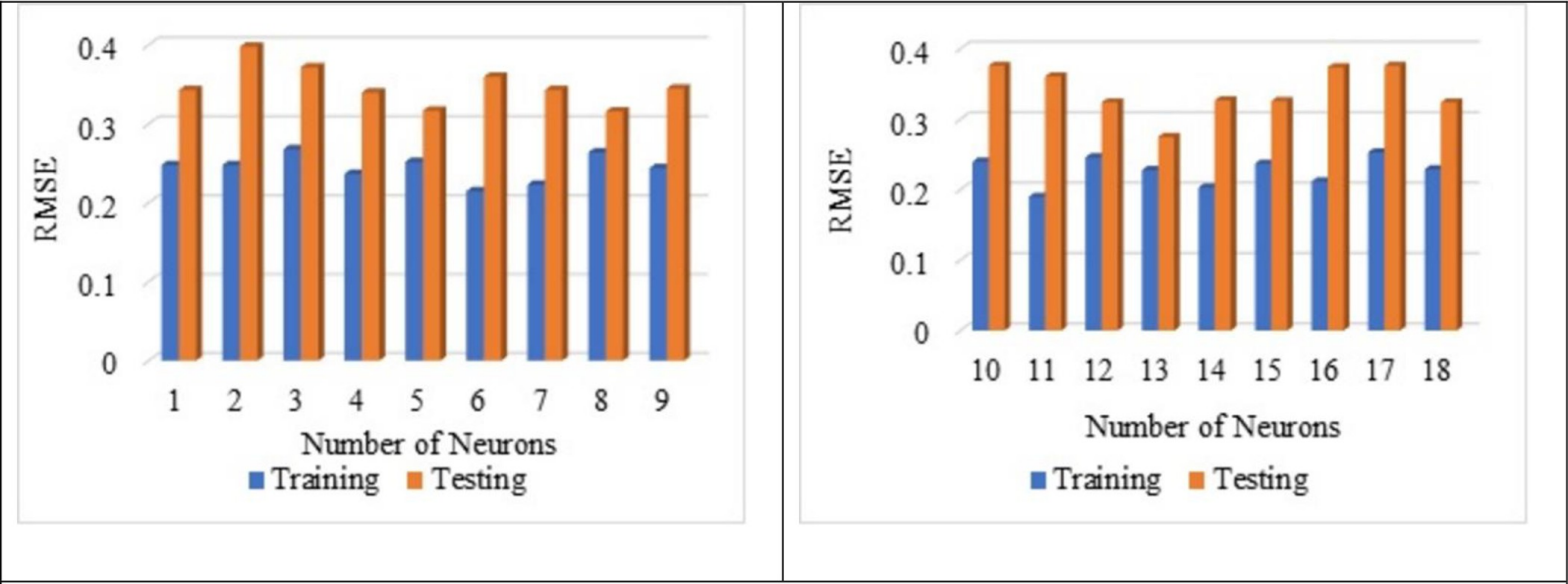
Impact of the number of neurons on RMSE value.

From Fig 10, the best average R^2^ value is noted for a number of neurons 13 as 0.576 for the training and the testing as 0.541. Hence, the average of R^2^ is noted as 0.559 for both testing and training. The influence of several neurons on the training and testing is already reported by Adil et al., 2020 [101]. From Fig 11, it is noted that the best average MSE value as 0.052 for the neuron as 13 with respect to training whereas for the testing, MSE value as 0.076. The average MSE value is reported as 0.064, which is the lowest. The best average RMSE value is noted for neuron 13 as 0.228 with respect to training whereas for the testing RMSE is noted as 0.275. The lowest average RMSE value is noted for neuron 13 as 0.252, which is the lowest among all other neurons from Fig 12.

**Fig 13:**
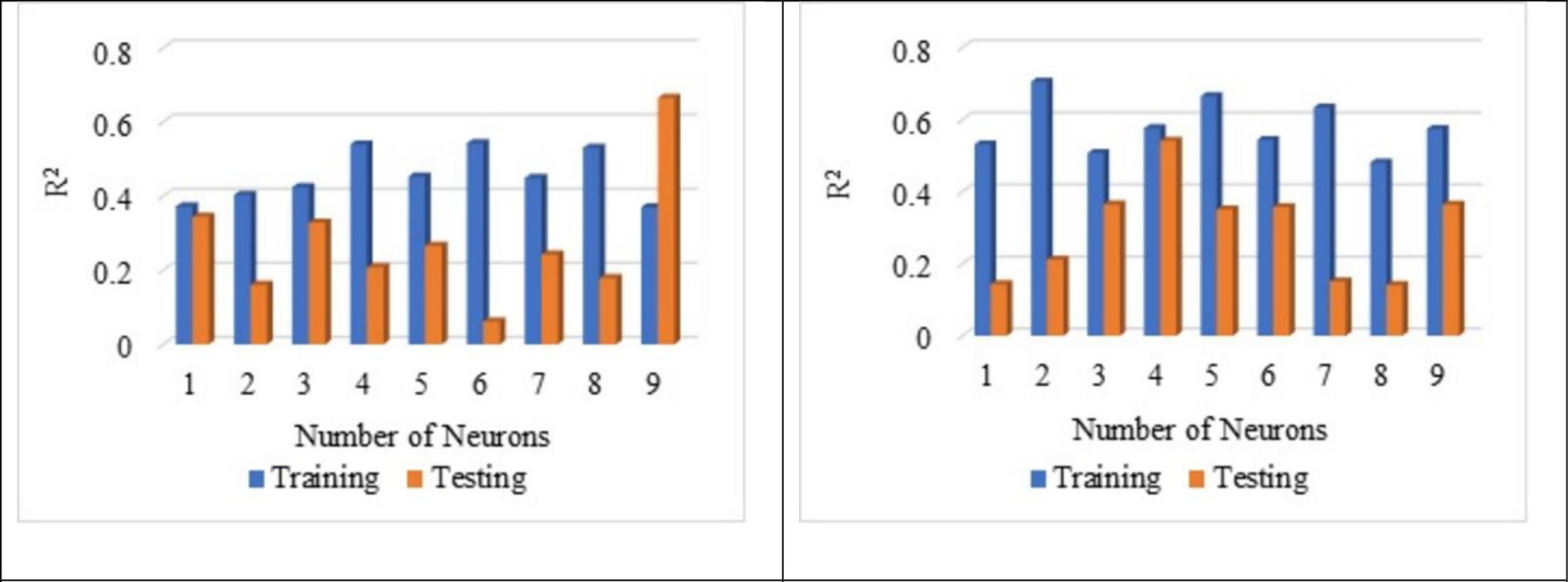
(a). Influence of number of neurons on Layer 1 based on R^2^. (b). Influence of number of neurons on Layer 2 based on R^2^.

**Fig 14:**
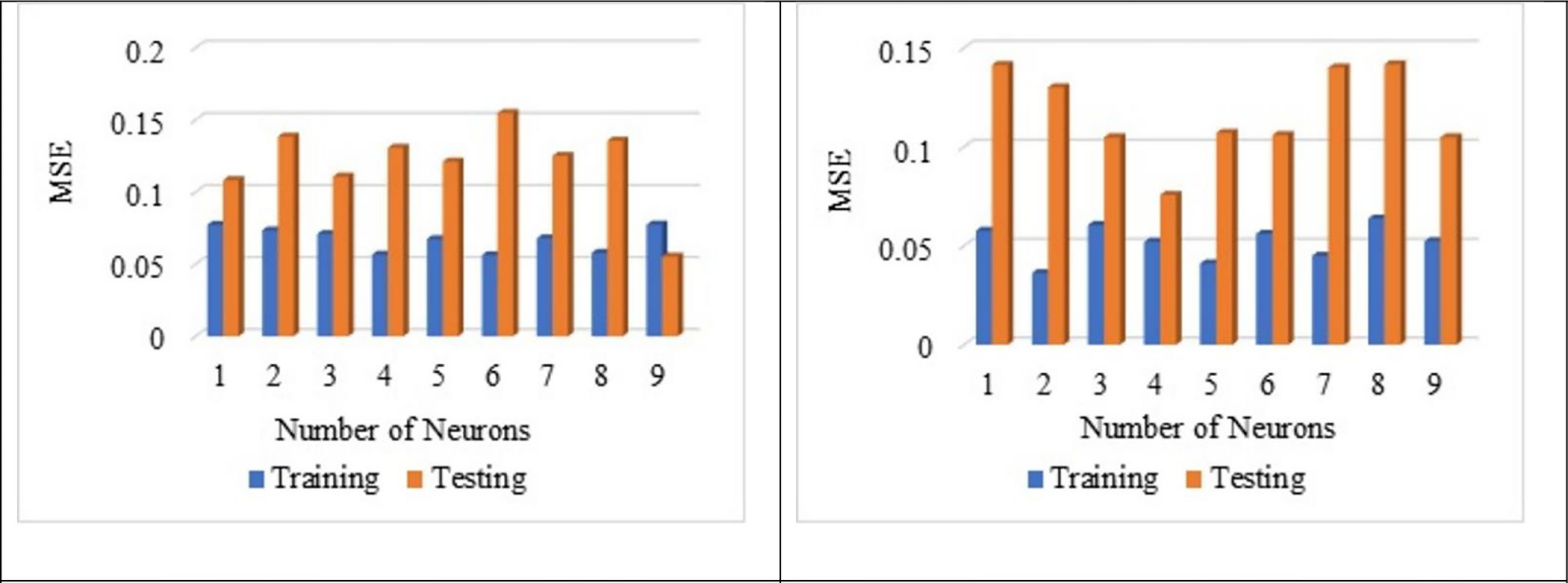
(a). Influence of number of neurons on Layer 1 based on MSE. (b). Influence of number of neurons on Layer 2 based on MSE.

**Fig 15:**
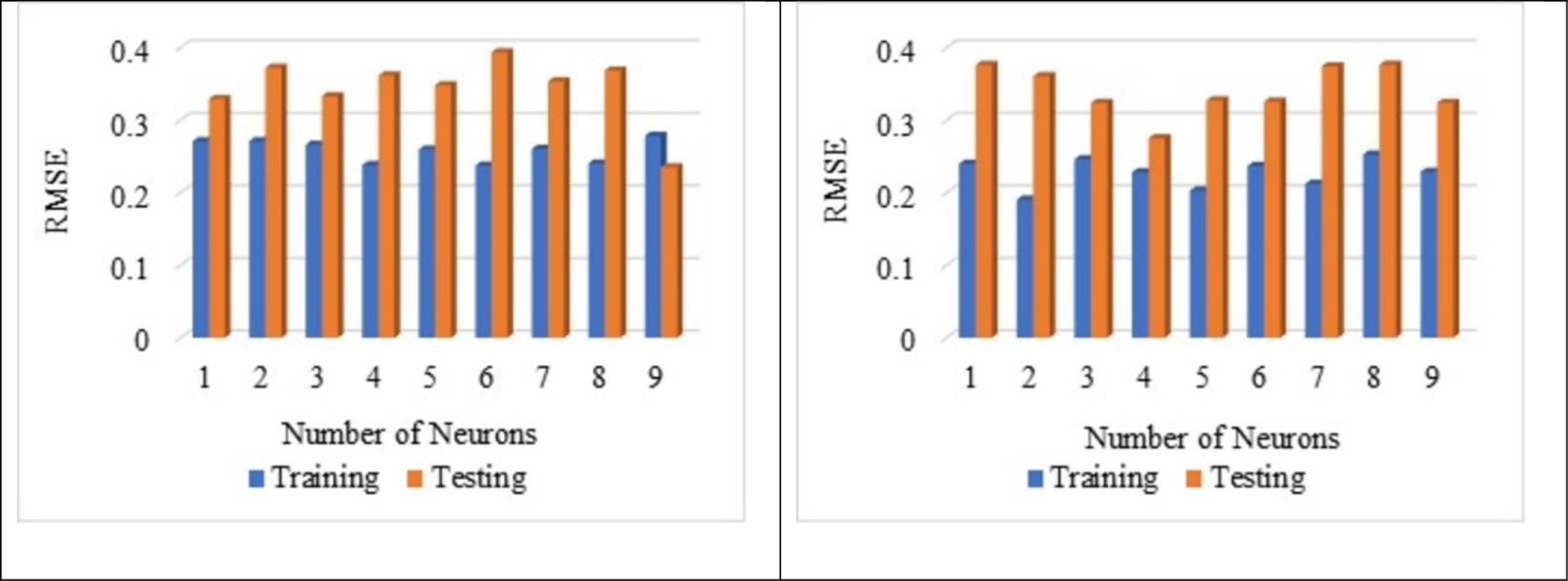
(a). Influence of number of neurons on Layer 1 based RMSE. (b). Influence of the number of neurons on Layer 2 based on RMSE.

From Figs 13A, 13B, 14A, 14B, 15A and 15B it is noted that the number of neurons 13 shows better results for the sensitive assessment R^2^, MSE and RMSE value. Lee et al., 2003 [118] reported that the increase in the number of neurons results in an increase in the number efficiency of the ANN model. However, after a certain point, an increase in the number of neurons does not impact the ANN model. Even the addition of too many neurons beyond the threshold limit leads to the overfitting of the data, and it is recommended to conduct a sensitive analysis of the models proposed [119]. Increasing the number of neurons results in overfitting and training the compressive strength prediction [120]. Variation in the number of neurons leads to change in the prediction of compressive strength, as reported by Lee., 2003 [118].

### 5.3 Influence of number of layers

Another important task in the ANN model is to optimize the number of layers. The best architecture model for ANN is selected based on the highest R^2^, least MSE and RMSE value by varying the number of layers. It is reported in the literature that the increase in the number of layers results in a decrease in the model’s efficiency [101]. However, the trial-and-error method is recommended to determine the optimised results for number of layers [101]. If the number of hidden layers provided is greater than 2, the number of neurons should be more than 80, or else the selected architecture is ineffective [101]. The best average R^2^ value is noted for number of neurons as 9 (R^2^ = 0.59 (Training), R^2^ = 0.77 (Testing) for layer 1). The best average MSE value is noted for number of neurons as 9 (MSE = 0.051 (Training), MSE = 0.039 (Testing) for Layer 1). The best average RMSE value is noted for several neurons as 9 (RMSE = 0.225 (Training), RMSE = 0.196 (Testing) for Layer 1). Similar to the effect of several neurons, the same behaviour is noted for the effect of the number of layers reported in the literature [101]. Feedforward algorithms in neural networks have their neurons arranged in the layers. All the neurons in the various layers are connected, although there is no connection between the neurons in each layer. Comparison of double and triple layers to forecast the compressive strength of confined columns using neural networks is reported by Le-Nguyen et al., 2022 [121].

### 5.4 Predicted compressive strength

**Fig 16:**
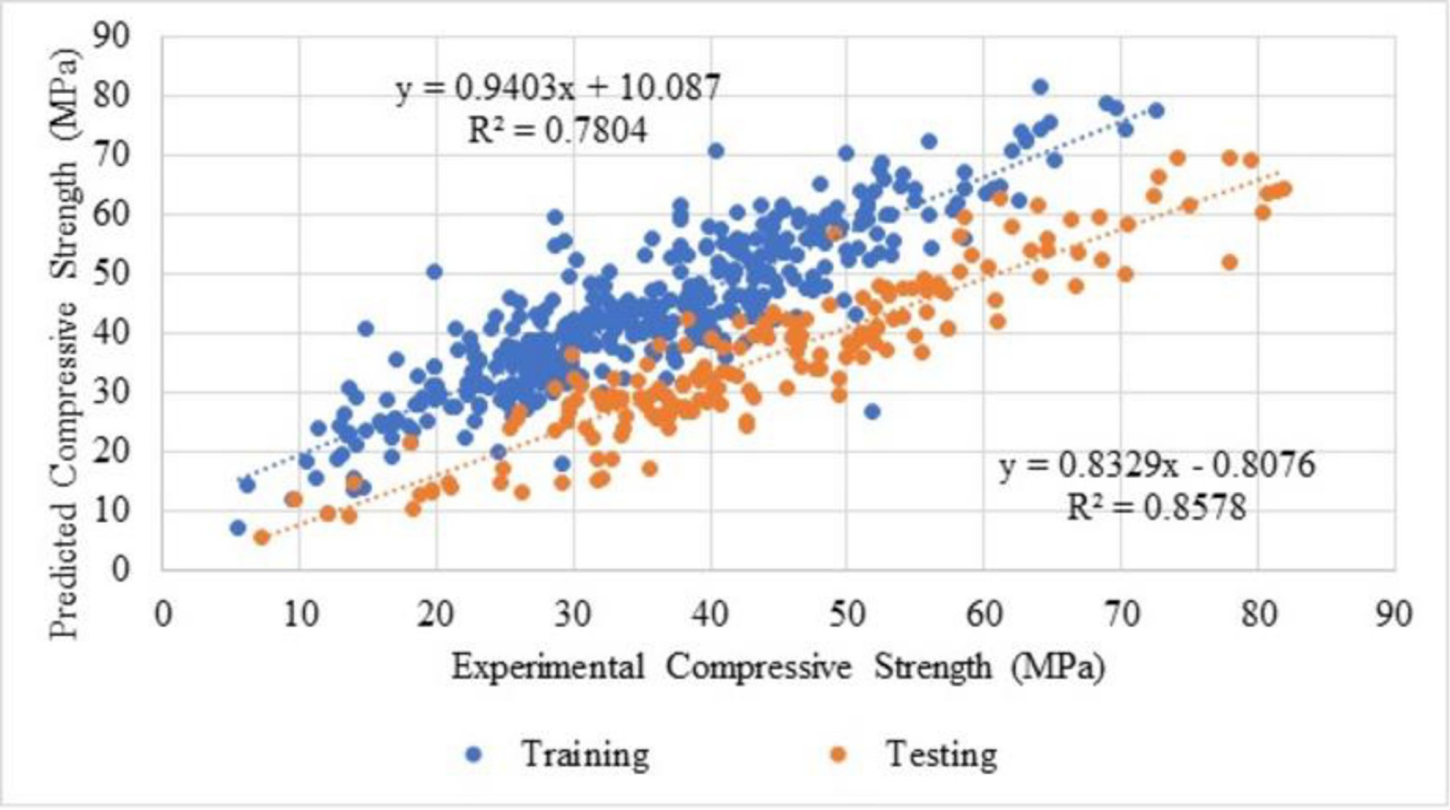
Regression analysis for training and testing dataset for predicted and experimental results.

**Fig 17:**
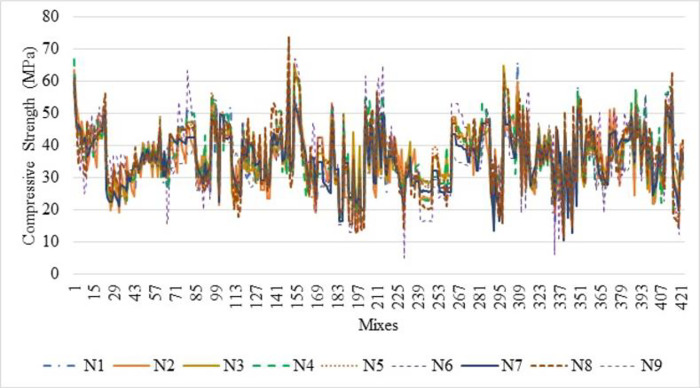
Predicted compressive strength for number of neurons 9 for layer 1.

Compressive strength prediction of recycled aggregate concrete using ANN is reported by Naderpour et al., 2018 [122]. In their studies, from 14 literatures, 139 existing data are used to forecast the compressive strength (output layer) from six input layers (water absorption, water to cement ratio, natural coarse aggregate, natural fine aggregate, water to total material ratio and the recycled coarse aggregate). In order to find the optimised model, there is variation in the neurons and layers used. Fig 16 shows that the compressive strength and the experimental model strength show better results for the training and testing models from the R^2^ value. Predicted compressive strength using various neurons from 1 to 9, concerning optimised layer as 1 is shown in Fig 17. Forecasting the compressive strength and modelled compressive strength for training using ANN with R^2^ value as 0.78 whereas the testing R^2^ is obtained as 0.86. Similar to Fig 17, the predicted compressive strength to the number of samples is reported by Moradi et al., 2021 [123], with a number of samples as 120.

### 5.5 Machine learning techniques

**Fig 18:**
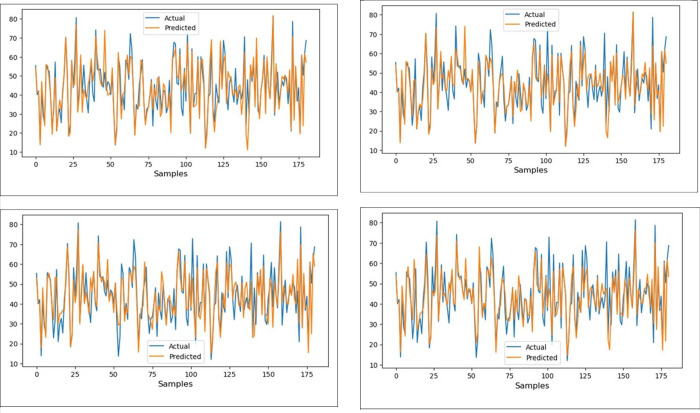
(a). Predicted and actual compressive strenght values of SCC using the empolyed XG Boost learning algorithm. (b). Predicted and actual compressive strenght values of SCC using the empolyed LightGBM algorithm. (c). Predicted and actual compressive strenght values of SCC using the empolyed extra trees algorithms. (d). Predicted and actual compressive strenght values of SCC using the empolyed Random forest algorithms.

Each machine learning algorithm was employed with its default parameters for training and testing. The results are detailed in **Table 10**. Evaluation metrics values, including MSE, RMSE, and R^2^, revealed that RF, ET, XGB and LGBM algorithms exhibited similar effectiveness, all achieving an R^2^ value exceeding 0.72, indicating a satisfactory level of predictive accuracy. Notably, XGB outperformed the others, achieving the highest R^2^ value at 0.7635 and the lowest RMSE at 6.8013. Fig 18A-18D shows a comparison between the predicted and actual compressive strength of SCCRCA using machine learning techniques ((a) XGB, (b) LGBM, (c) ET, and (d) RF). These graphs demonstrated the acceptable performance of these algorithms, because there is similarity between the predicted and actual values except some peaks where the models couldn’t predict them very well. So, these models could need more data in training phase to capture these peaks and enhance the model performance. Prediction of compressive strength of SCRAC using several ML techniques was reported by Jagadesh et al., 2023 [19].

**Table 10:** Evaluation metrics for machine learning models.

ML Model	MSE	RMSE	R^2^
**RF**	53.8046	7.3351	0.7249
**ET**	49.4027	7.0287	0.7474
**XGB**	46.2584	6.8013	0.7635
**LGBM**	50.1286	7.0802	0.7437

### 5.6 Development of model from ANN

In order to develop the equation to forecast the compressive strength of compressive strength of SCRCA, Eq (24) is proposed with the help of weight and bias to each layer from **Table 11**. Eq 24 is developed with the help of two different constants (A and B). Constant A consists of nine equations from Eqs (6) to (14), and constant B consists of another nine equations from Eqs (15) to (23).


A1=‐2.2831xCement+0.27622xwater–0.5661xFA‐0.1905xRFA‐1.0538xCA‐0.8820xRCA‐1.6613xMA‐0.5456xCHA+0.21753 (6)

A2=9.0083xCement‐39.071xWater–42.346xFA+16.1684xRFA‐4.6226xCA‐12.362xRCA‐63.546xMA‐7.432xCHA–16.597 (7)

A3=‐2.387xCement+34.7336xWater+43.0324xFA+69.6811xRFA+1.339xCA‐17.797xRCA‐13.133xMA‐4.3779xCHA–31.569 (8)

A4=0.73253xCement‐5.2991xWater‐2.1665xFA–3.6817xRFA‐0.3371xCA‐0.9753xRCA‐0.5262xMA‐2.6893xCHA–2.4523 (9)

A5=0.34836xCement+16.4305xWater‐19.193xFA–17.820xRFA‐8.1406xCA‐16.543xRCA‐18.444xMA‐0.8283xCHA+6.2896 (10)

A6=2.5528xCement‐3.2803xWater+0.20591xFA–2.0416xRFA‐2.23xCA‐2.0887xRCA‐1.901xMA‐2.1114xCHA‐1.8119 (11)

A7=‐2.9635xCement+0.88715xWater‐0.4458xFA–0.1973xRFA‐0.8156xCA‐16.543xRCA‐18.444xMA‐0.8283xCHA‐3.6754 (12)

A8=‐0.4482xCement‐2.3594xWater+5.0759xFA–3.3771xRFA‐1.9866xCA‐4.8235xRCA‐0.28xMA‐6.3203xCHA‐3.0603 (13)

A9=‐4.789xCement‐5.2472xWater‐4.5673xFA–5.2538xRFA+0.05528xCA‐2.7007xRCA‐1.3227xMA+4.1156xCHA+2.9558 (14)

$$B1=‐4.9607\;x\;f_{ck}*TANH(A1)$$ (15)

$$B2=‐0.2233\;x\;f_{ck}*TANH(A2)$$ (16)

$$B3=‐0.2387\;x\;f_{ck}*TANH(A3)$$ (17)

$$B4=0.73253\;x\;f_{ck}*TANH(A4)$$ (18)

$$B5=0.34836\;x\;f_{ck}*TANH(A5)$$ (19)

$$B6=2.5528\;x\;f_{ck}*TANH(A6)$$ (20)

$$B7=‐2.9635\;x\;f_{ck}*TANH(A7)$$ (21)

$$B8=‐0.4482\;x\;f_{ck}*TANH(A8)$$ (22)

$$B9=‐0.4787\;x\;f_{ck}*TANH(A9)$$ (23)

Predictednormalizedcompressivestrength=TANH(B1+B2+B3+B4+B5+B6+B7+B8+B9–0.5502) (24)

N_1_ –Normalised value for the input variable cement (kg)

N_2_ –Normalised value for the input variable water (kg)

N_3_ –Normalised value for the input variable fine aggregate (kg)

N_4_ –Normalised value for the input variable recycled fine aggregate (kg)

N_5_ –Normalised value for the input variable coarse aggregate (kg)

N_6_ –Normalised value for the input variable recycled coarse aggregate (kg)

N_7_ –Normalised value for the input variable mineral admixture (kg)

N_8_ –Normalised value for the input variable chemical admixture (kg)

N_9_ –Normalised value for the output variable chemical admixture (MPa)

**Table 11:** ANN weight and bias to each layer (Neuron = 9 and layer = 1).

Neuron	Weight to layer									Bias to	
	fck	Cement	Water	FA	RFA	CA	RCA	MA	CHA	Layer 1	Layer 2
1	-4.9607	-2.2831	0.27622	-0.5661	-0.1905	-1.0538	-0.882	-1.6613	0.5456	0.21753	-0.5502
2	-0.2233	9.0083	-39.071	-42.346	16.1684	-4.6226	-12.362	-63.546	-7.432	-16.597	
3	-0.2387	34.7336	43.0324	69.6811	1.339	-17.797	-13.133	-4.3779	-39.927	-31.569	
4	0.73253	-5.2991	-2.1665	-3.6817	-0.3371	-0.9753	-0.5262	-2.6893	-3.5078	-2.4523	
5	0.34836	16.4305	-19.193	-17.82	-8.1406	-16.543	-18.444	-0.8283	25.5357	6.2896	
6	2.5528	-3.2803	0.20591	-2.0416	-2.23	-2.0887	-1.901	-2.1114	0.07689	-1.8119	
7	-2.9635	0.88715	-0.4458	-0.1973	-0.8156	0.43549	0.40604	1.1427	-3.1062	-3.6754	
8	-0.4482	-2.3594	5.0759	-3.3771	-1.9866	4.8235	-0.28	-6.3203	-8.7888	-3.0603	
9	-0.4789	5.2472	-4.5673	-5.2538	0.05528	-2.7007	-3.0769	1.3227	4.1156	2.9558	

**Fig 19:**
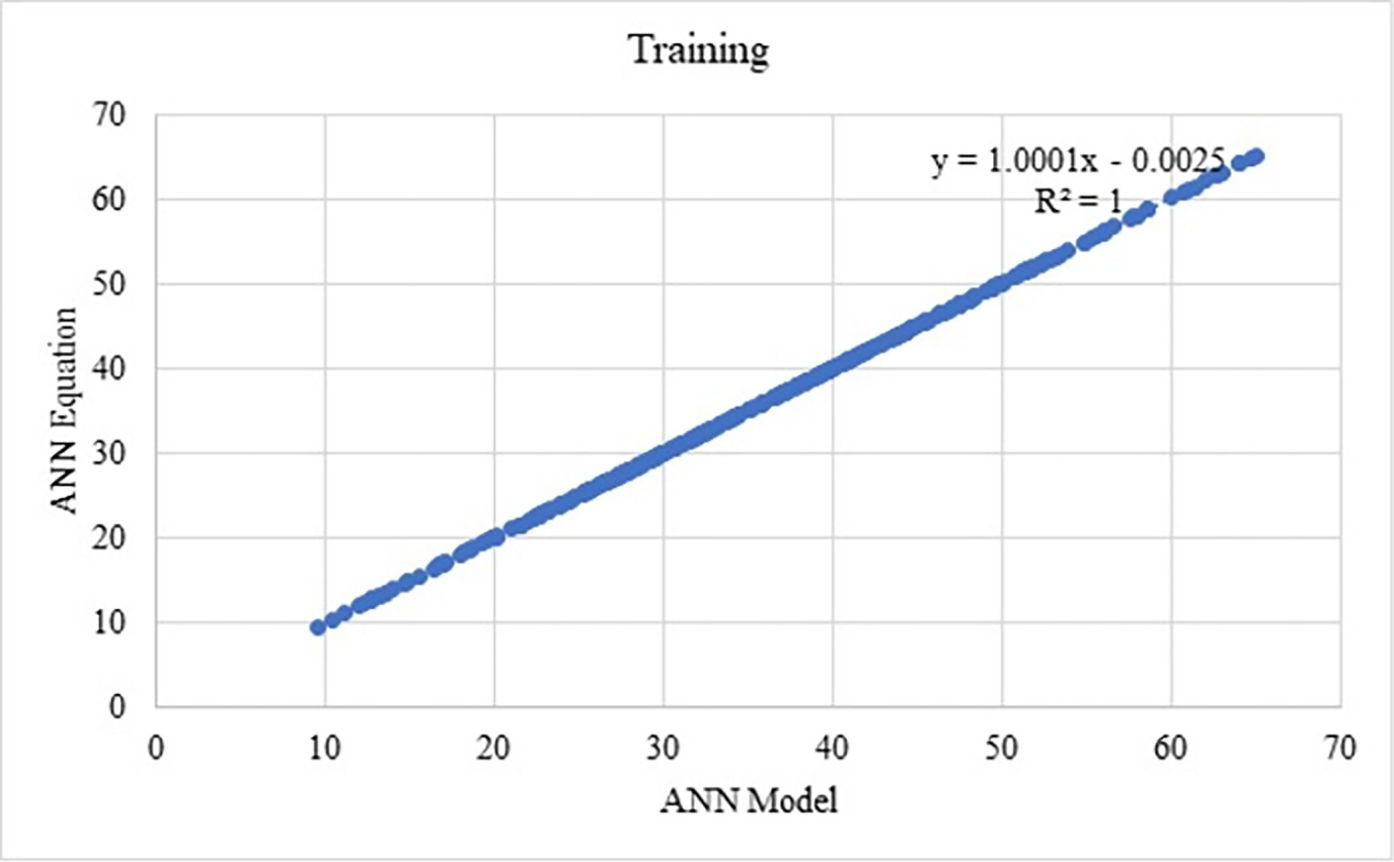
Predicted compressive strength from ANN equation and ANN models for training datasets.

**Fig 20:**
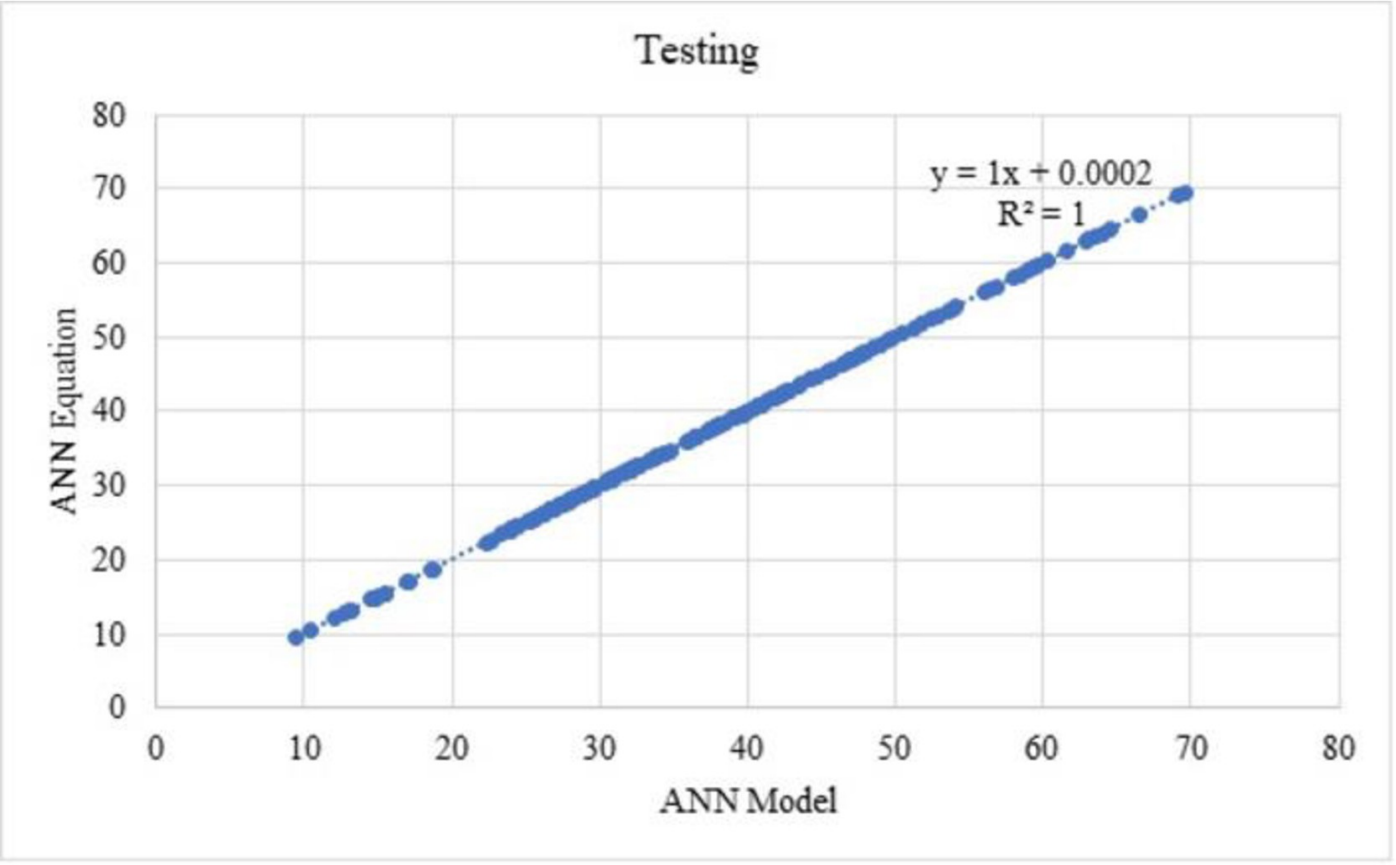
Predicted compressive strength from ANN equation and ANN models for testing datasets.

Training and testing values with respect to the model and equations from ANN are shown in Figs 19 and 20, the R^2^ value as 1. From which, it can be observed that the above equations are used to estimate each component of mix design and which can be used to determine the compressive strength of SCC. Thus, the components arrived are in the nature of normalised form, to obtain the actual form, the variables need to be denormalised.

**Fig 21:**
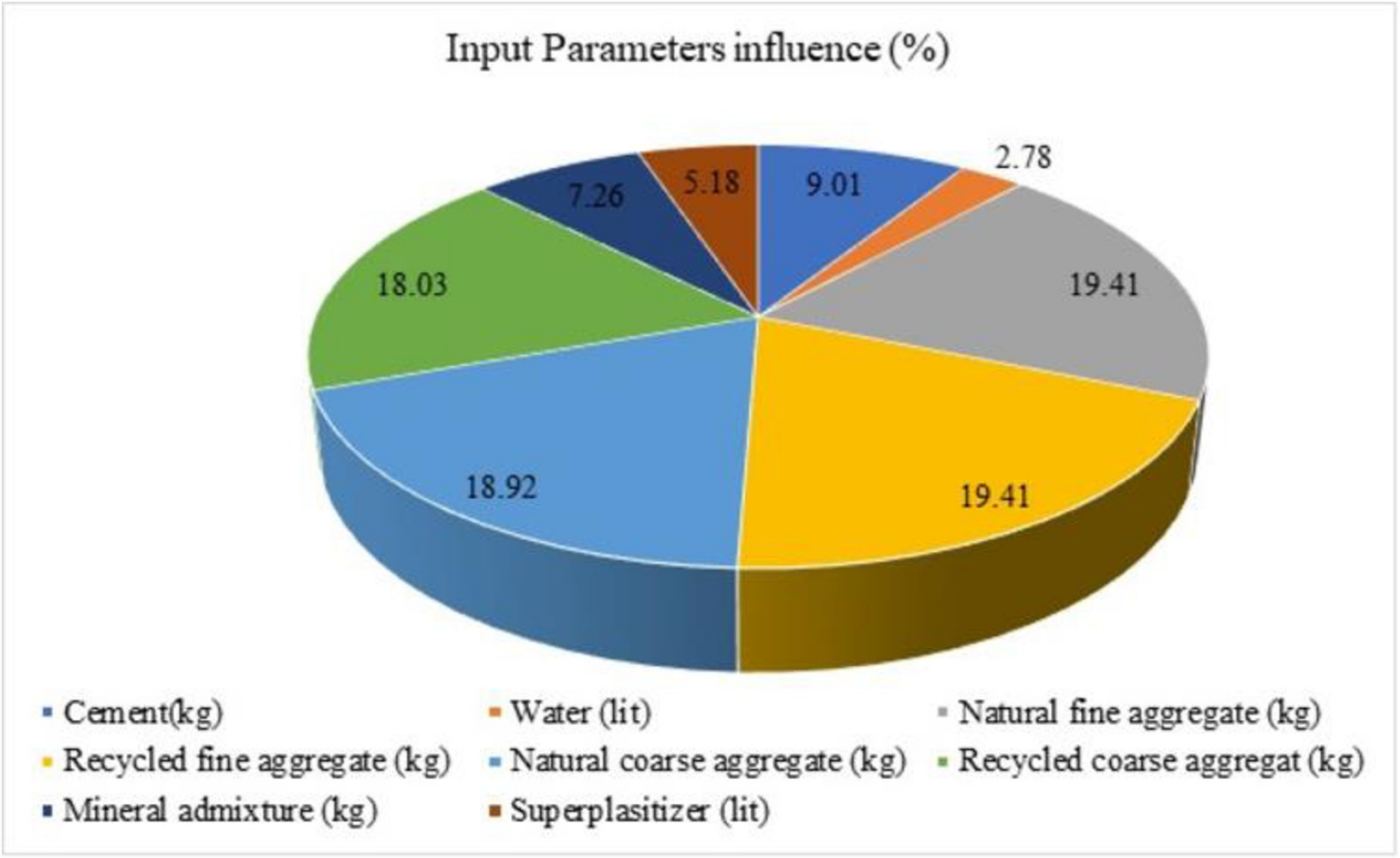
Influence of input parameters towards the prediction of compressive strength.


A proposal of the equation for the estimation of SCRCA using ANN is proposed by Dantas et al., 2013. Using the feed-forward propagation algorithm, the compressive strength is predicted using 17 input parameters and one output parameter from 1178 data (from literature). To analysis the effect of each input variable towards the forecast of the compressive strength of SCRCA, the research was supported known as the sensitivity analysis. Eq (25) and Eq (26) from previous studies [124] were used to estimate the involvement of each input variable to the outcome as the compressive strength. The contribution of each variable is depicted in Fig 21.

$$N_{i}=f_{max}(x_{i})-f_{min}(x_{i})$$ (25)

$$S_{i}=\frac{N_{i}}{\sum_{j-i}^{n}N_{j}}$$ (26)

With the help of an equation to forecast the compressive strength of SCRCA, it is necessary to develop the compositions of ingredients used to develop the mix design. By the trial-and-error method, the variables are developed, and normalization is required to check the required compressive strength. The predicted mix design from the ANN model is shown in **Table 12**.

**Table 12:** Mix design from ANN model.

Mix ID’s	Cement (kg)	Water (kg)	Fine aggregate (kg)	Recycled Fine Aggregate (kg)	Coarse Aggregate (Kg)	Recycled Coarse Aggregate (kg)	Mineral Admixture (kg)	Chemical Admixture (lit)
MT1	300	190	980	0	780	0	150	2.5
MT2	290	185	970	0	770	0	140	2.5
MT3	280	180	975	0	775	0	130	2.5

### 5.6 Concrete properties

**Fig 22:**
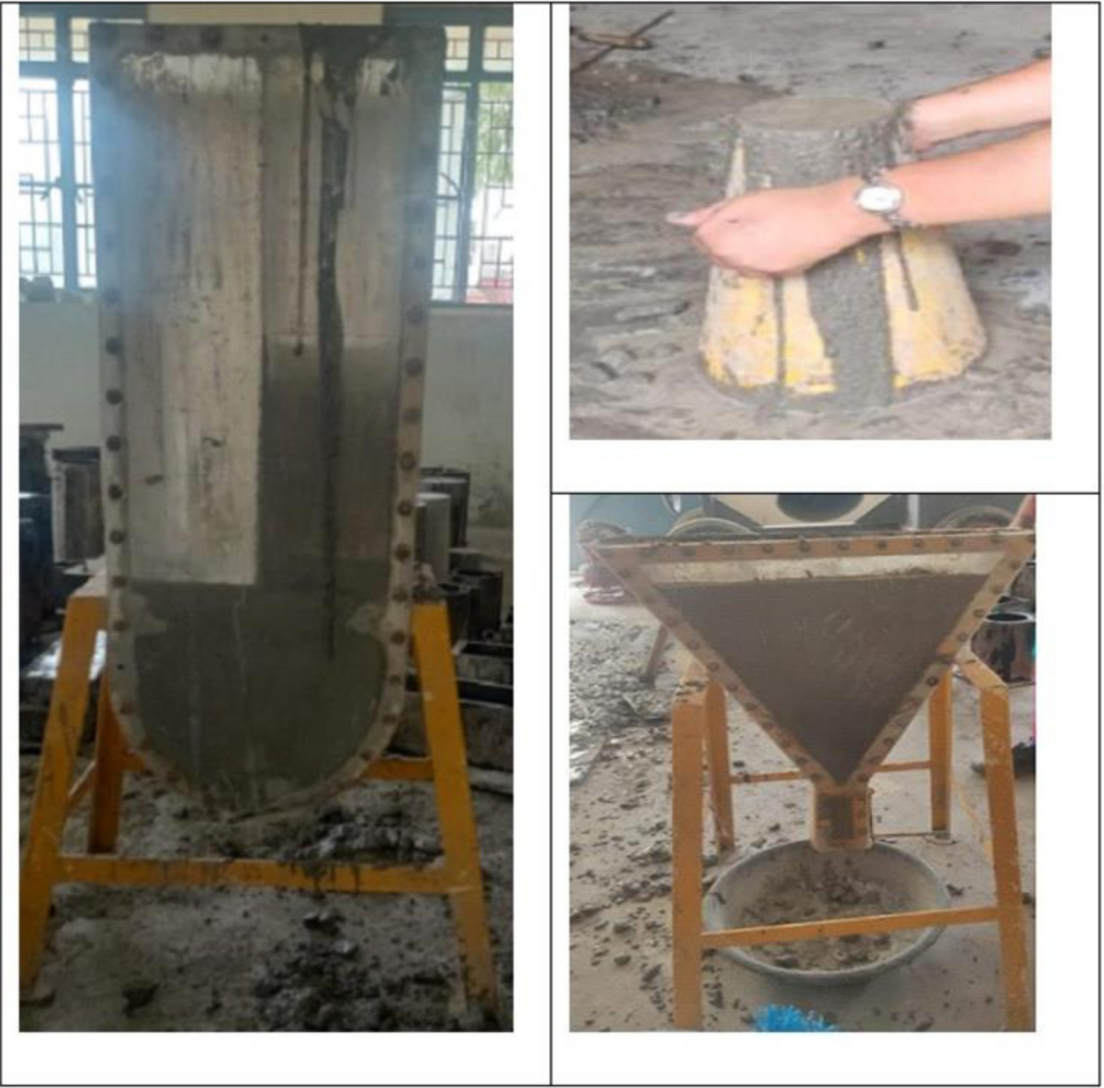
Testing of fresh concrete properties from ANN mix design model and standard mix design model.

The fresh concrete properties of mix ingredients developed earlier from the Indian standard and from ANN are evaluated, and their results are depicted in **Table 13**. The mix design developed is in the nature of control mixes in both cases. Similar procedures were adopted by authors in producing SCC, incorporating wastepaper sludge ash, pulverized fuel ash, nano silica, fly ash, limestone powder and glass powder as partial substitutes [125–127]. Three trial mixes are formed in each case, and their corresponding fresh concrete properties are found. From the results, it can be seen that the experimental and machine learning mixes show similar results. Hence, it can be recommended to use the mix design procedure from the ANN model can be used for application purposes. Experimental tests are depicted in Fig 22. From **Table 14**, it is noted that the mix design from ANN and standards shows similar results. Hence, developing the mix design from ANN for industrial applications is recommended.

**Table 13:** Fresh concrete properties of SCC.

ID No.	Slump flow (mm)	V Funnel (s)	L Box	U Box (mm)
ET1	602	10.3	0.93	2.9
ET2	615	10.9	0.92	3.1
ET3	585	10.1	0.89	2.7
MT1	605	10.4	0.93	3.0
MT2	620	11.1	0.93	3.2
MT3	595	10.2	0.90	2.8

**Table 14:** Cube compressive strength at 7 and 28 days.

ID No.	7^th^ day Compressive Strength (MPa)	28^th^ day Compressive Strength (MPa)
ET1	26.40	37.92
ET2	27.02	38.53
ET3	25.94	36.87
MT1	26.63	38.16
MT2	27.31	39.07
MT3	26.22	37.86

## 6. Conclusions

This paper is intended to demonstrate the potential use of ANN to forecast the compressive strength of SCRCA. Using 602 data samples that can be found in various published literature sources, the ANN model is built, trained, and evaluated. Eight input data, including the mass of natural fine aggregate, recycled coarse aggregate, natural coarse aggregate, water, cement, mineral admixture, and chemical admixture utilized in the mix designs, were used to create the models. Two normalizing techniques are used to visualize the data distribution. Standards are utilized as inputs to project the compressive strength on day 28. The study was also expanded to compare the 28^th^ day compressive strength using ANN with the 28^th^ day compressive strength determined from experimental testing. The findings from these studies are scheduled as follows:

- Normalization technique 2 shows a better result than the normalization technique 1. Tansing transfer function performed better than the other transfer function is reported. Among k-fold cross-validation, the best fold is noted for K = 7.

- The coefficient of determination value for predicted and actual compressive strength is 0.78 for training and for testing is 0.86.
- Mix design from ANN model with the help of weights and biases are developed. The most influential parameter for SCC is fine aggregate, and coarse aggregate is observed from mix design.
- The fresh and hardened properties of standard and ANN mix designs show similar results.
- Average compressive strength from ANN model is 39.067 MPa, whereas the compressive strength from the experimental is 38.36 MPa. This indicates that ANN can be used to develop and predict a model. The correlation coefficient value of predicted compressive strength for testing and training is 1.

## Supporting information

S1 File Supporting information is added as supplementary data. (DOCX)

S1 Graphical abstract (TIF)
