# Correction: Artificial neural network, machine learning modelling of compressive strength of recycled coarse aggregate based self-compacting concrete

**DOI:** 10.1371/journal.pone.0322947

**Published:** 2025-04-22

**Authors:** P. Jagadesh, Afzal Husain Khan, B. Shanmuga Priya, A. Asheeka, Zineb Zoubir, Hassan M. Magbool, Shamshad Alam, Omer Y. Bakather

The second author’s name is spelled incorrectly. The correct name is: Afzal Husain Khan.

The Funding statement for this paper is incorrect. The correct Funding statement is as follows: The authors extended their appreciation to the Deputyship for Research & innovation, Ministry of Education in Saudi Arabia for funding this research work though the project work number ISP23–144.

## References

[1] Jagadesh P, Khan AH, Priya BS, Asheeka A, Zoubir Z, Magbool HM, et al. Artificial neural network, machine learning modelling of compressive strength of recycled coarse aggregate based self-compacting concrete. PLoS One. 2024;19(5): e0303101. doi: 10.1371/journal.pone.0303101 38739642 PMC11090367

