# Supplementary material for: Artificial neural network, machine learning modelling of compressive strength of recycled coarse aggregate based self-compacting concrete
Source: PLoS One. 2024 May 13;19(5):e0303101. doi: 10.1371/journal.pone.0303101 (PMC11090367; doi:10.1371/journal.pone.0303101)
Supplement: S1 File — (DOCX) [file pone.0303101.s001.docx]

List of mix data from the literature

| Authors | fck (MPa) | Cement (Kg/m3) | Water (Kg/m3) | FA(Kg/m3) | RFA(Kg/m3) | CA(kg/m3) | RCA (Kg/m3) | MA(Kg/m3) | CA(Kg/m3) |
| --- | --- | --- | --- | --- | --- | --- | --- | --- | --- |
| Duan et al., 2020 | 42.91 | 430.5 | 172.2 | 660 | 0 | 780 | 0 | 184.5 | 1.53 |
| Duan et al., 2020 | 42.41 | 430.5 | 172.2 | 660 | 61.5 | 780 | 0 | 123 | 1.53 |
| Duan et al., 2020 | 40.25 | 430.5 | 172.2 | 660 | 61.5 | 585 | 195 | 123 | 1.53 |
| Duan et al., 2020 | 36.93 | 430.5 | 172.2 | 660 | 61.5 | 390 | 390 | 123 | 1.53 |
| Duan et al., 2020 | 34.85 | 430.5 | 172.2 | 660 | 61.5 | 0 | 780 | 123 | 1.53 |
| Duan et al., 2020 | 41.25 | 430.5 | 172.2 | 660 | 123 | 780 | 0 | 61.5 | 1.53 |
| Duan et al., 2020 | 40.73 | 430.5 | 172.2 | 660 | 123 | 585 | 195 | 61.5 | 1.53 |
| Duan et al., 2020 | 35.97 | 430.5 | 172.2 | 660 | 123 | 390 | 390 | 61.5 | 1.53 |
| Duan et al., 2020 | 34.43 | 430.5 | 172.2 | 660 | 123 | 0 | 780 | 61.5 | 1.53 |
| Kou and Poon., 2009 | 44.3 | 340 | 180 | 695 | 0 | 0 | 895 | 200 | 8.5 |
| Kou and Poon., 2009 | 44.5 | 340 | 180 | 521 | 153 | 0 | 895 | 200 | 8.5 |
| Kou and Poon., 2009 | 43.4 | 340 | 180 | 348 | 305 | 0 | 895 | 200 | 8.5 |
| Kou and Poon., 2009 | 41.3 | 340 | 180 | 174 | 458 | 0 | 895 | 200 | 8.5 |
| Kou and Poon., 2009 | 38.7 | 340 | 180 | 0 | 610 | 0 | 895 | 200 | 8.5 |
| Kou and Poon., 2009 | 53.7 | 340 | 180 | 662 | 0 | 0 | 850 | 270 | 8.5 |
| Kou and Poon., 2009 | 64.3 | 340 | 180 | 497 | 145 | 0 | 850 | 270 | 8.5 |
| Kou and Poon., 2009 | 62.3 | 340 | 180 | 331 | 291 | 0 | 850 | 270 | 8.5 |
| Kou and Poon., 2009 | 56.3 | 340 | 180 | 166 | 436 | 0 | 850 | 270 | 8.5 |
| Kou and Poon., 2009 | 53.2 | 340 | 180 | 0 | 581 | 0 | 850 | 270 | 8.5 |
| Kou and Poon., 2009 | 53.2 | 340 | 180 | 0 | 581 | 0 | 850 | 270 | 8.5 |
| Kou and Poon., 2009 | 59.1 | 340 | 165 | 0 | 616 | 0 | 850 | 270 | 9 |
| Kou and Poon., 2009 | 64.2 | 340 | 145 | 0 | 662 | 0 | 850 | 270 | 9.5 |
| Aslani et al., 2018 | 50.39 | 180 | 202.5 | 554.42 | 0 | 758.88 | 0 | 270 | 3 |
| Aslani et al., 2018 | 47.74 | 180 | 202.5 | 498.98 | 83.69 | 682.99 | 68.47 | 270 | 2.2 |
| Aslani et al., 2018 | 46.06 | 180 | 202.5 | 443.54 | 167.37 | 607.11 | 136.94 | 270 | 2.4 |
| Aslani et al., 2018 | 45.13 | 180 | 202.5 | 388.09 | 251.06 | 531.22 | 205.41 | 270 | 2.6 |
| Aslani et al., 2018 | 43.82 | 180 | 202.5 | 332.65 | 334.74 | 455.33 | 273.88 | 270 | 2.8 |
| Aslani et al., 2018 | 22.21 | 180 | 202.5 | 554.42 | 0 | 607.11 | 65.62 | 270 | 2.4 |
| Aslani et al., 2018 | 28.63 | 180 | 202.5 | 498.98 | 83.69 | 607.11 | 65.62 | 270 | 2.6 |
| Aslani et al., 2018 | 28.01 | 180 | 202.5 | 443.54 | 167.37 | 607.11 | 65.62 | 270 | 2.8 |
| Aslani et al., 2018 | 24.03 | 180 | 202.5 | 388.09 | 251.06 | 607.11 | 65.62 | 270 | 3 |
| Aslani et al., 2018 | 27.13 | 180 | 202.5 | 332.65 | 334.74 | 607.11 | 65.62 | 270 | 3.2 |
| Aslani et al., 2018 | 38.93 | 180 | 202.5 | 554.42 | 0 | 379.44 | 215.4 | 270 | 2 |
| Aslani et al., 2018 | 38.36 | 180 | 202.5 | 498.98 | 83.69 | 379.44 | 215.4 | 270 | 2.2 |
| Aslani et al., 2018 | 39.85 | 180 | 202.5 | 443.54 | 167.37 | 379.44 | 215.4 | 270 | 2.4 |
| Aslani et al., 2018 | 37.68 | 180 | 202.5 | 388.09 | 251.06 | 379.44 | 215.4 | 270 | 2.6 |
| Aslani et al., 2018 | 40.68 | 180 | 202.5 | 332.65 | 334.74 | 379.44 | 215.4 | 270 | 2.8 |
| Nieto et al., 2019 | 25.11 | 367 | 202 | 950 | 0 | 725 | 0 | 110 | 5.5 |
| Nieto et al., 2019 | 27.65 | 367 | 202 | 950 | 0 | 580 | 140 | 110 | 5.5 |
| Nieto et al., 2019 | 35.86 | 367 | 202 | 950 | 0 | 435 | 280 | 110 | 5.5 |
| Nieto et al., 2019 | 29.2 | 367 | 202 | 950 | 0 | 290 | 420 | 110 | 5.5 |
| Nieto et al., 2019 | 34.29 | 367 | 202 | 950 | 0 | 145 | 560 | 110 | 5.5 |
| Nieto et al., 2019 | 34.17 | 367 | 202 | 950 | 0 | 0 | 700 | 110 | 5.5 |
| Nieto et al., 2019 | 24.78 | 386 | 193 | 950 | 0 | 725 | 0 | 116 | 5.8 |
| Nieto et al., 2019 | 31.25 | 386 | 193 | 950 | 0 | 580 | 140 | 116 | 5.8 |
| Nieto et al., 2019 | 40.69 | 386 | 193 | 950 | 0 | 435 | 280 | 116 | 5.8 |
| Nieto et al., 2019 | 38.56 | 386 | 193 | 950 | 0 | 290 | 420 | 116 | 5.8 |
| Nieto et al., 2019 | 38.78 | 408 | 184 | 950 | 0 | 725 | 0 | 123 | 6.1 |
| Nieto et al., 2019 | 43.01 | 408 | 184 | 950 | 0 | 580 | 140 | 123 | 6.1 |
| Nieto et al., 2019 | 44.96 | 408 | 184 | 950 | 0 | 435 | 280 | 123 | 6.1 |
| Nieto et al., 2019 | 47.69 | 408 | 184 | 950 | 0 | 290 | 420 | 123 | 6.1 |
| Nieto et al., 2019 | 38.78 | 408 | 184 | 950 | 0 | 725 | 0 | 123 | 6.1 |
| Nieto et al., 2019 | 40.09 | 408 | 189 | 950 | 0 | 580 | 140 | 123 | 6.1 |
| Nieto et al., 2019 | 41.3 | 408 | 195 | 950 | 0 | 435 | 280 | 123 | 6.1 |
| Nieto et al., 2019 | 40.54 | 408 | 201 | 950 | 0 | 290 | 420 | 123 | 6.1 |
| Nieto et al., 2019 | 38.78 | 408 | 184 | 950 | 0 | 725 | 0 | 123 | 6.1 |
| Nieto et al., 2019 | 40.78 | 408 | 189 | 950 | 0 | 580 | 140 | 123 | 6.1 |
| Nieto et al., 2019 | 43.07 | 408 | 195 | 950 | 0 | 435 | 280 | 123 | 6.1 |
| Nieto et al., 2019 | 42.94 | 408 | 201 | 950 | 0 | 290 | 420 | 123 | 6.1 |
| Tang, W., et al., 2019 | 42.9 | 320 | 195 | 645 | 0 | 870 | 0 | 220 | 1.2 |
| Tang, W., et al., 2019 | 35.84 | 320 | 195 | 645 | 0 | 435 | 435 | 220 | 1.2 |
| Tang, W., et al., 2019 | 30.36 | 320 | 195 | 645 | 0 | 0 | 870 | 220 | 1.4 |
| Tang, W., et al., 2019 | 42.5 | 320 | 195 | 645 | 0 | 435 | 435 | 220 | 2 |
| Tang, W., et al., 2019 | 35.2 | 320 | 195 | 645 | 0 | 0 | 870 | 220 | 2 |
| Tang, W., et al., 2019 | 23.7 | 320 | 195 | 0 | 645 | 0 | 870 | 220 | 2 |
| Tang, W., et al., 2019 | 25.1 | 320 | 195 | 0 | 645 | 0 | 870 | 220 | 3 |
| Ali et al., 2012 | 46.3 | 350 | 140 | 936 | 0 | 936 | 0 | 35 | 5.25 |
| Ali et al., 2012 | 43.5 | 350 | 140 | 842.4 | 79.2 | 936 | 0 | 35 | 4.55 |
| Ali et al., 2012 | 41.5 | 350 | 140 | 748.8 | 158.4 | 936 | 0 | 35 | 4.2 |
| Ali et al., 2012 | 40.4 | 350 | 140 | 655.2 | 237.6 | 936 | 0 | 35 | 4.2 |
| Ali et al., 2012 | 38.2 | 350 | 140 | 561.6 | 316.8 | 936 | 0 | 35 | 3.85 |
| Ali et al., 2012 | 35.6 | 350 | 140 | 468 | 396 | 936 | 0 | 35 | 3.85 |
| Ali et al., 2012 | 62.2 | 400 | 160 | 890 | 0 | 890 | 0 | 40 | 7.2 |
| Ali et al., 2012 | 59.4 | 400 | 160 | 801 | 75.3 | 890 | 0 | 40 | 6.8 |
| Ali et al., 2012 | 53.2 | 400 | 160 | 712 | 150.6 | 890 | 0 | 40 | 6.8 |
| Ali et al., 2012 | 51.6 | 400 | 160 | 623 | 225.9 | 890 | 0 | 40 | 6.8 |
| Ali et al., 2012 | 48.4 | 400 | 160 | 534 | 301.2 | 890 | 0 | 40 | 6 |
| Ali et al., 2012 | 47.5 | 400 | 160 | 445 | 376.5 | 890 | 0 | 40 | 6 |
| Ali et al., 2012 | 67.7 | 450 | 180 | 846 | 0 | 846 | 0 | 45 | 8.1 |
| Ali et al., 2012 | 65.2 | 450 | 180 | 761.4 | 71.6 | 846 | 0 | 45 | 7.65 |
| Ali et al., 2012 | 61.6 | 450 | 180 | 676.8 | 148.2 | 846 | 0 | 45 | 7.65 |
| Ali et al., 2012 | 58.5 | 450 | 180 | 592.2 | 214.7 | 846 | 0 | 45 | 7.65 |
| Ali et al., 2012 | 55.8 | 450 | 180 | 507.6 | 286.3 | 846 | 0 | 45 | 6.75 |
| Ali et al., 2012 | 53.6 | 450 | 180 | 423 | 357.9 | 846 | 0 | 45 | 6.75 |
| Bidabadi et al., 2020 | 41.23 | 410 | 185 | 965 | 0 | 820 | 0 | 25 | 3.28 |
| Bidabadi et al., 2020 | 40.14 | 410 | 185 | 820.2 | 144.8 | 820 | 0 | 33 | 3.28 |
| Bidabadi et al., 2020 | 37.45 | 410 | 185 | 675.5 | 289.5 | 820 | 0 | 41.2 | 3 |
| Bidabadi et al., 2020 | 34.18 | 410 | 185 | 482.5 | 482.5 | 820 | 0 | 51.9 | 2.5 |
| Bidabadi et al., 2020 | 32.69 | 410 | 185 | 289.5 | 675.5 | 820 | 0 | 62.7 | 1.6 |
| Bidabadi et al., 2020 | 29.69 | 410 | 185 | 0 | 965 | 697 | 123 | 78.9 | 1.6 |
| Bidabadi et al., 2020 | 35.84 | 410 | 185 | 965 | 0 | 205 | 246 | 29.3 | 3.28 |
| Bidabadi et al., 2020 | 33.95 | 410 | 185 | 965 | 0 | 410 | 410 | 33.47 | 3.28 |
| Bidabadi et al., 2020 | 33.02 | 410 | 185 | 965 | 0 | 410 | 287 | 37.24 | 3 |
| Bidabadi et al., 2020 | 29.78 | 410 | 185 | 965 | 0 | 287 | 533 | 40.7 | 2.5 |
| Bidabadi et al., 2020 | 28.35 | 410 | 185 | 965 | 0 | 0 | 820 | 47 | 2.5 |
| Chakkamalayath et al., 2020 | 59.85 | 474 | 167 | 703 | 0 | 1054 | 0 | 0 | 2.9 |
| Chakkamalayath et al., 2020 | 56.72 | 475 | 167 | 721 | 0 | 1030 | 0 | 0 | 2.9 |
| Chakkamalayath et al., 2020 | 40.62 | 332.5 | 167 | 721 | 0 | 1020 | 0 | 142.5 | 2.9 |
| Chakkamalayath et al., 2020 | 52.27 | 475 | 167 | 721 | 0 | 721 | 309 | 0 | 7.5 |
| Chakkamalayath et al., 2020 | 39.14 | 332.5 | 167 | 721 | 0 | 721 | 309 | 142.5 | 7.5 |
| Chakkamalayath et al., 2020 | 43.93 | 142.5 | 167 | 721 | 0 | 721 | 309 | 190 | 7.5 |
| Grdic et al., 2010 | 49.42 | 409.6 | 178 | 676 | 0 | 429.6 | 429.6 | 260 | 4 |
| Grdic et al., 2010 | 47.25 | 409.6 | 178 | 676 | 0 | 429.6 | 429.6 | 260 | 4 |
| Grdic et al., 2010 | 45.4 | 409.6 | 186 | 676 | 0 | 429.6 | 429.6 | 260 | 4 |
| Guneyisi et al., 2014 | 53.22 | 440 | 209 | 748 | 0 | 493.5 | 211.5 | 110 | 9.35 |
| Guneyisi et al., 2014 | 55.54 | 440 | 209 | 748 | 0 | 495.9 | 212.8 | 110 | 9.35 |
| Guneyisi et al., 2014 | 57.89 | 440 | 209 | 748 | 0 | 476.4 | 204.5 | 110 | 7.7 |
| Guneyisi et al., 2014 | 60 | 440 | 209 | 748 | 0 | 493.5 | 211.5 | 110 | 9.35 |
| Guneyisi et al., 2014 | 50.15 | 440 | 209 | 748 | 0 | 444.7 | 190.8 | 110 | 9.35 |
| Kapoor et al., 2016 | 38.1 | 430 | 277 | 846 | 0 | 602 | 0 | 185 | 1.72 |
| Kapoor et al., 2016 | 36.5 | 430 | 277 | 846 | 0 | 301 | 278 | 185 | 2.15 |
| Kapoor et al., 2016 | 37.8 | 430 | 277 | 635 | 195 | 301 | 278 | 185 | 2.15 |
| Kapoor et al., 2016 | 36.9 | 430 | 277 | 423 | 386 | 301 | 278 | 185 | 2.15 |
| Kapoor et al., 2016 | 33.2 | 430 | 277 | 846 | 0 | 0 | 556 | 185 | 2.58 |
| Kapoor et al., 2016 | 35.6 | 430 | 277 | 635 | 193 | 0 | 556 | 185 | 3.01 |
| Kapoor et al., 2016 | 34 | 430 | 277 | 423 | 386 | 0 | 556 | 185 | 3.01 |
| Katar et al., 2021 | 55.9 | 425 | 191.5 | 840 | 0 | 900 | 0 | 75 | 4 |
| Katar et al., 2021 | 44.3 | 425 | 191.5 | 840 | 0 | 675 | 225 | 75 | 5 |
| Katar et al., 2021 | 42.4 | 425 | 191.5 | 840 | 0 | 450 | 450 | 75 | 1.75 |
| Katar et al., 2021 | 41.8 | 425 | 191.5 | 840 | 0 | 225 | 675 | 75 | 2 |
| Khodair & Luqman., 2017 | 54.6 | 375 | 142.5 | 880 | 0 | 865 | 0 | 0 | 5.92 |
| Khodair & Luqman., 2017 | 45.1 | 187.5 | 142.5 | 880 | 0 | 865 | 0 | 187.5 | 6.32 |
| Khodair & Luqman., 2017 | 48.2 | 187.5 | 142.5 | 880 | 0 | 865 | 0 | 187.5 | 7.17 |
| Khodair & Luqman., 2017 | 54.2 | 187.5 | 142.5 | 880 | 0 | 865 | 0 | 187.5 | 7.56 |
| Khodair & Luqman., 2017 | 49.1 | 375 | 142.5 | 880 | 0 | 648.75 | 216.25 | 0 | 6.71 |
| Khodair & Luqman., 2017 | 41 | 187.5 | 142.5 | 880 | 0 | 648.75 | 216.25 | 187.5 | 7.16 |
| Khodair & Luqman., 2017 | 45.4 | 187.5 | 142.5 | 880 | 0 | 648.75 | 216.25 | 187.5 | 7.9 |
| Khodair & Luqman., 2017 | 42.6 | 187.5 | 142.5 | 880 | 0 | 648.75 | 216.25 | 187.5 | 8.9 |
| Khodair & Luqman., 2017 | 42.9 | 375 | 142.5 | 880 | 0 | 432.5 | 432.5 | 0 | 6.77 |
| Khodair & Luqman., 2017 | 32.2 | 187.5 | 142.5 | 880 | 0 | 432.5 | 432.5 | 187.5 | 8.7 |
| Khodair & Luqman., 2017 | 38.6 | 187.5 | 142.5 | 880 | 0 | 432.5 | 432.5 | 187.5 | 8.84 |
| Khodair & Luqman., 2017 | 38 | 187.5 | 142.5 | 880 | 0 | 432.5 | 432.5 | 187.5 | 9.21 |
| Khodair & Luqman., 2017 | 37.9 | 375 | 142.5 | 880 | 0 | 216.25 | 648.75 | 0 | 7.2 |
| Khodair & Luqman., 2017 | 27.6 | 187.5 | 142.5 | 880 | 0 | 216.25 | 648.75 | 187.5 | 9.23 |
| Khodair & Luqman., 2017 | 31.1 | 187.5 | 142.5 | 880 | 0 | 216.25 | 648.75 | 187.5 | 9.32 |
| Khodair & Luqman., 2017 | 29.2 | 187.5 | 142.5 | 880 | 0 | 216.25 | 648.75 | 187.5 | 11.42 |
| Kumar et al., 2017 | 43.4 | 440 | 211.2 | 846 | 0 | 750 | 0 | 146.67 | 4.4 |
| Kumar et al., 2017 | 48 | 440 | 211.2 | 846 | 0 | 600 | 133.6 | 146.67 | 4.4 |
| Kumar et al., 2017 | 45.6 | 440 | 211.2 | 676.8 | 143.82 | 750 | 0 | 146.67 | 4.4 |
| Kumar et al., 2017 | 46.9 | 440 | 211.2 | 676.8 | 143.82 | 600 | 133.6 | 146.67 | 4.4 |
| Li et al., 2019 | 43 | 344 | 186 | 835 | 0 | 810 | 0 | 186 | 5 |
| Li et al., 2019 | 36.4 | 344 | 186 | 751 | 30 | 810 | 0 | 186 | 6 |
| Li et al., 2019 | 32.7 | 344 | 186 | 668 | 60 | 810 | 0 | 186 | 6.7 |
| Li et al., 2019 | 28.6 | 344 | 186 | 585 | 90 | 810 | 0 | 186 | 7.2 |
| Manzi et al., 2017 | 43.8 | 350 | 179 | 975 | 0 | 623 | 0 | 220 | 3.5 |
| Manzi et al., 2017 | 45.4 | 350 | 172 | 731 | 165 | 467 | 195 | 220 | 3.85 |
| Manzi et al., 2017 | 50.3 | 350 | 172 | 585 | 264 | 374 | 313 | 220 | 3.85 |
| Manzi et al., 2017 | 51.1 | 350 | 179 | 975 | 0 | 0 | 589 | 220 | 4.2 |
| Martinez-Garcia., 2020 | 46.36 | 400 | 190 | 904 | 0 | 700 | 0 | 58 | 320 |
| Martinez-Garcia., 2020 | 55.58 | 400 | 190 | 904 | 0 | 560 | 140 | 58 | 4 |
| Martinez-Garcia., 2020 | 54.7 | 400 | 190 | 904 | 0 | 350 | 350 | 58 | 4.8 |
| Martinez-Garcia., 2020 | 44.04 | 400 | 190 | 904 | 0 | 0 | 700 | 58 | 5.4 |
| Mo et al., 2020 | 74.2 | 635 | 247 | 847 | 0 | 565 | 0 | 71 | 2.286 |
| Mo et al., 2020 | 72.8 | 635 | 247 | 847 | 0 | 423.75 | 141.25 | 71 | 2.29 |
| Mo et al., 2020 | 70.9 | 635 | 247 | 847 | 0 | 282.5 | 282.5 | 71 | 2.29 |
| Mo et al., 2020 | 64.7 | 635 | 247 | 847 | 0 | 141.25 | 423.75 | 71 | 2.29 |
| Mo et al., 2020 | 63.4 | 635 | 247 | 847 | 0 | 0 | 565 | 71 | 2.29 |
| Pan et al., 2019 | 45.6 | 426 | 176 | 772 | 0 | 624 | 267 | 107 | 1.16 |
| Pan et al., 2019 | 49 | 384 | 176 | 772 | 0 | 624 | 267 | 150 | 1.16 |
| Pan et al., 2019 | 42.53 | 341 | 176 | 772 | 0 | 624 | 267 | 192 | 1.16 |
| Pan et al., 2019 | 37.22 | 298 | 176 | 772 | 0 | 624 | 267 | 235 | 1.16 |
| Pan et al., 2019 | 32.11 | 256 | 176 | 772 | 0 | 624 | 267 | 278 | 1.16 |
| Pan et al., 2019 | 28.1 | 213 | 176 | 772 | 0 | 624 | 267 | 320 | 1.16 |
| Revialla-Cuesta et al., 2020 | 60 | 300 | 165 | 1150 | 0 | 0 | 525 | 180 | 6.6 |
| Revialla-Cuesta et al., 2020 | 56 | 295 | 183 | 850 | 260 | 0 | 518 | 176 | 6.5 |
| Revialla-Cuesta et al., 2020 | 44 | 288 | 198 | 555 | 510 | 0 | 508 | 173 | 6.38 |
| Revialla-Cuesta et al., 2020 | 31 | 282 | 215 | 272 | 748 | 0 | 497 | 169 | 6.25 |
| Revialla-Cuesta et al., 2020 | 30 | 276 | 232 | 0 | 977 | 0 | 487 | 166 | 6.11 |
| Salesa et al., 2017 | 54.7 | 390 | 195 | 1025 | 0 | 0 | 700 | 75 | 5.85 |
| Salesa et al., 2017 | 59.3 | 390 | 195 | 1025 | 0 | 0 | 700 | 75 | 5.85 |
| Salesa et al., 2017 | 60.1 | 390 | 195 | 1025 | 0 | 0 | 700 | 75 | 5.85 |
| Salesa et al., 2017 | 61.7 | 390 | 195 | 1025 | 0 | 0 | 700 | 75 | 5.85 |
| Sharif et al., 2013 | 48.51 | 360 | 187.2 | 850 | 0 | 690 | 0 | 5 | 2.5 |
| Sharif et al., 2013 | 47.09 | 360 | 187.2 | 765 | 85 | 690 | 0 | 5 | 2.5 |
| Sharif et al., 2013 | 46.92 | 360 | 187.2 | 680 | 170 | 690 | 0 | 5 | 2.8 |
| Sharif et al., 2013 | 45.64 | 360 | 187.2 | 595 | 255 | 690 | 0 | 5 | 2.9 |
| Sharif et al., 2013 | 45.04 | 360 | 187.2 | 510 | 340 | 690 | 0 | 5 | 3 |
| Sharif et al., 2013 | 44.52 | 360 | 187.2 | 425 | 425 | 690 | 0 | 5 | 3.1 |
| Guo et al., 2020 | 53.45 | 520 | 182 | 785 | 0 | 867 | 0 | 0 | 4.02 |
| Guo et al., 2020 | 46.54 | 520 | 182 | 785 | 0 | 433 | 416.4 | 0 | 4.16 |
| Guo et al., 2020 | 18.04 | 260 | 182 | 785 | 0 | 433 | 416.4 | 260 | 5.72 |
| Guo et al., 2020 | 33.51 | 260 | 182 | 785 | 0 | 433 | 416.4 | 260 | 5.43 |
| Guo et al., 2020 | 40.31 | 260 | 182 | 785 | 0 | 433 | 416.4 | 260 | 6.76 |
| Guo et al., 2020 | 7.17 | 130 | 182 | 785 | 0 | 433 | 416.4 | 390 | 5.94 |
| Guo et al., 2020 | 19.66 | 130 | 182 | 785 | 0 | 433 | 416.4 | 390 | 4.68 |
| Guo et al., 2020 | 35.54 | 130 | 182 | 785 | 0 | 433 | 416.4 | 390 | 6.8 |
| Guo et al., 2020 | 43.89 | 520 | 182 | 785 | 0 | 0 | 832 | 0 | 4.72 |
| Guo et al., 2020 | 21 | 260 | 182 | 785 | 0 | 0 | 832 | 260 | 4.78 |
| Guo et al., 2020 | 38.38 | 260 | 182 | 785 | 0 | 0 | 832 | 260 | 5.67 |
| Guo et al., 2020 | 49.44 | 260 | 182 | 785 | 0 | 0 | 832 | 260 | 6.86 |
| Guo et al., 2020 | 13.64 | 130 | 182 | 785 | 0 | 0 | 832 | 390 | 7.4 |
| Guo et al., 2020 | 30.84 | 130 | 182 | 785 | 0 | 0 | 832 | 390 | 7.28 |
| Guo et al., 2020 | 42.75 | 130 | 182 | 785 | 0 | 0 | 832 | 390 | 7.57 |
| Guo et al., 2020 | 29.81 | 455 | 182 | 785 | 0 | 0 | 832 | 0 | 3.19 |
| Guo et al., 2020 | 13.89 | 113.7 | 182 | 785 | 0 | 0 | 832 | 341.23 | 4.01 |
| Guo et al., 2020 | 18.35 | 113.7 | 182 | 785 | 0 | 0 | 832 | 341.23 | 1.37 |
| Guo et al., 2020 | 26.23 | 113.7 | 182 | 785 | 0 | 0 | 832 | 341.23 | 3.19 |
| Guo et al., 2020 | 19.75 | 404 | 182 | 785 | 0 | 0 | 832 | 0 | 2.83 |
| Guo et al., 2020 | 9.63 | 101 | 182 | 785 | 0 | 0 | 832 | 303 | 3.2 |
| Guo et al., 2020 | 12.07 | 101 | 182 | 785 | 0 | 0 | 832 | 303 | 1.21 |
| Guo et al., 2020 | 18.86 | 101 | 182 | 785 | 0 | 0 | 832 | 303 | 3.63 |
| Gesoglu et al., 2015 | 77.96 | 427.5 | 171 | 765 | 0 | 859.8 | 0 | 142.5 | 6.29 |
| Gesoglu et al., 2015 | 81.4 | 370.5 | 171 | 757.5 | 0 | 851.4 | 0 | 199.5 | 7 |
| Gesoglu et al., 2015 | 66.63 | 360 | 206.4 | 773.4 | 0 | 869.3 | 0 | 120 | 3.11 |
| Gesoglu et al., 2015 | 72.47 | 312 | 206.4 | 767.2 | 0 | 862.3 | 0 | 167 | 4.55 |
| Gesoglu et al., 2015 | 68.67 | 427.5 | 171 | 765 | 0 | 0 | 749.2 | 142.5 | 4.95 |
| Gesoglu et al., 2015 | 70.39 | 370.5 | 171 | 757.5 | 0 | 0 | 741.9 | 199.5 | 6.26 |
| Gesoglu et al., 2015 | 55.38 | 360 | 206.4 | 773.4 | 0 | 0 | 757.5 | 120 | 2.55 |
| Gesoglu et al., 2015 | 63.89 | 312 | 206.4 | 767.2 | 0 | 0 | 751.3 | 168 | 4.09 |
| Gesoglu et al., 2015 | 61.97 | 427.5 | 171 | 0 | 667 | 859.8 | 0 | 142.5 | 4.51 |
| Gesoglu et al., 2015 | 64.61 | 370.5 | 171 | 0 | 660.5 | 851.4 | 0 | 199.5 | 4.2 |
| Gesoglu et al., 2015 | 48.69 | 360 | 206.4 | 0 | 674.4 | 869.3 | 0 | 120 | 1.82 |
| Gesoglu et al., 2015 | 61.04 | 312 | 206.4 | 0 | 668.9 | 862.3 | 0 | 168 | 2.7 |
| Gesoglu et al., 2015 | 55.76 | 427.5 | 171 | 0 | 667 | 0 | 749.2 | 142.5 | 3.44 |
| Gesoglu et al., 2015 | 57.41 | 370.5 | 171 | 0 | 660.5 | 0 | 741.9 | 199.5 | 3.79 |
| Gesoglu et al., 2015 | 46.04 | 360 | 206.4 | 0 | 674.4 | 0 | 757.5 | 120 | 1.78 |
| Gesoglu et al., 2015 | 52.92 | 312 | 206.4 | 0 | 668.9 | 0 | 751.3 | 168 | 2.62 |
| Behera et al., 2019 | 60.76 | 313.54 | 174.06 | 927.75 | 0 | 749.7 | 0 | 209.03 | 5.23 |
| Behera et al., 2019 | 55.76 | 313.54 | 174.065 | 463.87 | 364.85 | 749.7 | 0 | 209.03 | 5.23 |
| Behera et al., 2019 | 44.54 | 313.54 | 174.06 | 0 | 729.69 | 749.7 | 0 | 209.03 | 5.23 |
| Behera et al., 2019 | 44.54 | 315.11 | 174.06 | 927.75 | 0 | 749.7 | 0 | 210.08 | 3.15 |
| Behera et al., 2019 | 38.41 | 315.11 | 174.065 | 0 | 729.75 | 749.7 | 0 | 210.08 | 4.2 |
| Bahrami et al., 2020 | 46 | 400 | 150 | 1200 | 0 | 530 | 0 | 100 | 3.5 |
| Bahrami et al., 2020 | 38.99 | 400 | 167 | 1200 | 0 | 530 | 0 | 100 | 3.8 |
| Bahrami et al., 2020 | 38.99 | 400 | 167 | 1200 | 0 | 530 | 0 | 100 | 3.8 |
| Bahrami et al., 2020 | 36.7 | 400 | 167 | 900 | 300 | 530 | 0 | 100 | 3.8 |
| Bahrami et al., 2020 | 32.8 | 400 | 167 | 600 | 600 | 530 | 0 | 100 | 3.8 |
| Bahrami et al., 2020 | 29.6 | 400 | 167 | 300 | 900 | 530 | 0 | 100 | 3.8 |
| Bahrami et al., 2020 | 24.8 | 400 | 167 | 0 | 1200 | 530 | 0 | 100 | 3.8 |
| Bahrami et al., 2020 | 37 | 400 | 167 | 1200 | 0 | 397.5 | 132.5 | 100 | 3.8 |
| Bahrami et al., 2020 | 33.51 | 400 | 167 | 1200 | 0 | 265 | 265 | 100 | 3.8 |
| Bahrami et al., 2020 | 31.36 | 400 | 167 | 1200 | 0 | 132.5 | 397.5 | 100 | 3.8 |
| Bahrami et al., 2020 | 25.83 | 400 | 167 | 1200 | 0 | 0 | 530 | 100 | 3.8 |
| Bahrami et al., 2020 | 38 | 380 | 167 | 900 | 300 | 530 | 0 | 100 | 3.8 |
| Bahrami et al., 2020 | 39.6 | 370 | 167 | 900 | 300 | 530 | 0 | 100 | 3.8 |
| Bahrami et al., 2020 | 42.1 | 360 | 167 | 900 | 300 | 530 | 0 | 100 | 3.8 |
| Bahrami et al., 2020 | 38.7 | 380 | 167 | 1200 | 0 | 397.5 | 132.5 | 100 | 3.8 |
| Bahrami et al., 2020 | 40.3 | 370 | 167 | 1200 | 0 | 397.5 | 132.5 | 100 | 3.8 |
| Bahrami et al., 2020 | 42 | 360 | 167 | 1200 | 0 | 397.5 | 132.5 | 100 | 3.8 |
| Aslani et al., 2019 | 50.4 | 160 | 180 | 323 | 321.5 | 667 | 0 | 240.5 | 4.44 |
| Aslani et al., 2019 | 24.5 | 160 | 180 | 329 | 329 | 667 | 0 | 240.5 | 3.7 |
| Aslani et al., 2019 | 25.0 | 160 | 180 | 319 | 320 | 667 | 0 | 240.5 | 3.7 |
| Aslani et al., 2019 | 22.3 | 160 | 180 | 325 | 319 | 667 | 0 | 240.5 | 2.96 |
| Aslani et al., 2019 | 25.3 | 160 | 180 | 320 | 328 | 667 | 0 | 240.5 | 2.96 |
| Aslani et al., 2019 | 24.1 | 160 | 180 | 317 | 325 | 667 | 0 | 240.5 | 2.96 |
| Aslani et al., 2019 | 23.4 | 160 | 180 | 330 | 332 | 667 | 0 | 240.5 | 2.96 |
| Aslani et al., 2019 | 19.0 | 160 | 180 | 325 | 320 | 667 | 0 | 240.5 | 2.96 |
| Aslani et al., 2019 | 25.7 | 160 | 180 | 322 | 326 | 667 | 0 | 240.5 | 2.96 |
| Mattar et al., 2019 | 34.6 | 375 | 187.5 | 795 | 0 | 970 | 0 | 0 | 1.5 |
| Mattar et al., 2019 | 33.7 | 375 | 187.5 | 795 | 0 | 970 | 0 | 0 | 1.5 |
| Mattar et al., 2019 | 36.2 | 375 | 187.5 | 795 | 0 | 970 | 0 | 0 | 1.5 |
| Mattar et al., 2019 | 32.0 | 375 | 187.5 | 795 | 0 | 970 | 0 | 0 | 1.5 |
| Mattar et al., 2019 | 35.0 | 375 | 187.5 | 795 | 0 | 700 | 235 | 0 | 1.5 |
| Mattar et al., 2019 | 34.1 | 375 | 187.5 | 795 | 0 | 460 | 460 | 0 | 1.5 |
| Mattar et al., 2019 | 34.7 | 375 | 187.5 | 795 | 0 | 460 | 460 | 0 | 1.5 |
| Mattar et al., 2019 | 31.8 | 375 | 187.5 | 795 | 0 | 460 | 460 | 0 | 1.5 |
| Mattar et al., 2019 | 31.6 | 375 | 187.5 | 795 | 0 | 220 | 670 | 0 | 1.5 |
| Mattar et al., 2019 | 31.3 | 375 | 187.5 | 795 | 0 | 0 | 670 | 0 | 1.5 |
| Mattar et al., 2019 | 30.7 | 375 | 187.5 | 795 | 0 | 460 | 460 | 0 | 1.5 |
| Mattar et al., 2019 | 32.0 | 375 | 187.5 | 795 | 0 | 460 | 460 | 0 | 1.5 |
| Mattar et al., 2019 | 28.6 | 375 | 187.5 | 795 | 0 | 460 | 460 | 0 | 1.5 |
| Mattar et al., 2019 | 59.3 | 450 | 171 | 780 | 0 | 955 | 0 | 0 | 4.5 |
| Mattar et al., 2019 | 62.0 | 450 | 171 | 780 | 0 | 955 | 0 | 0 | 4.5 |
| Mattar et al., 2019 | 61.1 | 450 | 171 | 780 | 0 | 955 | 0 | 0 | 4.5 |
| Mattar et al., 2019 | 60.1 | 450 | 171 | 780 | 0 | 715 | 240 | 0 | 4.5 |
| Mattar et al., 2019 | 58.8 | 450 | 171 | 780 | 0 | 715 | 240 | 0 | 4.5 |
| Mattar et al., 2019 | 57.0 | 450 | 171 | 780 | 0 | 460 | 460 | 0 | 4.5 |
| Mattar et al., 2019 | 58.6 | 450 | 171 | 780 | 0 | 460 | 460 | 0 | 4.5 |
| Mattar et al., 2019 | 56.0 | 450 | 171 | 780 | 0 | 460 | 460 | 0 | 4.5 |
| Mattar et al., 2019 | 57.2 | 450 | 171 | 780 | 0 | 220 | 670 | 0 | 4.5 |
| Mattar et al., 2019 | 58.1 | 450 | 171 | 780 | 0 | 220 | 670 | 0 | 4.5 |
| Mattar et al., 2019 | 55.3 | 450 | 171 | 780 | 0 | 220 | 670 | 0 | 4.5 |
| Mattar et al., 2019 | 55.4 | 450 | 171 | 780 | 0 | 0 | 870 | 0 | 4.5 |
| Mattar et al., 2019 | 54.0 | 450 | 171 | 780 | 0 | 0 | 870 | 0 | 4.5 |
| Boudali et al., 2016 | 37.9 | 350 | 176 | 887 | 0 | 791 | 0 | 140 | 7 |
| Boudali et al., 2016 | 40.0 | 350 | 176 | 887 | 0 | 791 | 0 | 140 | 8.75 |
| Boudali et al., 2016 | 41.3 | 350 | 176 | 887 | 0 | 0 | 791 | 140 | 10.5 |
| Boudali et al., 2016 | 42.0 | 350 | 176 | 887 | 0 | 0 | 791 | 140 | 12.25 |
| Boudali et al., 2016 | 39.9 | 420 | 210 | 1170 | 0 | 292 | 0 | 200 | 7 |
| Boudali et al., 2016 | 39.0 | 420 | 210 | 1170 | 0 | 292 | 0 | 200 | 8.75 |
| Boudali et al., 2016 | 42.0 | 420 | 210 | 1170 | 0 | 0 | 292 | 200 | 10.5 |
| Boudali et al., 2016 | 47.1 | 420 | 210 | 1170 | 0 | 0 | 292 | 200 | 12.25 |
| Ge et al., 2021 | 65.8 | 321 | 149.5 | 806 | 0 | 854 | 0 | 214 | 4.28 |
| Ge et al., 2021 | 53.3 | 321 | 149.5 | 605 | 160 | 854 | 0 | 214 | 4.28 |
| Ge et al., 2021 | 61.5 | 321 | 149.5 | 605 | 160 | 854 | 0 | 214 | 4.28 |
| Ge et al., 2021 | 60.5 | 321 | 149.5 | 403 | 321 | 854 | 0 | 214 | 4.28 |
| Ge et al., 2021 | 54.8 | 321 | 149.5 | 202 | 482 | 854 | 0 | 214 | 4.28 |
| Ge et al., 2021 | 50.8 | 321 | 149.5 | 0 | 642 | 854 | 0 | 214 | 4.28 |
| Revilla-Cuesta et al., 2022 | 44.9 | 300 | 185 | 1100 | 0 | 530 | 0 | 165 | 4.5 |
| Revilla-Cuesta et al., 2022 | 40.7 | 300 | 210 | 550 | 505 | 530 | 0 | 165 | 4.5 |
| Revilla-Cuesta et al., 2022 | 28.1 | 300 | 235 | 0 | 1010 | 530 | 0 | 165 | 4.5 |
| Revilla-Cuesta et al., 2022 | 45.6 | 300 | 185 | 940 | 0 | 530 | 0 | 335 | 4.5 |
| Revilla-Cuesta et al., 2022 | 42.2 | 300 | 210 | 475 | 435 | 530 | 0 | 335 | 4.5 |
| Revilla-Cuesta et al., 2022 | 28.5 | 300 | 235 | 865 | 0 | 530 | 0 | 335 | 4.5 |
| Revilla-Cuesta et al., 2022 | 35.9 | 300 | 200 | 0 | 940 | 530 | 0 | 305 | 4.5 |
| Revilla-Cuesta et al., 2022 | 28.9 | 300 | 220 | 435 | 475 | 530 | 0 | 305 | 4.5 |
| Revilla-Cuesta et al., 2022 | 15.6 | 300 | 245 | 865 | 0 | 530 | 0 | 305 | 4.5 |
| Garcia-Troncoso et al., 2021 | 73.8 | 635 | 247 | 847 | 0 | 565 | 0 | 71 | 0 |
| Garcia-Troncoso et al., 2021 | 64.4 | 635 | 247 | 847 | 0 | 0 | 565 | 71 | 0 |
| Garcia-Troncoso et al., 2021 | 56.0 | 635 | 247 | 847 | 0 | 0 | 565 | 71 | 0 |
| Garcia-Troncoso et al., 2021 | 67.4 | 635 | 247 | 847 | 0 | 0 | 565 | 71 | 0 |
| Pereira-de-Oliveira et al., 2013 | 54.2 | 284.9 | 159.3 | 730.7 | 0 | 807.5 | 0 | 370.2 | 3.12 |
| Pereira-de-Oliveira et al., 2013 | 53.9 | 284.9 | 159.3 | 730.7 | 0 | 726.8 | 74.9 | 370.2 | 3.12 |
| Pereira-de-Oliveira et al., 2013 | 53.7 | 284.9 | 159.5 | 730.7 | 0 | 646 | 149.8 | 370.2 | 3.12 |
| Pereira-de-Oliveira et al., 2013 | 53.3 | 284.9 | 160.1 | 730.7 | 0 | 565.2 | 224.6 | 370.2 | 3.12 |
| Pereira-de-Oliveira et al., 2013 | 53.0 | 284.9 | 160.1 | 730.7 | 0 | 484.5 | 299.4 | 370.2 | 3.12 |
| Nandanam et al., 2021 | 59.7 | 550 | 165 | 607 | 0 | 0 | 584 | 0 | 3.58 |
| Nandanam et al., 2021 | 36.0 | 385 | 148 | 729 | 0 | 0 | 702 | 165 | 7.15 |
| Nandanam et al., 2021 | 46.5 | 275 | 146 | 745 | 0 | 0 | 717 | 275 | 6.6 |
| Nandanam et al., 2021 | 14.2 | 165 | 172 | 757 | 0 | 0 | 728 | 385 | 5.5 |
| Nandanam et al., 2021 | 60.9 | 385 | 155 | 815 | 0 | 0 | 778 | 165 | 7.98 |
| Nandanam et al., 2021 | 58.7 | 275 | 162 | 789 | 0 | 0 | 753 | 275 | 7.15 |
| Nandanam et al., 2021 | 48.0 | 165 | 172 | 729 | 0 | 0 | 695 | 385 | 5.5 |
| Nandanam et al., 2021 | 43.2 | 509 | 221 | 734 | 0 | 0 | 701 | 41 | 4.95 |
| Nandanam et al., 2021 | 71.0 | 468 | 197 | 760 | 0 | 0 | 726 | 82 | 6.33 |
| Silva et al., 2016 | 35.3 | 384 | 216 | 964.8 | 0 | 643.2 | 0 | 96 | 2.88 |
| Silva et al., 2016 | 31.7 | 384 | 216 | 964.8 | 0 | 482.41 | 143.08 | 96 | 2.88 |
| Silva et al., 2016 | 32.1 | 384 | 216 | 964.8 | 0 | 321.6 | 286.16 | 96 | 2.88 |
| Silva et al., 2016 | 32.6 | 384 | 216 | 964.8 | 0 | 160.8 | 429.23 | 96 | 2.89 |
| Silva et al., 2016 | 29.8 | 384 | 216 | 964.8 | 0 | 0 | 572.29 | 96 | 2.9 |
| Larsen et al., 2019 | 33.6 | 445 | 219 | 717 | 0 | 0 | 611 | 125 | 1.14 |
| Larsen et al., 2019 | 42.9 | 512 | 235 | 664 | 0 | 0 | 565 | 145 | 1.31 |
| Larsen et al., 2019 | 31.6 | 430 | 226 | 717 | 0 | 0 | 611 | 121 | 1.1 |
| Larsen et al., 2019 | 36.4 | 445 | 219 | 717 | 0 | 0 | 611 | 126 | 2 |
| Larsen et al., 2019 | 35.8 | 436 | 223 | 717 | 0 | 0 | 611 | 123 | 1.62 |
| Larsen et al., 2019 | 40.8 | 485 | 229 | 685 | 0 | 0 | 584 | 137 | 1.8 |
| Larsen et al., 2019 | 38.9 | 475 | 233 | 685 | 0 | 0 | 584 | 134 | 1.22 |
| Larsen et al., 2019 | 37.7 | 453 | 231 | 698 | 0 | 0 | 595 | 128 | 2.03 |
| Larsen et al., 2019 | 45.0 | 506 | 238 | 664 | 0 | 0 | 565 | 143 | 2.27 |
| Larsen et al., 2019 | 41.6 | 495 | 241 | 664 | 0 | 0 | 565 | 140 | 1.61 |
| Revialla-Cuesta et al., 2022 | 44.7 | 300 | 185 | 1120 | 0 | 0 | 530 | 170 | 2.3 |
| Revialla-Cuesta et al., 2022 | 40.8 | 300 | 210 | 560 | 510 | 0 | 530 | 170 | 2.3 |
| Revialla-Cuesta et al., 2022 | 27.8 | 300 | 235 | 0 | 1010 | 0 | 530 | 170 | 2.3 |
| Revialla-Cuesta et al., 2022 | 50.4 | 425 | 185 | 1120 | 0 | 0 | 430 | 170 | 2.3 |
| Revialla-Cuesta et al., 2022 | 42.8 | 425 | 210 | 560 | 510 | 0 | 430 | 170 | 2.3 |
| Revialla-Cuesta et al., 2022 | 32.5 | 425 | 235 | 0 | 1010 | 0 | 430 | 170 | 2.3 |
| Revialla-Cuesta et al., 2022 | 45.5 | 300 | 185 | 1120 | 0 | 0 | 530 | 340 | 2.3 |
| Revialla-Cuesta et al., 2022 | 42.5 | 300 | 210 | 560 | 510 | 0 | 530 | 340 | 2.3 |
| Revialla-Cuesta et al., 2022 | 28.6 | 300 | 235 | 0 | 1010 | 0 | 530 | 340 | 2.3 |
| Revialla-Cuesta et al., 2022 | 54.5 | 425 | 185 | 1120 | 0 | 0 | 430 | 340 | 2.3 |
| Revialla-Cuesta et al., 2022 | 51.4 | 425 | 210 | 560 | 510 | 0 | 430 | 340 | 2.3 |
| Revialla-Cuesta et al., 2022 | 36.9 | 425 | 235 | 0 | 1010 | 0 | 430 | 340 | 2.3 |
| Revialla-Cuesta et al., 2022 | 35.9 | 300 | 185 | 1120 | 0 | 0 | 530 | 305 | 2.3 |
| Revialla-Cuesta et al., 2022 | 28.8 | 300 | 210 | 560 | 510 | 0 | 530 | 305 | 2.3 |
| Revialla-Cuesta et al., 2022 | 15.6 | 300 | 235 | 0 | 1010 | 0 | 530 | 305 | 2.3 |
| Revialla-Cuesta et al., 2022 | 41.8 | 425 | 185 | 1120 | 0 | 0 | 430 | 305 | 2.3 |
| Revialla-Cuesta et al., 2022 | 34.9 | 425 | 210 | 560 | 510 | 0 | 430 | 305 | 2.3 |
| Revialla-Cuesta et al., 2022 | 27.3 | 425 | 235 | 0 | 1010 | 0 | 430 | 305 | 2.3 |
| Singh et al., 2021 | 60.1 | 624 | 212 | 798.6 | 0 | 668.25 | 0 | 0 | 2.5 |
| Singh et al., 2021 | 58.0 | 624 | 212 | 798.6 | 0 | 334.12 | 306.9 | 0 | 2.5 |
| Singh et al., 2021 | 55.7 | 624 | 212 | 798.6 | 0 | 0 | 613.8 | 0 | 2.5 |
| Singh et al., 2021 | 37.5 | 288 | 196 | 798.6 | 0 | 668.25 | 0 | 288 | 1.56 |
| Singh et al., 2021 | 37.3 | 288 | 196 | 798.6 | 0 | 334.12 | 306.9 | 288 | 1.56 |
| Singh et al., 2021 | 36.4 | 288 | 196 | 798.6 | 0 | 0 | 613.8 | 288 | 1.56 |
| Singh et al., 2021 | 52.4 | 414 | 201 | 798.6 | 0 | 668.25 | 0 | 178 | 2.61 |
| Singh et al., 2021 | 50.8 | 414 | 201 | 798.6 | 0 | 334.12 | 306.9 | 178 | 2.61 |
| Singh et al., 2021 | 50.4 | 414 | 201 | 798.6 | 0 | 0 | 613.8 | 178 | 2.61 |
| Singh et al., 2021 | 55.1 | 417 | 203 | 798.6 | 0 | 668.25 | 0 | 179 | 2.62 |
| Singh et al., 2021 | 53.2 | 417 | 203 | 798.6 | 0 | 334.12 | 306.9 | 179 | 2.62 |
| Singh et al., 2021 | 52.4 | 417 | 203 | 798.6 | 0 | 0 | 613.8 | 179 | 2.62 |
| Sua-iam & Makul., 2013 | 26.8 | 450 | 171 | 922 | 0 | 804 | 0 | 0 | 0 |
| Sua-iam & Makul., 2013 | 30.6 | 450 | 171 | 692 | 230 | 804 | 0 | 0 | 0 |
| Sua-iam & Makul., 2013 | 35.7 | 450 | 171 | 461 | 461 | 804 | 0 | 0 | 0 |
| Sua-iam & Makul., 2013 | 55.3 | 450 | 171 | 230 | 692 | 804 | 0 | 0 | 0 |
| Sua-iam & Makul., 2013 | 47.1 | 450 | 171 | 0 | 922 | 804 | 0 | 0 | 0 |
| Sua-iam & Makul., 2013 | 22.9 | 450 | 202 | 922 | 0 | 804 | 0 | 0 | 0 |
| Sua-iam & Makul., 2013 | 24.2 | 450 | 202 | 692 | 230 | 804 | 0 | 0 | 0 |
| Sua-iam & Makul., 2013 | 32.5 | 450 | 202 | 461 | 461 | 804 | 0 | 0 | 0 |
| Sua-iam & Makul., 2013 | 45.9 | 450 | 202 | 230 | 692 | 804 | 0 | 0 | 0 |
| Sua-iam & Makul., 2013 | 40.8 | 450 | 202 | 0 | 922 | 804 | 0 | 0 | 0 |
| Sua-iam & Makul., 2013 | 30.6 | 550 | 209 | 813 | 0 | 708 | 0 | 0 | 0 |
| Sua-iam & Makul., 2013 | 33.1 | 550 | 209 | 610 | 203 | 708 | 0 | 0 | 0 |
| Sua-iam & Makul., 2013 | 38.2 | 550 | 209 | 407 | 406 | 708 | 0 | 0 | 0 |
| Sua-iam & Makul., 2013 | 59.9 | 550 | 209 | 203 | 610 | 708 | 0 | 0 | 0 |
| Sua-iam & Makul., 2013 | 50.3 | 550 | 209 | 0 | 813 | 708 | 0 | 0 | 0 |
| Sua-iam & Makul., 2013 | 29.3 | 550 | 248 | 813 | 0 | 708 | 0 | 0 | 0 |
| Sua-iam & Makul., 2013 | 30.6 | 550 | 248 | 610 | 203 | 708 | 0 | 0 | 0 |
| Sua-iam & Makul., 2013 | 35.7 | 550 | 248 | 407 | 406 | 708 | 0 | 0 | 0 |
| Sua-iam & Makul., 2013 | 48.4 | 550 | 248 | 203 | 610 | 708 | 0 | 0 | 0 |
| Sua-iam & Makul., 2013 | 45.9 | 550 | 248 | 0 | 813 | 708 | 0 | 0 | 0 |
| Wang et al., 2020 | 72.3 | 418 | 158.4 | 965 | 0 | 860 | 0 | 22 | 6.5 |
| Wang et al., 2020 | 58.5 | 418 | 158.4 | 868.5 | 40.3 | 860 | 0 | 22 | 6.5 |
| Wang et al., 2020 | 56 | 418 | 158.4 | 868.5 | 56.6 | 860 | 0 | 22 | 6.5 |
| Wang et al., 2020 | 50.1 | 418 | 158.4 | 820.3 | 76.3 | 860 | 0 | 22 | 6.5 |
| Wang et al., 2020 | 32.2 | 418 | 158.4 | 723.7 | 116.5 | 860 | 0 | 22 | 6.5 |
| Yu et al., 2014 | 58.2 | 385 | 165 | 763 | 0 | 895 | 0 | 165 | 4.04 |
| Yu et al., 2014 | 57.3 | 385 | 165 | 763 | 0 | 448 | 448 | 165 | 4.04 |
| Yu et al., 2014 | 52 | 350 | 200 | 829 | 0 | 0 | 829 | 200 | 3.68 |
| Yu et al., 2021 | 64 | 477 | 164 | 736.2 | 0 | 798 | 0 | 113 | 0 |
| Yu et al., 2021 | 51.5 | 449 | 164 | 797.5 | 0 | 558 | 240 | 137 | 2.93 |
| Yu et al., 2021 | 49 | 419 | 164 | 841.1 | 0 | 320 | 478 | 160 | 5.79 |
| Yu et al., 2021 | 40.5 | 391 | 164 | 936.2 | 0 | 798 | 0 | 185 | 8.64 |
| Yu et al., 2021 | 53.2 | 395 | 176 | 936.2 | 0 | 558 | 240 | 151 | 0 |
| Yu et al., 2021 | 48 | 366 | 176 | 841.1 | 0 | 798 | 0 | 173 | 2.7 |
| Yu et al., 2021 | 46.2 | 449 | 176 | 797.5 | 0 | 0 | 798 | 106 | 5.55 |
| Yu et al., 2021 | 43.2 | 421 | 176 | 736.2 | 0 | 320 | 478 | 128 | 8.24 |
| Yu et al., 2021 | 56 | 335 | 193 | 797.5 | 0 | 320 | 478 | 158 | 0 |
| Yu et al., 2021 | 44.7 | 361 | 193 | 736.2 | 0 | 798 | 0 | 138 | 2.5 |
| Yu et al., 2021 | 38.5 | 386 | 193 | 936.2 | 0 | 0 | 798 | 117 | 5.03 |
| Yu et al., 2021 | 41.8 | 409 | 193 | 841.1 | 0 | 558 | 240 | 97 | 7.59 |
| Yu et al., 2021 | 29.6 | 349 | 210 | 841.1 | 0 | 0 | 798 | 106 | 0 |
| Yu et al., 2021 | 41.8 | 372 | 210 | 936.2 | 0 | 320 | 478 | 88 | 2.3 |
| Yu et al., 2021 | 36.5 | 305 | 210 | 736.2 | 0 | 558 | 240 | 144 | 4.5 |
| Yu et al., 2021 | 33.4 | 326 | 210 | 797.5 | 0 | 798 | 0 | 125 | 6.8 |
| Santos et al., 2017 | 42.9 | 270 | 187 | 698 | 0 | 787 | 0 | 306 | 3 |
| Santos et al., 2017 | 38.9 | 270 | 187 | 523 | 167 | 591 | 193 | 306 | 3 |
| Santos et al., 2017 | 37 | 270 | 187 | 349 | 333 | 394 | 385 | 306 | 3 |
| Santos et al., 2017 | 34 | 270 | 187 | 698 | 0 | 0 | 770 | 306 | 3 |
| Santos et al., 2017 | 29.1 | 270 | 187 | 0 | 667 | 787 | 0 | 306 | 3 |
| Santos et al., 2017 | 78.7 | 437 | 188 | 695 | 0 | 787 | 0 | 177 | 4 |
| Santos et al., 2017 | 77.6 | 437 | 188 | 521 | 156 | 591 | 184 | 177 | 4 |
| Santos et al., 2017 | 75.6 | 437 | 188 | 347 | 312 | 394 | 369 | 177 | 4 |
| Santos et al., 2017 | 74.2 | 437 | 188 | 695 | 0 | 0 | 737 | 177 | 4 |
| Santos et al., 2017 | 69.3 | 437 | 188 | 0 | 624 | 787 | 0 | 177 | 4 |
| Omrane et al., 2016 | 31.6 | 449 | 189 | 435 | 435 | 435 | 435 | 449 | 4.04 |
| Omrane et al., 2016 | 29.7 | 426 | 189 | 435 | 435 | 435 | 435 | 449 | 3.83 |
| Omrane et al., 2016 | 24.2 | 404 | 189 | 435 | 435 | 435 | 435 | 449 | 3.64 |
| Omrane et al., 2016 | 23.3 | 381 | 189 | 435 | 435 | 435 | 435 | 449 | 3.43 |
| Omrane et al., 2016 | 21.4 | 359 | 189 | 435 | 435 | 435 | 435 | 449 | 3.23 |
| Wang et al., 2019 | 57.8 | 401.47 | 198.96 | 836.46 | 0 | 768.5 | 0 | 122.11 | 2.09 |
| Wang et al., 2019 | 49.6 | 401.47 | 198.96 | 836.46 | 0 | 384.25 | 384.25 | 122.11 | 2.09 |
| Wang et al., 2019 | 46.3 | 401.47 | 198.96 | 836.46 | 0 | 0 | 768.5 | 122.11 | 2.09 |
| Guo et al., 2020 | 53.45 | 520 | 182 | 785 | 0 | 867 | 0 | 0 | 4.02 |
| Guo et al., 2020 | 46.54 | 520 | 182 | 785 | 0 | 433 | 416.4 | 0 | 4.16 |
| Guo et al., 2020 | 18.04 | 260 | 182 | 785 | 0 | 433 | 416.4 | 260 | 5.72 |
| Guo et al., 2020 | 33.51 | 260 | 182 | 785 | 0 | 433 | 416.4 | 260 | 5.43 |
| Guo et al., 2020 | 40.31 | 260 | 182 | 785 | 0 | 433 | 416.4 | 260 | 6.76 |
| Guo et al., 2020 | 7.17 | 130 | 182 | 785 | 0 | 433 | 416.4 | 390 | 5.94 |
| Guo et al., 2020 | 19.66 | 130 | 182 | 785 | 0 | 433 | 416.4 | 390 | 4.68 |
| Guo et al., 2020 | 35.54 | 130 | 182 | 785 | 0 | 433 | 416.4 | 390 | 6.8 |
| Guo et al., 2020 | 43.89 | 520 | 182 | 785 | 0 | 0 | 832 | 0 | 4.72 |
| Guo et al., 2020 | 21 | 260 | 182 | 785 | 0 | 0 | 832 | 260 | 4.78 |
| Guo et al., 2020 | 38.38 | 260 | 182 | 785 | 0 | 0 | 832 | 260 | 5.67 |
| Guo et al., 2020 | 49.44 | 260 | 182 | 785 | 0 | 0 | 832 | 260 | 6.86 |
| Guo et al., 2020 | 13.64 | 130 | 182 | 785 | 0 | 0 | 832 | 390 | 7.4 |
| Guo et al., 2020 | 30.84 | 130 | 182 | 785 | 0 | 0 | 832 | 390 | 7.28 |
| Guo et al., 2020 | 42.75 | 130 | 182 | 785 | 0 | 0 | 832 | 390 | 7.57 |
| Guo et al., 2020 | 29.81 | 455 | 182 | 785 | 0 | 0 | 832 | 0 | 3.19 |
| Guo et al., 2020 | 13.89 | 113.7 | 182 | 785 | 0 | 0 | 832 | 341.23 | 4.01 |
| Guo et al., 2020 | 18.35 | 113.7 | 182 | 785 | 0 | 0 | 832 | 341.23 | 1.37 |
| Guo et al., 2020 | 26.23 | 113.7 | 182 | 785 | 0 | 0 | 832 | 341.23 | 3.19 |
| Guo et al., 2020 | 19.75 | 404 | 182 | 785 | 0 | 0 | 832 | 0 | 2.83 |
| Guo et al., 2020 | 9.63 | 101 | 182 | 785 | 0 | 0 | 832 | 303 | 3.2 |
| Guo et al., 2020 | 12.07 | 101 | 182 | 785 | 0 | 0 | 832 | 303 | 1.21 |
| Guo et al., 2020 | 18.86 | 101 | 182 | 785 | 0 | 0 | 832 | 303 | 3.63 |
| Gesoglu et al., 2015 | 77.96 | 427.5 | 171 | 765 | 0 | 859.8 | 0 | 142.5 | 6.29 |
| Gesoglu et al., 2015 | 81.4 | 370.5 | 171 | 757.5 | 0 | 851.4 | 0 | 199.5 | 7 |
| Gesoglu et al., 2015 | 66.63 | 360 | 206.4 | 773.4 | 0 | 869.3 | 0 | 120 | 3.11 |
| Gesoglu et al., 2015 | 72.47 | 312 | 206.4 | 767.2 | 0 | 862.3 | 0 | 167 | 4.55 |
| Gesoglu et al., 2015 | 68.67 | 427.5 | 171 | 765 | 0 | 0 | 749.2 | 142.5 | 4.95 |
| Gesoglu et al., 2015 | 70.39 | 370.5 | 171 | 757.5 | 0 | 0 | 741.9 | 199.5 | 6.26 |
| Gesoglu et al., 2015 | 55.38 | 360 | 206.4 | 773.4 | 0 | 0 | 757.5 | 120 | 2.55 |
| Gesoglu et al., 2015 | 63.89 | 312 | 206.4 | 767.2 | 0 | 0 | 751.3 | 168 | 4.09 |
| Gesoglu et al., 2015 | 61.97 | 427.5 | 171 | 0 | 667 | 859.8 | 0 | 142.5 | 4.51 |
| Gesoglu et al., 2015 | 64.61 | 370.5 | 171 | 0 | 660.5 | 851.4 | 0 | 199.5 | 4.2 |
| Gesoglu et al., 2015 | 48.69 | 360 | 206.4 | 0 | 674.4 | 869.3 | 0 | 120 | 1.82 |
| Gesoglu et al., 2015 | 61.04 | 312 | 206.4 | 0 | 668.9 | 862.3 | 0 | 168 | 2.7 |
| Gesoglu et al., 2015 | 55.76 | 427.5 | 171 | 0 | 667 | 0 | 749.2 | 142.5 | 3.44 |
| Gesoglu et al., 2015 | 57.41 | 370.5 | 171 | 0 | 660.5 | 0 | 741.9 | 199.5 | 3.79 |
| Gesoglu et al., 2015 | 46.04 | 360 | 206.4 | 0 | 674.4 | 0 | 757.5 | 120 | 1.78 |
| Gesoglu et al., 2015 | 52.92 | 312 | 206.4 | 0 | 668.9 | 0 | 751.3 | 168 | 2.62 |
| Behera et al., 2019 | 60.76 | 313.54 | 174.06 | 927.75 | 0 | 749.7 | 0 | 209.03 | 5.23 |
| Behera et al., 2019 | 55.76 | 313.54 | 174.065 | 463.87 | 364.85 | 749.7 | 0 | 209.03 | 5.23 |
| Behera et al., 2019 | 44.54 | 313.54 | 174.06 | 0 | 729.69 | 749.7 | 0 | 209.03 | 5.23 |
| Behera et al., 2019 | 44.54 | 315.11 | 174.06 | 927.75 | 0 | 749.7 | 0 | 210.08 | 3.15 |
| Behera et al., 2019 | 38.41 | 315.11 | 174.065 | 0 | 729.75 | 749.7 | 0 | 210.08 | 4.2 |
| Bahrami et al., 2020 | 46 | 400 | 150 | 1200 | 0 | 530 | 0 | 100 | 3.5 |
| Bahrami et al., 2020 | 38.99 | 400 | 167 | 1200 | 0 | 530 | 0 | 100 | 3.8 |
| Bahrami et al., 2020 | 38.99 | 400 | 167 | 1200 | 0 | 530 | 0 | 100 | 3.8 |
| Bahrami et al., 2020 | 36.7 | 400 | 167 | 900 | 300 | 530 | 0 | 100 | 3.8 |
| Bahrami et al., 2020 | 32.8 | 400 | 167 | 600 | 600 | 530 | 0 | 100 | 3.8 |
| Bahrami et al., 2020 | 29.6 | 400 | 167 | 300 | 900 | 530 | 0 | 100 | 3.8 |
| Bahrami et al., 2020 | 24.8 | 400 | 167 | 0 | 1200 | 530 | 0 | 100 | 3.8 |
| Bahrami et al., 2020 | 37 | 400 | 167 | 1200 | 0 | 397.5 | 132.5 | 100 | 3.8 |
| Bahrami et al., 2020 | 33.51 | 400 | 167 | 1200 | 0 | 265 | 265 | 100 | 3.8 |
| Bahrami et al., 2020 | 31.36 | 400 | 167 | 1200 | 0 | 132.5 | 397.5 | 100 | 3.8 |
| Bahrami et al., 2020 | 25.83 | 400 | 167 | 1200 | 0 | 0 | 530 | 100 | 3.8 |
| Bahrami et al., 2020 | 38 | 380 | 167 | 900 | 300 | 530 | 0 | 100 | 3.8 |
| Bahrami et al., 2020 | 39.6 | 370 | 167 | 900 | 300 | 530 | 0 | 100 | 3.8 |
| Bahrami et al., 2020 | 42.1 | 360 | 167 | 900 | 300 | 530 | 0 | 100 | 3.8 |
| Bahrami et al., 2020 | 38.7 | 380 | 167 | 1200 | 0 | 397.5 | 132.5 | 100 | 3.8 |
| Bahrami et al., 2020 | 40.3 | 370 | 167 | 1200 | 0 | 397.5 | 132.5 | 100 | 3.8 |
| Bahrami et al., 2020 | 42 | 360 | 167 | 1200 | 0 | 397.5 | 132.5 | 100 | 3.8 |
| Mahakavi & Chitra., 2020 | 40.7 | 500 | 172 | 919 | 0 | 803 | 0 | 31 | 37.5 |
| Mahakavi & Chitra., 2020 | 36.11 | 500 | 172 | 919 | 0 | 602.25 | 200.75 | 31 | 37.5 |
| Mahakavi & Chitra., 2020 | 33.65 | 500 | 172 | 919 | 0 | 401.5 | 401.5 | 31 | 37.5 |
| Mahakavi & Chitra., 2020 | 28.65 | 500 | 172 | 919 | 0 | 200.75 | 602.25 | 31 | 37.5 |
| Mahakavi & Chitra., 2020 | 25.37 | 500 | 172 | 919 | 0 | 0 | 803 | 31 | 37.5 |
| Mahakavi & Chitra., 2020 | 42.81 | 500 | 172 | 689.25 | 229.75 | 803 | 0 | 31 | 37.5 |
| Mahakavi & Chitra., 2020 | 39.27 | 500 | 172 | 459.5 | 459.5 | 803 | 0 | 31 | 37.5 |
| Mahakavi & Chitra., 2020 | 36.2 | 500 | 172 | 229.75 | 689.25 | 803 | 0 | 31 | 37.5 |
| Mahakavi & Chitra., 2020 | 32.19 | 500 | 172 | 0 | 919 | 803 | 0 | 31 | 37.5 |
| Mahakavi & Chitra., 2020 | 28.61 | 500 | 172 | 689.25 | 229.75 | 602.25 | 200.75 | 31 | 37.5 |
| Mahakavi & Chitra., 2020 | 45.65 | 500 | 172 | 689.25 | 229.75 | 401.5 | 401.5 | 31 | 37.5 |
| Mahakavi & Chitra., 2020 | 40.62 | 500 | 172 | 689.25 | 229.75 | 200.75 | 602.25 | 31 | 37.5 |
| Mahakavi & Chitra., 2020 | 38.08 | 500 | 172 | 689.25 | 229.75 | 0 | 803 | 31 | 37.5 |
| Mahakavi & Chitra., 2020 | 33.12 | 500 | 172 | 459.5 | 459.5 | 602.25 | 200.75 | 31 | 37.5 |
| Mahakavi & Chitra., 2020 | 29.76 | 500 | 172 | 459.5 | 459.5 | 401.5 | 401.5 | 31 | 37.5 |
| Mahakavi & Chitra., 2020 | 42.65 | 500 | 172 | 459.5 | 459.5 | 200.75 | 602.25 | 31 | 37.5 |
| Mahakavi & Chitra., 2020 | 37.01 | 500 | 172 | 459.5 | 459.5 | 0 | 803 | 31 | 37.5 |
| Mahakavi & Chitra., 2020 | 34.89 | 500 | 172 | 229.75 | 689.25 | 602.25 | 200.75 | 31 | 37.5 |
| Mahakavi & Chitra., 2020 | 29.71 | 500 | 172 | 229.75 | 689.25 | 401.5 | 401.5 | 31 | 37.5 |
| Mahakavi & Chitra., 2020 | 25.99 | 500 | 172 | 229.75 | 689.25 | 200.75 | 602.25 | 31 | 37.5 |
| Mahakavi & Chitra., 2020 | 33.79 | 500 | 172 | 229.75 | 689.25 | 0 | 803 | 31 | 37.5 |
| Mahakavi & Chitra., 2020 | 31.78 | 500 | 172 | 0 | 919 | 602.25 | 200.75 | 31 | 37.5 |
| Mahakavi & Chitra., 2020 | 29.24 | 500 | 172 | 0 | 919 | 401.5 | 401.5 | 31 | 37.5 |
| Mahakavi & Chitra., 2020 | 24.65 | 500 | 172 | 0 | 919 | 200.75 | 602.25 | 31 | 37.5 |
| Mahakavi & Chitra., 2020 | 20.97 | 500 | 172 | 0 | 919 | 0 | 803 | 31 | 37.5 |
| Tuyan et al., 2014 | 52.3 | 315 | 194 | 738 | 0 | 745 | 0 | 297 | 4.9 |
| Tuyan et al., 2014 | 42.2 | 315 | 216 | 713 | 0 | 720 | 0 | 292 | 3.3 |
| Tuyan et al., 2014 | 37.2 | 315 | 239 | 688 | 0 | 694 | 0 | 286 | 2.2 |
| Tuyan et al., 2014 | 54.7 | 315 | 194 | 738 | 0 | 596 | 149 | 297 | 5.5 |
| Tuyan et al., 2014 | 44 | 315 | 216 | 713 | 0 | 576 | 144 | 292 | 3.4 |
| Tuyan et al., 2014 | 37.7 | 315 | 239 | 688 | 0 | 555 | 139 | 286 | 2.4 |
| Tuyan et al., 2014 | 57.2 | 315 | 194 | 738 | 0 | 447 | 298 | 297 | 5.7 |
| Tuyan et al., 2014 | 44.3 | 315 | 216 | 713 | 0 | 432 | 288 | 292 | 3.5 |
| Tuyan et al., 2014 | 38.2 | 315 | 239 | 688 | 0 | 416 | 278 | 286 | 2.7 |
| Tuyan et al., 2014 | 51.1 | 315 | 194 | 738 | 0 | 298 | 447 | 297 | 6.2 |
| Tuyan et al., 2014 | 40.9 | 315 | 216 | 713 | 0 | 288 | 432 | 292 | 3.7 |
| Tuyan et al., 2014 | 35.8 | 315 | 239 | 688 | 0 | 278 | 416 | 286 | 3 |
| Zhou et al., 2013 | 59.11 | 550 | 165 | 720 | 0 | 995 | 0 | 0 | 8.8 |
| Zhou et al., 2013 | 56.78 | 315 | 165 | 833 | 0 | 0 | 882 | 200 | 8.8 |
| Zhou et al., 2013 | 61.18 | 346.5 | 165 | 833 | 0 | 0 | 882 | 220 | 8.8 |
| Zhou et al., 2013 | 64.54 | 378 | 180 | 833 | 0 | 0 | 882 | 240 | 9.6 |
| Zhou et al., 2013 | 66.35 | 378 | 174 | 833 | 0 | 0 | 882 | 240 | 9.6 |
| Zhou et al., 2013 | 68.47 | 378 | 168 | 833 | 0 | 0 | 882 | 240 | 9.6 |
| Yu et al., 2014 | 58.2 | 385 | 171 | 763 | 0 | 895 | 0 | 165 | 0.5775 |
| Yu et al., 2014 | 57.3 | 385 | 171 | 763 | 0 | 448 | 448 | 165 | 0.5775 |
| Yu et al., 2014 | 52 | 350 | 176 | 829 | 0 | 0 | 829 | 200 | 0.525 |
| Sadeghi-Nik et al., 2018 | 52 | 427.3 | 183 | 930 | 0 | 712 | 0 | 94.006 | 7 |
| Sadeghi-Nik et al., 2018 | 43.1 | 427.3 | 183 | 744 | 186 | 569.6 | 142.4 | 94.006 | 7 |
| Sadeghi-Nik et al., 2018 | 41 | 427.3 | 183 | 558 | 372 | 427.2 | 284.8 | 94.006 | 7 |
| Sadeghi-Nik et al., 2018 | 40.1 | 427.3 | 183 | 372 | 558 | 284.8 | 427.2 | 94.006 | 7 |
| Sadeghi-Nik et al., 2018 | 38.1 | 427.3 | 183 | 186 | 744 | 142.4 | 569.6 | 94.006 | 7 |
| Sadeghi-Nik et al., 2018 | 30 | 427.3 | 183 | 0 | 930 | 0 | 712 | 94.006 | 7 |
| Sadeghi-Nik et al., 2018 | 74.1 | 427.3 | 183 | 930 | 0 | 712 | 0 | 94.006 | 7 |
| Sadeghi-Nik et al., 2018 | 55 | 427.3 | 183 | 744 | 186 | 569.6 | 142.4 | 94.006 | 7 |
| Sadeghi-Nik et al., 2018 | 52.1 | 427.3 | 183 | 558 | 372 | 427.2 | 284.8 | 94.006 | 7 |
| Sadeghi-Nik et al., 2018 | 51 | 427.3 | 183 | 372 | 558 | 284.8 | 427.2 | 94.006 | 7 |
| Sadeghi-Nik et al., 2018 | 50.5 | 427.3 | 183 | 186 | 744 | 142.4 | 569.6 | 94.006 | 7 |
| Sadeghi-Nik et al., 2018 | 40 | 427.3 | 183 | 0 | 930 | 0 | 712 | 94.006 | 7 |
| Babalola et al., 2020 | 30.6 | 433 | 195 | 532 | 0 | 990 | 0 | 0 | 3.464 |
| Babalola et al., 2020 | 31.7 | 160 | 130 | 770 | 0 | 0 | 1115 | 127 | 3.464 |
| Babalola et al., 2020 | 33.5 | 144 | 129 | 770 | 0 | 0 | 1115 | 143 | 3.464 |
| Babalola et al., 2020 | 35.8 | 127 | 127 | 770 | 0 | 0 | 1115 | 159 | 3.464 |
| Babalola et al., 2020 | 37 | 111 | 128 | 770 | 0 | 0 | 1115 | 174 | 3.464 |
| Babalola et al., 2020 | 39.8 | 94 | 128 | 770 | 0 | 0 | 1115 | 190 | 3.464 |
| Babalola et al., 2020 | 39.2 | 78 | 128 | 770 | 0 | 0 | 1115 | 205 | 3.464 |
| Babalola et al., 2020 | 41.4 | 158 | 105 | 770 | 0 | 0 | 1115 | 195 | 3.464 |
| Babalola et al., 2020 | 38 | 140 | 114 | 770 | 0 | 0 | 1115 | 187 | 3.464 |
| Babalola et al., 2020 | 37.4 | 124 | 122 | 770 | 0 | 0 | 1115 | 180 | 3.464 |
| Babalola et al., 2020 | 37 | 111 | 128 | 770 | 0 | 0 | 1115 | 174 | 3.464 |
| Babalola et al., 2020 | 33 | 99 | 133 | 770 | 0 | 0 | 1115 | 169 | 3.464 |
| Pereira-de-Oliveira et al., 2014 | 54 | 284.9 | 161.3 | 730.8 | 0 | 807.9 | 0 | 370.2 | 3.4 |
| Pereira-de-Oliveira et al., 2014 | 54 | 284.9 | 163.2 | 730.8 | 0 | 646.3 | 149.6 | 370.2 | 4.8 |
| Pereira-de-Oliveira et al., 2014 | 53 | 284.9 | 160.7 | 730.8 | 0 | 484.8 | 299.3 | 370.2 | 4.6 |
| Pereira-de-Oliveira et al., 2014 | 51 | 284.9 | 162.4 | 730.8 | 0 | 0 | 808.5 | 370.2 | 6 |
| Poongodi et al., 2021 | 43.33 | 420 | 180 | 810 | 0 | 1170 | 0 | 0 | 0 |
| Poongodi et al., 2021 | 46.67 | 294 | 180 | 810 | 0 | 1170 | 0 | 126 | 0 |
| Poongodi et al., 2021 | 48.03 | 294 | 180 | 810 | 0 | 936 | 234 | 126 | 0 |
| Poongodi et al., 2021 | 49.48 | 294 | 180 | 810 | 0 | 702 | 468 | 126 | 0 |
| Poongodi et al., 2021 | 43.13 | 294 | 180 | 810 | 0 | 468 | 702 | 126 | 0 |
| Poongodi et al., 2021 | 52.16 | 294 | 168 | 910 | 0 | 900 | 0 | 126 | 6 |
| Poongodi et al., 2021 | 51.33 | 294 | 168 | 910 | 0 | 720 | 180 | 126 | 6 |
| Poongodi et al., 2021 | 50.07 | 294 | 168 | 910 | 0 | 540 | 360 | 126 | 6 |
| Poongodi et al., 2021 | 46.22 | 294 | 168 | 910 | 0 | 360 | 540 | 126 | 6 |
| Revathi et al., 2013 | 36.2 | 300 | 191 | 761 | 0 | 875 | 0 | 169 | 4 |
| Revathi et al., 2013 | 35.4 | 300 | 191 | 761 | 0 | 656 | 206 | 169 | 4.2 |
| Revathi et al., 2013 | 34.7 | 300 | 191 | 761 | 0 | 437 | 413 | 169 | 4.2 |
| Revathi et al., 2013 | 32.8 | 300 | 191 | 761 | 0 | 219 | 619 | 169 | 4.3 |
| Revathi et al., 2013 | 30.3 | 300 | 194 | 761 | 0 | 0 | 825 | 158 | 4.3 |
| Senas et al., 2016 | 51.2 | 415 | 166 | 1065 | 0 | 750 | 0 | 0 | 4.15 |
| Senas et al., 2016 | 48.1 | 415 | 166 | 1065 | 0 | 375 | 333 | 0 | 4.98 |
| Senas et al., 2016 | 45.6 | 415 | 166 | 852 | 195 | 375 | 333 | 0 | 5.81 |
| Senas et al., 2016 | 47.6 | 415 | 166 | 1065 | 0 | 750 | 0 | 0 | 2.91 |
| Senas et al., 2016 | 46.6 | 415 | 166 | 1065 | 0 | 375 | 333 | 0 | 5.4 |
| Senas et al., 2016 | 47 | 415 | 166 | 852 | 195 | 375 | 333 | 0 | 6.23 |
| Nili et al., 2019 | 46.3 | 418 | 184 | 1172 | 0 | 335 | 0 | 167 | 3.334 |
| Nili et al., 2019 | 32.1 | 418 | 184 | 1160 | 0 | 0 | 331 | 166 | 3.8874 |
| Nili et al., 2019 | 35.2 | 418 | 184 | 580 | 580 | 332 | 0 | 166 | 3.8874 |
| Nili et al., 2019 | 38.2 | 418 | 184 | 574 | 574 | 0 | 328 | 164 | 3.8874 |
| Nili et al., 2019 | 46.4 | 385 | 184 | 1172 | 0 | 335 | 0 | 200 | 3.465 |
| Nili et al., 2019 | 52.9 | 385 | 184 | 1160 | 0 | 0 | 331 | 199 | 3.85 |
| Nili et al., 2019 | 32.5 | 385 | 184 | 580 | 580 | 332 | 0 | 199 | 4.235 |
| Nili et al., 2019 | 31.8 | 385 | 184 | 574 | 574 | 0 | 328 | 197 | 4.62 |
| Barroqueiro et al., 2020 | 81.9 | 437 | 193 | 646 | 0 | 787 | 0 | 201 | 8 |
| Barroqueiro et al., 2020 | 80.7 | 437 | 193 | 485 | 145 | 591 | 184 | 201 | 8 |
| Barroqueiro et al., 2020 | 79.5 | 437 | 193 | 323 | 290 | 394 | 369 | 201 | 8 |
| Barroqueiro et al., 2020 | 75 | 437 | 193 | 0 | 581 | 0 | 737 | 201 | 8 |
| Barroqueiro et al., 2020 | 80.3 | 437 | 193 | 646 | 0 | 0 | 737 | 201 | 8 |
| Barroqueiro et al., 2020 | 78 | 437 | 193 | 0 | 581 | 787 | 0 | 201 | 8 |
| Fiol et al., 2018 | 49.09 | 250 | 112 | 650 | 0 | 1150 | 0 | 320 | 1.8 |
| Fiol et al., 2018 | 49.98 | 250 | 112 | 650 | 0 | 920 | 250 | 320 | 1.8 |
| Fiol et al., 2018 | 55.64 | 250 | 112 | 670 | 0 | 540 | 540 | 320 | 2.25 |
| Fiol et al., 2018 | 56.75 | 250 | 112 | 720 | 0 | 0 | 1040 | 320 | 2.85 |
| Fiol et al., 2018 | 58.3 | 290 | 112 | 650 | 0 | 1150 | 0 | 300 | 1.8 |
| Fiol et al., 2018 | 60.25 | 290 | 112 | 650 | 0 | 920 | 250 | 300 | 1.8 |
| Fiol et al., 2018 | 58.52 | 290 | 112 | 670 | 0 | 540 | 540 | 300 | 2.25 |
| Fiol et al., 2018 | 70.56 | 290 | 112 | 720 | 0 | 0 | 1040 | 300 | 2.85 |
| Fiol et al., 2018 | 63.36 | 320 | 112 | 650 | 0 | 1150 | 0 | 280 | 1.8 |
| Fiol et al., 2018 | 64.13 | 320 | 112 | 650 | 0 | 920 | 250 | 280 | 1.8 |
| Fiol et al., 2018 | 66.82 | 320 | 112 | 720 | 0 | 540 | 540 | 280 | 2.25 |
| Fiol et al., 2018 | 72.81 | 320 | 112 | 720 | 0 | 0 | 1040 | 280 | 2.85 |
