# Supplementary figures and images for: Artificial neural network, machine learning modelling of compressive strength of recycled coarse aggregate based self-compacting concrete
Source: PLoS One. 2024 May 13;19(5):e0303101. doi: 10.1371/journal.pone.0303101 (PMC11090367; doi:10.1371/journal.pone.0303101)

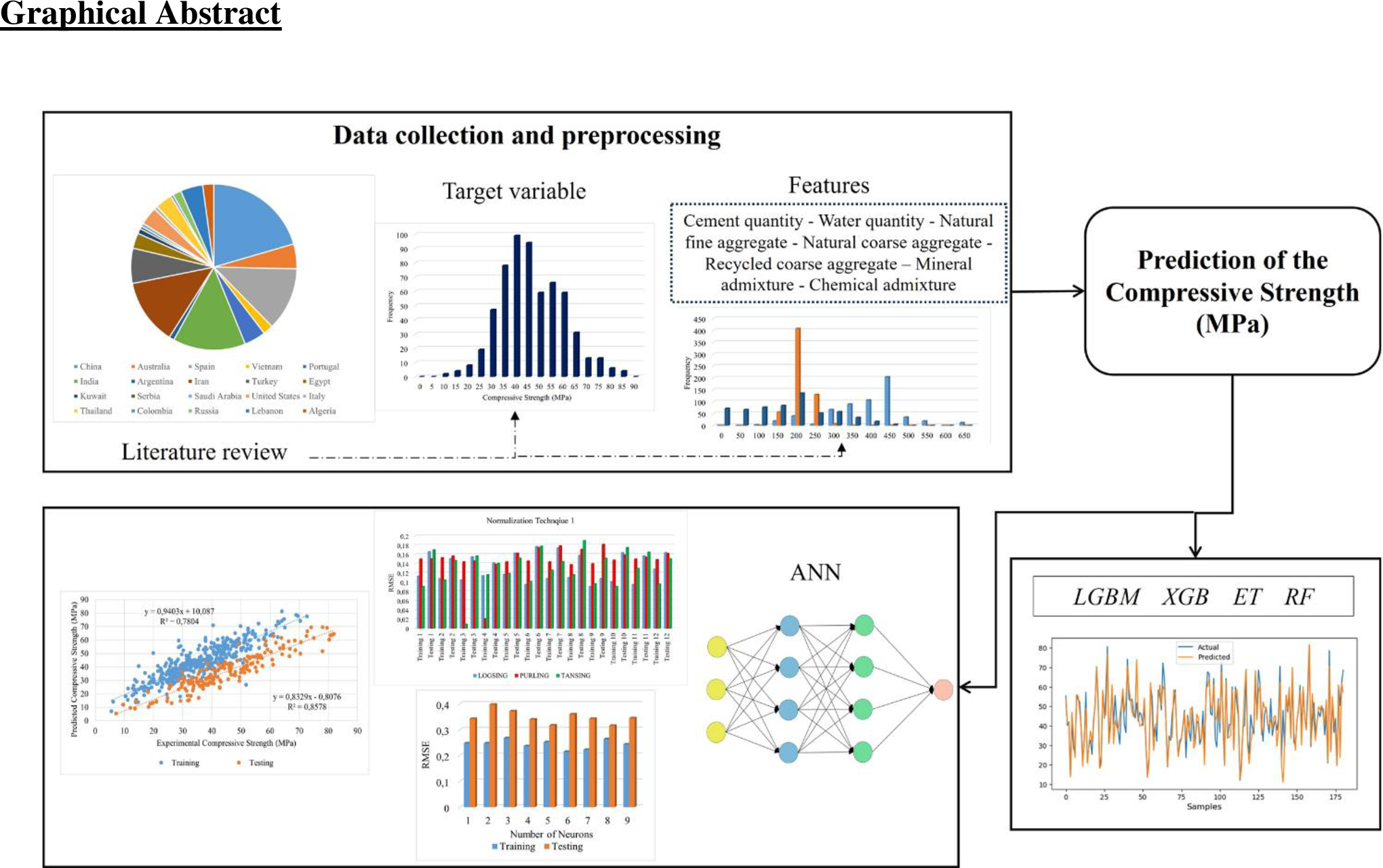

Supplement: S1 Graphical abstract — (TIF) [file pone.0303101.s002.tif]
